# Development and validation of a *Xanthomonas axonopodis *pv. *citri *DNA microarray platform (*XACarray*) generated from the shotgun libraries previously used in the sequencing of this bacterial genome

**DOI:** 10.1186/1756-0500-3-150

**Published:** 2010-05-27

**Authors:** Leandro M Moreira, Marcelo L de Laia, Robson F de Souza, Paulo A Zaini, Ana CR da Silva, Aline M da Silva, Jesus A Ferro

**Affiliations:** 1 Departamento de Ciências Biológicas (DECBI), Instituto de Ciências Exatas e Biológicas, Universidade Federal de Ouro Preto, Campus Morro do Cruzeiro, Ouro Preto, MG, Brazil; 2 Núcleo de Pesquisas em Ciências Biológicas (NUPEB), Universidade Federal de Ouro Preto, Ouro Preto, MG, Brazil; 3 Departamento de Bioquímica, Instituto de Química, Universidade de São Paulo, São Paulo, SP, Brazil; 4 Departamento de Engenharia Florestal, Faculdade de Ciências Agrárias, Universidade Federal dos Vales do Jequitinhonha e Mucuri, Diamantina, MG, Brazil; 5 Departamento de Tecnologia, Faculdade de Ciências Agrárias e Veterinárias de Jaboticabal, UNESP - Univ. Estadual Paulista, Jaboticabal, SP, Brazil; 6 Alellyx Applied Genomics, Rua James Clerk Maxwell 320, Campinas - SP, Brazil

## Abstract

**Background:**

From shotgun libraries used for the genomic sequencing of the phytopathogenic bacterium *Xanthomonas axonopodis *pv. *citri *(XAC), clones that were representative of the largest possible number of coding sequences (CDSs) were selected to create a DNA microarray platform on glass slides (*XACarray*). The creation of the *XACarray *allowed for the establishment of a tool that is capable of providing data for the analysis of global genome expression in this organism.

**Findings:**

The inserts from the selected clones were amplified by PCR with the universal oligonucleotide primers M13R and M13F. The obtained products were purified and fixed in duplicate on glass slides specific for use in DNA microarrays. The number of spots on the microarray totaled 6,144 and included 768 positive controls and 624 negative controls per slide. Validation of the platform was performed through hybridization of total DNA probes from XAC labeled with different fluorophores, Cy3 and Cy5. In this validation assay, 86% of all PCR products fixed on the glass slides were confirmed to present a hybridization signal greater than twice the standard deviation of the deviation of the global median signal-to-noise ration.

**Conclusions:**

Our validation of the *XACArray *platform using DNA-DNA hybridization revealed that it can be used to evaluate the expression of 2,365 individual CDSs from all major functional categories, which corresponds to 52.7% of the annotated CDSs of the XAC genome. As a proof of concept, we used this platform in a previously work to verify the absence of genomic regions that could not be detected by sequencing in related strains of *Xanthomonas*.

## Findings

Citrus canker, or cancrosis, is a disease that affects most species of the *Citrus *genus [1]. Its symptoms can be observed on leaves, fruits or branches and are characterized by small, pointed and spongy pustules that are surrounded by a chlorotic halo on both sides of the leaf. This halo tends to spread quickly through the tissue, increasing its diameter and becoming brownish or darker in color with a rough and salient appearance [2]. The causal agent of cancrosis is the bacterium *Xanthomonas axonopodis *pv. *citri *(XAC) [3], more recently renamed as *Xanthomonas citri *subsp. *citri *[4,5] whose genome was completely sequenced in 2002 [6] and compared with others organisms [6-8]. Data from this sequencing project revealed that the XAC genome contains a circular chromosome of 5.2 Mbp and two plasmids (33 and 64 Kbp), containing a total of 4,489 coding regions [6].

To perform the complete sequencing of this phytopathogen, shotgun libraries containing 46,462 clones were created and distributed over approximately 500 96-well plates, representing 98% of the genome, with an estimated coverage of seven times its size [6]. The remaining 2% of the genome was sequenced using a cosmid library.

Upon completion of the sequencing project, the clones were stored with the expectation that they would be useful for future functional genomics studies. We here explore one of the possible applications of this library, namely the use of these clones to build a DNA microarray platform for use in studies of gene expression and genotyping [9]. Building on the data provided by the XAC genome project, this tool will enable the exploration of the physiological and biochemical machinery of this organism in distinct environmental situations, both *in vitro *and *in vivo*, thus providing clues to the working of *Xanthomonas *metabolism and the mechanisms of infection and disease.

### Clone selection from shotgun libraries

**Figure 1:**
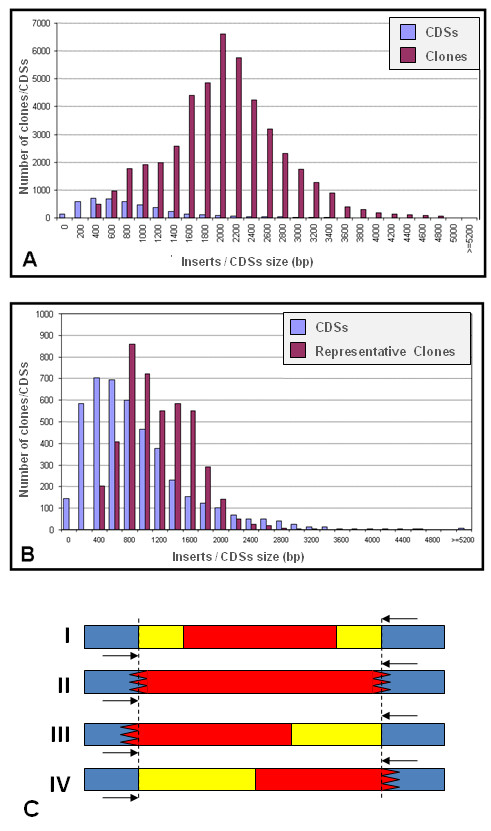
**(A) Size distribution of the 46,462 inserts generated by the shotgun technique and of the 4,489 CDSs annotated in the XAC genome. (B) Distribution of the 4,421 selected clones, which best represented 3,084 CDSs of the XAC genome**. Note in both figures that for the CDSs that were 0 to 200 bp in length and for a small fraction of genes that were up to 400 bp in length, representative clones were not found in the shotgun library, following the selection criteria described in the methods. **(C) Representative scheme of the type of clones selected from the XAC genomic library**. The blue region represents the sequence of the pUC19 cloning vector. The region marked by dotted vertical lines bounds the insert of the clone. The region where the CDSs do not overlap is marked in yellow, and the corresponding overlap is marked in red. The region with a serrated end represents the rupture of the sequence of the gene of interest. Black arrows represent the universal oligonucleotide primer (M13) used for the PCR amplification of the insert.

The clones in the XAC sequencing libraries were generated from total bacterial DNA (chromosomal DNA + plasmid DNA) using shotgun methods [10] and cloning into the pUC18 vector. The number of clones totaled 46,462 with inserts ranging in size from 400 to 5,200 bp (Figure 1AB) [6]. All of these clones were placed in 496 96-well plates, and stored at -80°C. In order to represent the CDSs in the *XACarray*, clones that included the largest fragments (0.4 to 3 Kbp) of each CDSs of interest were selected. However, not all of the CDSs were represented by clones that included a single open-reading frame (ORF). Actually, many CDSs were represented by clones that possessed inserts with parts of other CDSs or even whole CDSs. Thus, the selected clones were divided into four possible overlap types, considering the sequence segment of interest in relation to the insert as a whole: I) the CDS is completely represented within the insert and is surrounded on both sides by intergenic regions and/or other CDSs; II) the insert is an internal fragment of the corresponding CDS; III) part of the interested CDS flanking the left margin of the insert, or IV) part of the interested CDS flanking the right margin of the insert (Figure 1C). Details of the relationship between the insert and CDS of interest are described in the results and are summarized in Table 1.

**Table 1:** Analysis of clone selection grouped according to the overlap models.

Type of clones	Total of selected clones	Average size of overlap Insert/CDS	Average size of non-overlap Insert/CDS
I	2055	635	835
II	463	1121	0
III	827	822	418
IV	1124	726	481
**Total**	**4421**	**830.5**^**a**^	**578**^**b**^

Note that the average total of the CDS/insert overlap (^a^) is greater than the average of the non-overlap (^b^).

### Amplification, purification and identification of PCR products

The selected clone inserts were amplified through PCR after DNA extraction by the boiling preparation technique [11]. The amplification reactions were performed with an initial denaturation at 95°C for 5 minutes, followed by 40 denaturing cycles at 95°C for 45 seconds, annealing at 50°C for 30 seconds and extension at 72°C for 1 minute in a final volume of 50 μL of amplification solution containing 1.0 μL boiling prep product, 1 U *Taq *DNA Polymerase™ (Invitrogen), 40 mM dNTPs (Invitrogen) and 10 pmols of each universal oligonucleotide M13 (forward 5'-GTTTTCCCAGTCACGAC-3' and reverse 5'-CAGGAAACAGCTATGAC-3'). In an attempt to increase the amount of the generated product, 1.0 μL of the amplification reaction was added to new amplification solution with the same volume and concentration of reagents and submitted to the amplification protocol described above.

The amplification products were purified with Multiscreen Millipore™ (cat. # MAFB NOB 50) filters plates and diluted in 50 μL of 10 mM Tris pH 8.0 directly into 96-well plates. The purification products were then manually combined in 8 384-well plates; one of these plates contained the hybridization controls (ScoreCard [12]).

**Figure 2:**
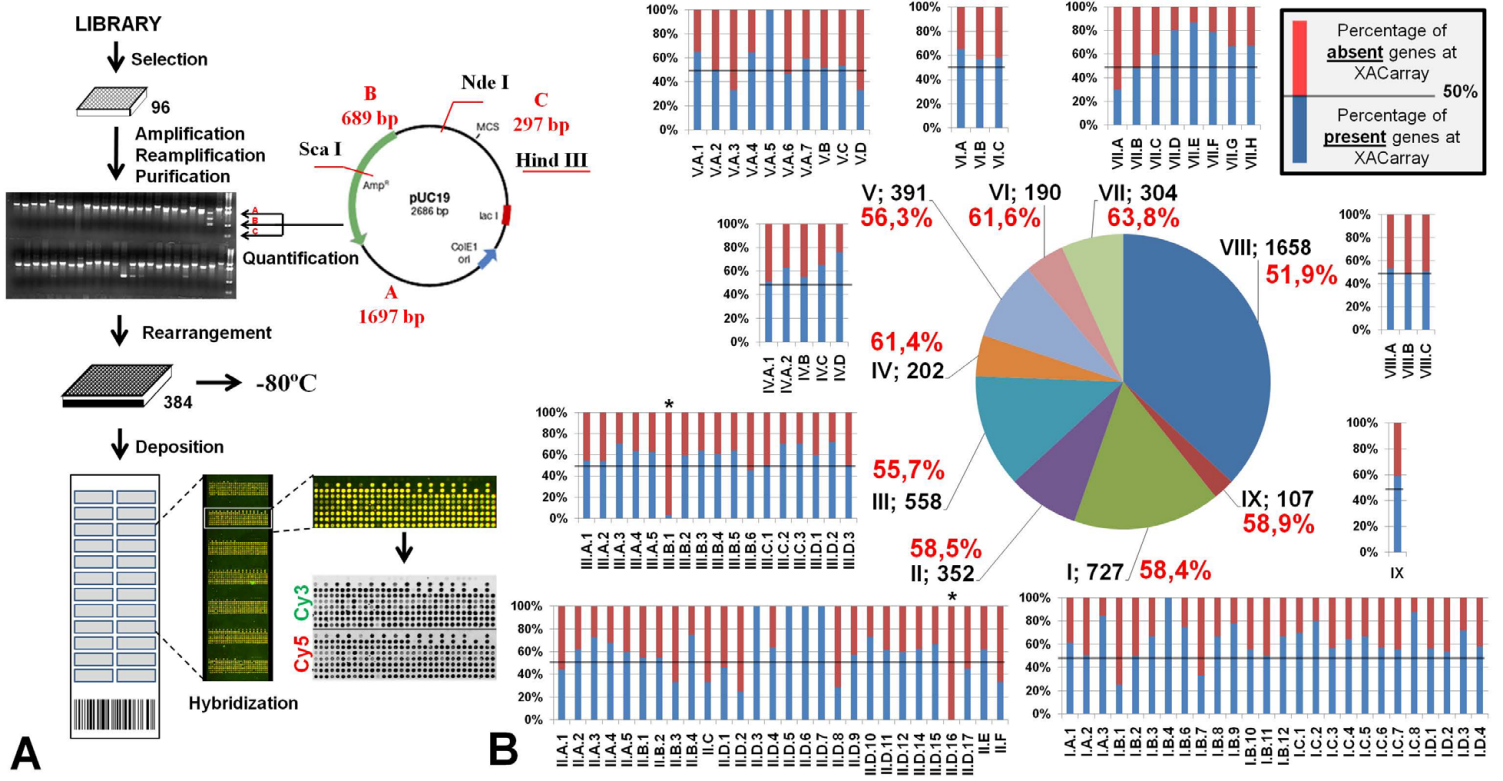
**(A) Figurative fluxogram of the *XACarray *construction protocol**. The clones were selected from the library, amplified and re-amplified. The PCR products were purified and visually analyzed for concentration based on a molecular weight standard created specifically for this purpose and by re-sequencing of one of the ends of the insert. The samples were rearranged in 384 well plates and fixed onto the slides. The image demonstrates the result obtained with the hybridization of the DNA samples labeled with the fluorophores Cy3 and Cy5, highlighting subarray 7, for which the monochromatic analysis data demonstrates the quality of the hybridization. **(B) Statistical distribution of the products fixed on the *XACarray *based on primary, secondary and tertiary annotation categories **[6]. Note that for only two subcategories (III.B.1 and II.D.16), no products were represented on the *XACarray*, and in most of the remaining categories, the total of the genes present (blue bars) exceeds the absent genes (red bar).

In all steps, the PCR products were evaluated by electrophoresis in 1% agarose gels stained with ethidium bromide [13]. Amplicon size and quantity were estimated by comparison to a molecular weight standard that was created specifically for this purpose (Figure 2A). This standard contained three DNA fragments of 1,697, 689 and 297 bp, respectively, with pre-determined concentrations. The standard was produced by cleavage of the pUC19 plasmid with the restriction enzymes *Hind*III, *Nde*I and *Sca*I, respectively, and was purified using a cesium chloride density gradient column [14]. This strategy allowed for comparative visual analysis of the sample concentrations in the gels, eliminating the need for individual quantification of each observed product.

To ensure that each amplified PCR product was in fact the clone selected for each CDS, an end of the insert in each of the clones was sequenced using the universal oligonucleotide primer M13R and the BigDye terminator v3™ kit (Applied Biosystems). These sequences were then compared to the database of sequencing reads and the XAC genome using the BLASTn algorithm [15].

### Fixation of PCR products onto glass slides

Equal volumes of DMSO were added to the purified PCR products in 384-well plates so that the final concentration was approximately 200 to 400 fmol/μL of DNA [16]. Afterwards, DNA was deposited onto Type 7 mirrored glass slides (Amersham Biosciences/GE Healthcare) using a robot (*Generation *III *Microarrays Spotter*™ - Amersham Biosciences/GE Healthcare). This robot allowed for the deposition of up to 4,608 DNA samples that were organized into 12 subsets (*subarrays*) of 384 spots (12 × 32) (Figure 2A). The set of 3,072 probes, which corresponded to the products on the 384-well plates and included positive and negative controls, was placed in duplicate in the two longitudinal halves of the slide. The duplicates were called set A and set B, respectively, and constituted a technical replica on each slide (Figure 2A). After DNA deposition, the slides were submitted to 50 mJ UV light, which promoted the fixation of the DNA onto the support. The slides were stored in desiccators (relative moisture ~5%), ready to be used for any experimental purpose.

#### XAC cultivation and DNA extraction

The XAC 306 isolate was extracted from *Citrus limonia L*. Osbeck (rangpur lime)-infected leaves and stored in 30% glycerol at room temperature until use. The strain was sowed to reactivate its growth in NA solid medium (3 g/L of meat extract, 5/L g of peptone, 5 g/L of sodium chloride, 15 g/L of agar) and was incubated at 28°C for 48 hours. Samples of the reactivated strain were minced in 12 mL of NA liquid medium in 25-mL Falcon tubes and maintained under gentle agitation at 28°C for 48 hours. Appropriated antibiotic was used when necessary. After this period, the samples were centrifuged for 30 minutes at 3,000 *g *at room temperature. The supernatant was discarded, and 12.5 mL of wash buffer (10 mM Tris-HCl, pH 8.8, 3.0 mM KCl, 1.25 mM NaCl) was added to the bacterial pellet. The samples were shaken vigorously using a vortex and then centrifuged for 15 minutes at 3,000 *g *at room temperature. The bacterial pellet was resuspended again, now using 10 mL of D buffer (25 mM sodium citrate, pH 7.0, 5.0 g/L sarkosyl, 4 M guanidine isothiocyanate), and incubated in a 65°C water bath for 1 hour to promote cell lysis. After the lysis step, 5.0 mL of P buffer (667 mM Tris-HCl, 833 mM NaCl, 83 mM EDTA, pH 8.0) was added. The solution was shaken by inversion and centrifuged for 30 minutes at 3,000 *g *at room temperature. Aliquots (1.0 mL) of the supernatant were transferred to tubes with a 2.0 mL capacity, 1.0 mL of isopropanol was added and the samples were kept at -20°C. The samples were then homogenized by inversion and centrifuged for 45 minutes at 3,000 g at 4°C. The DNA pellet was resuspended, washed twice with 70% ethanol to promote precipitation and centrifuged for 15 minutes at 3,000 *g*. Finally, the DNA pellet was resuspended in 200 μL TE buffer (10 mM Tris-HCl, pH 8.0, 0.1 mM EDTA) containing 1 μg/mL of RNaseA and incubated for 1 hour at 37°C. The quality and concentration of the extracted DNA was evaluated on a 0.8% agarose gel stained with ethidium bromide [14]; the profile of the band obtained for the DNA samples was compared to a molecular weight standard with known sizes and concentrations (Figure 2A).

### Preparation of labeled DNA and microarray hybridization

Double-stranded DNA labeled with fluorophore was produced by Cy3-dCTP or Cy5-dCTP™ (Amersham Biosciences/GE Healthcare) incorporation during polymerization from 2 μg XAC total DNA (chromosomal + plasmid) that had been fragmented through the shearing technique (~14 times through a syringe with 8 cm and 18 gauge needles) [17]. The reaction was performed using 1 μL of the DNA polymerase Klenow Fragment™ (Gibco) at a high concentration (40 U/μL) and 500 μg of random nonamer primers [18]. Afterwards, the labeled DNA was purified with Multiscreen™ filter plates (Millipore), and the total amount of incorporated fluorophore was indirectly quantified through absorbance detection at 550 *nm *for Cy3 and 650 *nm *for Cy5. To ensure accuracy in the analysis, equivalent quantities of fluorescent DNA were used in the microarray hybridizations, which were performed for 16 hours at 42°C in hybridization buffer (Amersham Biosciences/GE Healthcare) with 50% formamide. According to protocols described previously by Koide and coauthors [18], after washing, the slides were dried by applying a jet of compressed nitrogen gas. Microarray data was aquired using a *Generation III Scanner™ *(Amersham Biosciences/GE Healthcare) in order to obtain images of each fluorescent channel, Cy3 and Cy5, for each of the microarray probes (Figure 2A).

### Detection, quantification and normalization of fluorescence signals

After the microarrays were scanned, the crude signal intensity of the images was determined using Array Vision 6.0™ software (Image Research/Molecular Dynamics). For each spot on the microarray, which represents a specific probe, the foreground and background intensity were measured.

Data preparation included subtraction of the local noise for each spot, and calculation of average fluorescece density and artifacts removal (MTM Dens). Array Vision software allowed the exclusion of bad pixels (pixels within a section that showed signal intensity either above or under four median absolute deviations (MADs) of the signal intensity of all of the pixels in the section).

## Results

### Construction of XAC DNA microarrays (*XACarray*)

The selection of clones from the XAC shotgun libraries allowed for the identification of 4,421 clones, which represented 3,084 individual CDSs of the XAC genome (Figure 1A). The remaining 1,405 CDSs, which would complete the genome, were not selected because they represented very small CDSs, smaller than 200 bp for example (Figure 1B), or because they were not physically found in the shotgun library [6].

Table 1 and Figure 1C present the distribution and profile, respectively, of numbers of CDSs and selected clones, as well a schematic model of the CDS/insert overlap, which was used as a reference in the classification of the clones. It is worth noting that the ideal clones are classified as overlap type II, because the insert of these clones as a whole represents a part of the CDS of interest. Therefore, overlap of other genes or intergenic regions do not exist. However, this type of clone was the least representative in the *XACarray *and consisted of only 292 (11.1%) of the fixed CDSs (Table 2). For the other three types of selected clones (I, II and IV), an overlap region of other CDSs or intergenic regions with the insert was always present. Of those, the most representative overlap type was type I (see methods), totaling 1,184 fixed CDSs on the slide (44.9%) and presenting a insert/CDS of interest ratio of 0.76 (Table 2).

**Table 2:** Analysis of the CDSs deposited on the *XACarray *based on the hybridization signal during the experimental validation of the platform.

Type of clones	Physical analysis (Fixed PCR products)			**Annotation**^**c**^		Hybridization signal	
	
	Clones n° (%)	AIS^a^ (bp)	C/I^b^ (%)	Function n° (%)	Hypothetical n° (%)	Function n° (%)	Hypothetical n° (%)
I	1,184 (57.6)	1,487	56.8	631 (53.2)	553 (46.8)	574 (90.9)	485 (87.7)
II	292 (63.1)	1,121	100	235 (80.5)	57 (19.5)	215 (91.5)	52 (91.2)
III	490 (59.3)	1,211	69.9	343 (70.0)	147 (30.0)	323 (94.2)	123 (83.7)
IV	673 (59.9)	1,209	70.5	466 (69.2)	207 (30.8)	436 (93.6)	184 (88.9)
**Total**	**2,639 (59.7)**	**1,324**	**82.1**	**1,675 (63.5)**	**964 (36.5)**	**1,521 (90.8)**	**844 (87.6)**

						**2,365 (89.6)**	

^a ^AIS (average of insert size).

^b ^C/I (average of overlapping size CDS/Insert)

^c ^Annotation based on Silva *et al *2002 [6].

Note that the number of clones that were amplified and positively annotated represent only 59.7% of the total clones initially selected (4,421), and that 2,365 of individual CDSs presented a positive hybridization signal, which represent 52.7% of the total annotated CDSs in the genome and 89.6% of the fixed products.

After the *in silico *selection, the clones were rearranged in 47, 96 well plates using a Q-Bot robot™ (Genetix, UK), and the inserts were amplified by PCR (see methods). Approximately 5% of the clones were shown to be not viable, and 10% presented an insert with a size different from that which was predicted (data not shown), suggesting the existence of probable errors in clone labeling, a common occurrence in clone library construction, even in commercial libraries [19]. Moreover, another 15% presented negative results with respect to their amplification. For these reasons, it was necessary to re-sequence one end of the insert of each of the previously selected clones in order to confirm the identity of all of the clones. The sequences were compared to the XAC genome, and the identity of the clones was corrected when necessary. Unfortunately, at the end of all the steps described above, approximately 30% of the initially selected clones (1,326) could not be used. Thus, at the end of these steps, expected identity of 2,653 clones were confirmed and this set was used. They were rearranged again, now in 28 96-well plates. Although the number of clones was severely reduced, the microarray contained a set of clones with a reliable identity. However, after the PCR was performed to increase the amount of the probe to be immobilized on the slides, the number of clones was reduced to 2,639, which represented 58.8% of the XAC genome (Table 2). To this set of 2,639 PCR products, 121 products derived from amplifications of genomic DNA sequences using specific oligonucleotides that were previously acquired for other experimental proposals were added; these products represented the sequences of genes of particular interest, including the genes *vir*, *hrp*, *rpf *and *gum*. Thus, the 2,760 PCR products were purified (probes), rearranged in 8 384-well plates, and used in the construction of microarrays as described above.

It is necessary to highlight that the molecular mass pattern obtained from the pUC19 plasmid not only facilitated the analysis of the amplified product, but also demonstrated that this approach was correct. The banding pattern quantification of some randomly selected PCR products corresponded to the visual quantifications that were performed using only the agarose gels (data not shown).

### *XACarray *validation

For an initial validation of the quality of the *XACarray*, hybridization with XAC genomic DNA labeled with distinct fluorophores was performed. Figure 2A shows the fluxogram of the *XACarray *protocol and the hybridization images obtained using this probe. Note that the spots on the slide are practically all yellow in color, which visually represents the hybridization of probes labeled with Cy3 and Cy5 in similar quantities. This result can be observed more rigorously from the monochromatic images obtained from laser scanning at 550 and 650 nm, respectively.

The analysis of these images revealed a noticeable signal above the background noise for 88.5% of the CDSs represented in the *XACarray*, a percentage that is equivalent to 2,365 individual CDSs of the XAC genome (52.7%). Table 2 summarizes the physical features of the 2,670 probes deposited on the *XACarray*. The probes are represented in Figure 1C in relation to the type of overlap of the insert and the average size of these sequences. The classification of the CDSs regarding their annotation characteristics is shown in Table 2. The CDS are classified into two major groups: CDSs previously annotated with a determined putative function, which represent 63.5% of the *XACarray*; and CDSs that compose the hypothetical group, in this case exclusive hypothetical and conserved hypothetical in other organisms, which represent the other 36.5% of the composition of the array.

**Figure 3:**
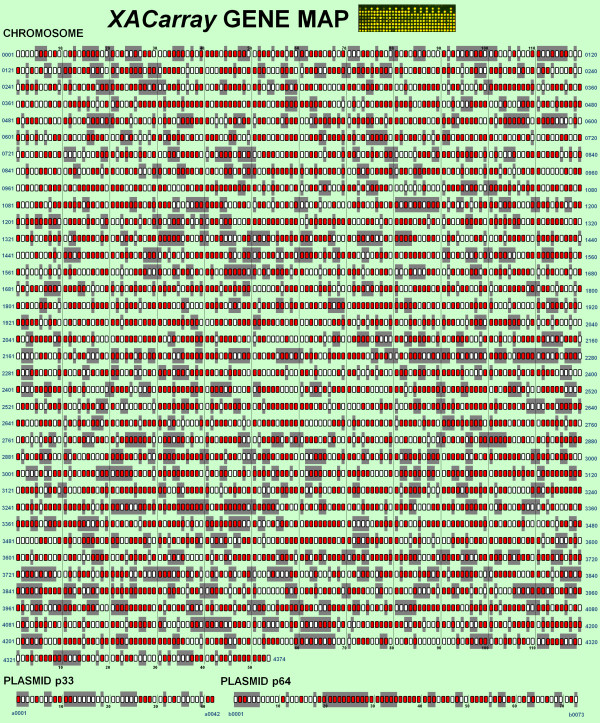
**XAC genes presented on the *XACarray***. Those CDs whose products were fixed on the *XACarray *are marked in red (2,760 in total). The gray background highlights the genes annotated as hypothetical or hypothetical conserved. The CDS products highlighted in the figure are presented in the additional file 1.

Quantitatively, the *XACarray *can be considered to be representative of the XAC genome, not only for containing probes that represent more than 50% of all XAC genes, but also for presenting 83.4% of all of its annotation categories, with more than 50% of the functionally annotated genes (Figure 2B). This global vision of representation can be observed in detail in Figure 3 and additional file 1, which correlates the gene map of annotations to the CDSs fixed on the slide.

## Conclusions

In this study, we developed a new and low-cost method to generate a DNA microarray. Previously, Astua-Monge et al. [20] used a DNA macroarray, implemented on nylon membranes and containing probes for 279 XAC genes associated with pathogenicity and virulence, to investigate genes differentially expressed in a synthetic medium (XVM2) that simulates *in planta *conditions. In contrast, our plataform is the first DNA microarray platform for XAC that representes more than 50% of all XAC genome CDSs.

The platform was constructed from shotgun libraries, previously generated for the genomic sequencing project. Although the clones of type II are ideal, because they are restricted to include just an internal fragment of the target gene, we have made an effort to select clones that would not overlap with other genes. In our array, there are at least 1,290 clones (~30% of the genome, not counting the ones for which specific primers were designed) that contain genome fragments that do not overlap any gene besides the target gene they were selected to represent. Also, many genes that have fragments in the same clone will correspond to operons and could, in a first crude analysis, be regarded as a single expression unit, with additional experiments clarifying which gene(s) are indeed relevant. Therefore, our platform can be used to generate lists of putative differentially expressed genes in XAC under different physiological conditions.

On the other hand, the presence of overlapping probes makes it necessary to subsequently validate individual candidate genes using methods with higher specificity and sensitivity, like qRT-PCR. Moreover, under most conditions, some more expensive methods, like designed-probe chips and RNA-Seq will always provide better specificity/sensitivity rates and better reproducibility and such methods should be preferred whenever researchers have access to them. Functional results using this platform were described in a recent work that use this tool to validated putative insertion/deletion regions between different and incomplete genomes sequences of other *Xanthomonas *species [21].

## Competing interests

The authors declare that they have no competing interests.

## Authors' contributions

**LMM**: Responsible for the amplification, purification and fixation of the material onto the slides, participated in the validation of the hybridization platform, preparation of the manuscript and elaboration of all figures; **MLL**: Participated in the amplification reactions and in the preparation of the manuscript; **RFS**: Responsible for assembling the software for *in silico *selection of the representative clones and participated in the preparation of the manuscript; **PZ**: Participated in the preparation of the manuscript; **ACRS**: Participated in the preparation of the proposal, the construction of the shotgun libraries and the assembly of the platform itself; **AMS**: Participated in the project design and the data analysis; **JAF**: Participated in the construction of the shotgun libraries, the data analysis and the preparation of the manuscript. All authors read and approved the final manuscript.

## Supplementary Material

Additional file 1 **Additional informations about the CDSs highlighted in Figure **3. Click here for file
