# Supplementary material for: Development and validation of a Xanthomonas axonopodis pv. citri DNA microarray platform (XACarray) generated from the shotgun libraries previously used in the sequencing of this bacterial genome
Source: BMC Res Notes. 2010 May 27;3:150. doi: 10.1186/1756-0500-3-150 (PMC2890508; doi:10.1186/1756-0500-3-150)
Supplement: Additional file 1 — Additional informations about the CDSs highlighted in Figure 3. [file 1756-0500-3-150-S1.PDF]

Gene_ID	Clone_ID	Category	Product
**XAC0006**	A0QR9860E10	VIII.A	conserved hypothetical protein
**XAC0007**	A0QR6702C11	VIII.A	conserved hypothetical protein
**XAC0009**	A0QH6327F01	VII.C	biopolymer transport ExbB protein
**XAC0012**	A0UV6456E10	II.D.6	pyridoxal phosphate biosynthetic protein
**XAC0013**	A0CE6504G10	VIII.A	conserved hypothetical protein
**XAC0015**	A0QH6732D11	VIII.B	hypothetical protein
**XAC0016**	A0JE1145F05	VIII.B	hypothetical protein
**XAC0017**	A0QR6839E01	VIII.A	conserved hypothetical protein
**XAC0018**	A0UV6711D01	VIII.C	Xanthomonas conserved hypothetical protein
**XAC0021**	A0CE6830F01	VIII.A	conserved hypothetical protein
**XAC0023**	A0UV6843F05	III.C.3	carboxyl-terminal protease
**XAC0026**	A0RN1447G12	VIII.A	conserved hypothetical protein
**XAC0028**	A0CE6314A10	VII.D	cellulase
**XAC0029**	A0UV6709A09	VII.D	cellulase
**XAC0033**	A0QR5903A03	II.A.1	glutamate synthase, alpha subunit
**XAC0034**	A0CE9723B02	VIII.A	conserved hypothetical protein
**XAC0035**	A0UE9889F07	VIII.A	conserved hypothetical protein
**XAC0036**	A0QR6804H02	VIII.C	Xanthomonas conserved hypothetical protein
**XAC0038**	A0QR9851A02	VIII.A	conserved hypothetical protein
**XAC0040**	A0QR6730C07	VIII.B	hypothetical protein
**XAC0041**	A0UV6707D05	IV.C	mannosyltransferase B
**XAC0042**	A0QR5902D11	IV.C	glycosyltransferase
**XAC0043**	A0JJ1443G01	IV.C	UDP-glucose lipid carrier transferase
**XAC0045**	A0QH9770D03	VIII.B	hypothetical protein
**XAC0046**	A0UV9820D11	IV.C	UDP-N-acetyl-D-mannosamine transferase
**XAC0047**	A0UT9849A02	IV.C	galactosyltransferase
**XAC0048**	A0UV9901E10	VIII.B	hypothetical protein
**XAC0049**	A0EC6322A05	VIII.B	hypothetical protein
**XAC0052**	A0UV6511H02	VIII.B	hypothetical protein
**XAC0053**	A0UE6759D05	IX	methyltransferase
**XAC0054**	A0AC6824C06	I.B.11	UDP-glucose epimerase (degenerated)
**XAC0056**	A0JJ0709F09	VII.E	polysaccharide export protein
**XAC0057**	A0CE6829A09	V.A.7	transport protein
**XAC0059**	A0UV1651F08	II.A.2	asparagine synthetase like protein
**XAC0061**	A0UV6845G12	VIII.A	conserved hypothetical protein
**XAC0063**	A0QR6614C06	II.D.17	aryl sulfotransferase
**XAC0064**	A0QH6813G09	IX	acetyltransferase
**XAC0065**	A0QR6840H03	IX	microcystin dependent protein
**XAC0067**	A0CE6743E05	IX	microcystin dependent protein
**XAC0070**	A0UT6815D11	IV.A.1	ankyrin-like protein
**XAC0074**	A0EC6474F02	V.A.7	TonB-dependent receptor
**XAC0075**	A0JJ0702C08	I.D.2	xylose repressor-like protein
**XAC0077**	A0JE5637E10	VIII.A	conserved hypothetical protein
**XAC0078**	A0JE1637C05	III.B.5	ATP-dependent RNA helicase
**XAC0081**	A0AC6823A06	VIII.A	conserved hypothetical protein
**XAC0083**	A0EC0204C09	I.C.3	short chain dehydrogenase
**XAC0085**	A0UV6818F09	VIII.A	conserved hypothetical protein
**XAC0087**	A0QR9705E01	VIII.B	hypothetical protein
**XAC0091**	A0JJ1371H02	VI.C	ISxac3 transposase
**XAC0093**	A0CE6480F05	VI.C	ISxac1 transposase
**XAC0096**	A0JJ1546D10	VIII.C	Xanthomonas conserved hypothetical protein
**XAC0098**	A0UV6821H09	VIII.B	hypothetical protein
**XAC0099**	A0QR5904C02	VIII.B	hypothetical protein

**XAC0102**	A0RN1406D06	VIII.A	conserved hypothetical protein
**XAC0106**	A0QR5327B01	III.A.4	MutT/nudix family protein
**XAC0110**	A0UT9877B11	V.A.1	proline/betaine transporter
**XAC0111**	A0JE1144B02	VIII.C	Xanthomonas conserved hypotheticalprotein
**XAC0112**	A0EC6476C04	VIII.A	conserved hypothetical protein
**XAC0114**	A0UV5526B08	VIII.A	conserved hypothetical protein
**XAC0116**	A0QR6613D02	VIII.A	conserved hypothetical protein
**XAC0117**	A0QR5206A01	VIII.A	conserved hypothetical protein
**XAC0121**	A0CE5319H08	III.C.3	TldD protein
**XAC0124**	A0AC6825D11	I.B.3	fructose-1,6-bisphosphatase
**XAC0126**	A0UV6763B05	V.A.7	iron transporter
**XAC0131**	A0QR9758E02	VIII.C	Xanthomonas conserved hypothetical protein
**XAC0132**	A0QR9705D11	VIII.A	conserved hypothetical protein
**XAC0133**	A0UV6846B06	I.C.2	L-lactate dehydrogenase
**XAC0135**	A0AC6825A10	I.D.1	two-component system, sensor protein
**XAC0136**	A0QR6730E03	I.D.1	two-component system, regulatory protein
**XAC0138**	A0UV6821C02	VIII.B	hypothetical protein
**XAC0141**	A0AC6827F08	VIII.A	conserved hypothetical protein
**XAC0143**	A0QH6713C11	VII.D	2-keto-3-deoxygluconate kinase
**XAC0144**	A0UV5502G02	V.A.7	TonB-dependent receptor
**XAC0145**	A0UV6736H12	VIII.A	conserved hypothetical protein
**XAC0146**	A0QR6840H02	VIII.B	hypothetical protein
**XAC0147**	A0QR6705H01	VIII.A	conserved hypothetical protein
**XAC0149**	A0UV6711E03	VIII.B	hypothetical protein
**XAC0150**	A0QR6801A02	VIII.B	hypothetical protein
**XAC0152**	A0EC6324C02	VI.C	ISxac3 transposase
**XAC0153**	A0QH6326F12	VI.C	ISxac3 transposase
**XAC0154**	A0JE1034E08	I.A.1	alpha-amylase
**XAC0155**	A0JJ1003E03	III.D.1	trehalose synthase
**XAC0160**	A0UV6708A05	I.A.1	xylanase
**XAC0164**	A0CE6831F11	V.A.7	C4-dicarboxylate transport protein
**XAC0165**	A0UT6817H07	I.A.1	xylosidase/arabinosidase
**XAC0166**	A0UV6818E01	I.D.2	transcriptional regulator lacI family
**XAC0167**	A0QR6728D09	VIII.B	hypothetical protein
**XAC0168**	A0UV6709D06	VII.D	5-keto-4-deoxyuronate isomerase
**XAC0170**	A0UT6815F11	IX	sugar-phosphate isomerase
**XAC0171**	A0UE6759H05	I.A.1	rhamnogalacturonan acetylesterase
**XAC0172**	A0QR9756G08	VIII.A	conserved hypothetical protein
**XAC0173**	A0UT6386G04	VIII.C	Xanthomonas conserved hypothetical protein
**XAC0177**	A0AC0115D10	VIII.A	conserved hypothetical protein
**XAC0178**	A0CE6742A12	VIII.A	conserved hypothetical protein
**XAC0179**	A0QR6725B12	V.A.7	ABC transporter ATP-binding protein
**XAC0182**	A0UV6762A12	V.A.7	ABC transporter ATP-binding protein
**XAC0183**	A0AC6824C07	V.A.1	ABC transporter amino acid permease
**XAC0184**	A0QR6840A01	VIII.A	conserved hypothetical protein
**XAC0187**	A0UV6710C02	IV.B	HipA protein
**XAC0188**	A0QR5902G11	I.D.2	transcriptional regulator
**XAC0189**	A0QR6729E09	I.C.3	indolepyruvate ferredoxin oxidoreductase chain alpha
**XAC0190**	A0QR9776E12	VIII.C	Xanthomonas conserved hypothetical protein
**XAC0192**	A0QR5321E04	V.B	partition protein
**XAC0193**	A0QR5702C06	VIII.A	conserved hypothetical protein
**XAC0194**	A0QR5701E10	VIII.A	conserved hypothetical protein
**XAC0195**	A0CE5318C03	II.E	cardiolipin synthase
**XAC0196**	A0QR5703C03	VIII.A	conserved hypothetical protein

**XAC0197**	A0QR6839C05	IV.C	acetyltransferase
**XAC0198**	A0UE6399D11	VIII.A	conserved hypothetical protein
**XAC0199**	A0UV6709F06	VIII.A	conserved hypothetical protein
**XAC0201**	A0AC6408F07	I.C.2	alcohol dehydrogenase
**XAC0202**	A0AC0113A10	VIII.C	Xanthomonas conserved hypothetical
**XAC0205**	A0JE1331G02	I.D.2	nitrogen regulatory protein P-II
**XAC0206**	A0QR6002D03	V.A.4	ammonium transporter
**XAC0208**	A0UV6709D04	I.D.1	two-component system, regulatory protein
**XAC0210**	A0QR6705H04	VII.C	superoxide dismutase
**XAC0211**	A0UV6763H05	I.B.10	lactoylglutathione lyase
**XAC0212**	A0UT9703B08	VIII.A	conserved hypothetical protein
**XAC0213**	A0UV5523B02	I.A.3	acyl-CoA thiolase
**XAC0217**	A0UV6764C10	IV.C	glycosyltransferase
**XAC0218**	A0CE6830B11	VIII.A	conserved hypothetical protein
**XAC0221**	A0AC9761F03	III.C.2	protein-export protein
**XAC0222**	A0UV5502C01	III.D.2	glycerol-3-phosphate dehydrogenase
**XAC0223**	A0JJ1371F07	VIII.A	conserved hypothetical protein
**XAC0225**	A0UV9820H12	I.D.1	two-component system, sensor protein
**XAC0226**	A0QH6813C04	I.D.1	two-component system, regulatory protein
**XAC0228**	A0CE6830H03	VIII.B	hypothetical protein
**XAC0230**	A0QR6701H05	III.B.4	tRNA/rRNA methyltransferase
**XAC0232**	A0QR6614F03	VIII.A	conserved hypothetical protein
**XAC0233**	A0UV6708E11	II.E	3-oxoacyl-[ACP] synthase III
**XAC0238**	A0UV6763G05	II.E	NAD(P)H steroid dehydrogenase
**XAC0242**	A0QR6752A12	II.D.11	ubiquinone biosynthesis protein
**XAC0243**	A0CE9839A10	VIII.A	conserved hypothetical protein
**XAC0244**	A0QR6702G10	III.B.5	pseudouridylate synthase
**XAC0245**	A0QR5407B10	VIII.A	conserved hypothetical protein
**XAC0248**	A0RN1630E07	II.A.2	asparaginase
**XAC0249**	A0RN1630A06	III.C.3	peptidyl-dipeptidase
**XAC0251**	A0QR6730C11	I.D.2	transcriptional regulator tetR family
**XAC0252**	A0UV9883B05	VIII.A	conserved hypothetical protein
**XAC0254**	A0UT6815A01	V.A.4	Na+/H+-exchanging protein
**XAC0256**	A0UV6818D12	I.B.4	malate synthase
**XAC0257**	A0UV6762F09	I.B.4	isocitrate lyase
**XAC0259**	A0CE6743A02	VIII.A	conserved hypothetical protein
**XAC0260**	A0AC6825E09	VIII.C	Xanthomonas conserved hypothetical protein
**XAC0261**	A0UE6399D10	VIII.A	conserved hypothetical protein
**XAC0262**	A0CE6507H12	III.C.3	dipeptidyl anminopeptidase
**XAC0263**	A0EC6321D09	II.E	biotin carboxylase
**XAC0265**	A0UV1654C03	I.A.3	acyl-CoA dehydrogenase
**XAC0266**	A0QR5408D08	I.D.2	transcriptional regulator acrR family
**XAC0267**	A0QR6375D12	VIII.A	conserved hypothetical protein
**XAC0268**	A0QR6375E10	VIII.A	conserved hypothetical protein
**XAC0270**	A0CE9724C12	VIII.A	conserved hypothetical protein
**XAC0273**	A0UV6764D05	I.C.3	cytochrome C5
**XAC0274**	A0QH6215E06	III.A.4	nuclease
**XAC0275**	A0UT9846C06	VIII.C	Xanthomonas conserved hypothetical protein
**XAC0279**	genomic_DNA	VIII.C	Xanthomonas conserved hypothetical protein
**XAC0280**	A0UV6210E02	IX	ATPase
**XAC0283**	A0UE9886C11	IX	hydrolase
**XAC0285**	A0UV9883F02	VIII.C	Xanthomonas conserved hypothetical protein
**XAC0286**	A0JJ1085D10	VII.A	avirulence protein
**XAC0287**	A0QR6481F04	I.C.3	quinone oxidoreductase

**XAC0288**	A0AC1317G08	I.C.3	oxidoreductase
**XAC0289**	A0CE6743F09	VIII.A	conserved hypothetical protein
**XAC0291**	A0QR6367D10	IV.A.2	Oar protein
**XAC0292**	A0CE6831A04	VIII.A	conserved hypothetical protein
**XAC0297**	A0UV6455E01	VIII.A	conserved hypothetical protein
**XAC0298**	A0QR5518B07	VIII.A	conserved hypothetical protein
**XAC0300**	A0QR5408C07	II.A.2	serine-pyruvate aminotransferase
**XAC0302**	A0UV6711D12	I.D.2	transcriptional regulator lysR family
**XAC0303**	A0CE6829E06	I.D.2	transcriptional regulator
**XAC0305**	A0UT6815E09	II.D.10	gamma-glutamyltranspeptidase
**XAC0310**	A0QH6810C02	I.A.2	vanillate O-demethylase oxidoreductase
**XAC0311**	A0AM1363H10	I.A.2	vanillate O-demethylase oxygenasesubunit
**XAC0312**	A0UV6509H09	I.D.2	transcriptional regulator lysR family
**XAC0314**	A0QR6840F09	VIII.A	conserved hypothetical protein
**XAC0315**	A0UV9712G03	VIII.B	hypothetical protein
**XAC0316**	A0QR6804B09	I.D.2	transcriptional regulator lysR family
**XAC0317**	A0UV6711G02	V.A.7	MFS transporter
**XAC0319**	A0QR6804C09	IX	chloroperoxidase
**XAC0320**	A0QR6728B03	I.D.2	SIR2-like regulatory protein
**XAC0323**	A0QR6728D05	VIII.C	Xanthomonas conserved hypothetical protein
**XAC0326**	A0JJ0702A07	I.D.1	two-component system, sensor protein
**XAC0327**	A0UV6708C10	VII.C	acriflavin resistance protein
**XAC0328**	A0QH6731B06	VII.C	multidrug efflux transporter
**XAC0329**	A0QH6810B12	IV.A.2	outer membrane protein
**XAC0330**	genomic_DNA	VIII.A	conserved hypothetical protein
**XAC0330**	A0QR5317B07	I.D.2	conditioned medium factor
**XAC0333**	A0UT9846A01	I.D.2	transcriptional regulator metE/metH family
**XAC0334**	A0UV9822C06	I.C.3	NADH-dependent FMN reductase
**XAC0339**	A0QR6730C01	I.C.3	oxidoreductase
**XAC0341**	A0QR6728C07	VIII.A	conserved hypothetical protein
**XAC0342**	A0AC9832C06	VI.C	ISxac3 transposase
**XAC0343**	A0UT6817D07	VI.C	ISxac3 transposase
**XAC0344**	A0QH6835D01	VI.A	phage-related integrase
**XAC0345**	A0EC6472A01	II.A.2	dihydroxy-acid dehydratase
**XAC0346**	A0QR6724G10	VII.D	cellulase (degenerated)
**XAC0347**	A0UT9894B03	III.C.3	proteinase inhibitor
**XAC0348**	A0QR6403B06	IX	transferase
**XAC0349**	A0AR1103D07	V.A.7	MFS transporter
**XAC0350**	A0UT9876H09	VIII.A	conserved hypothetical protein
**XAC0354**	A0AC0115A10	I.A.2	benzaldehyde dehydrogenase II
**XAC0355**	A0QR6701H06	I.D.2	PobR regulator
**XAC0356**	A0UV6739F09	I.A.2	P-hydroxybenzoate hydroxylase
**XAC0358**	A0RN1228F09	I.B.10	glycerol kinase
**XAC0360**	A0JE1211B06	I.A.2	glycerol-3-phosphate dehydrogenase
**XAC0362**	A0QR6729B08	I.A.2	phenoxybenzoate dioxygenase beta subunit
**XAC0364**	A0UV6766G04	I.C.2	glutaconate CoA transferase subunit A
**XAC0368**	A0AC6825E03	I.A.2	protocatechuate 3,4-dioxygenase alpha chain
**XAC0370**	A0QR9758G03	I.A.2	b-ketoadipate enol-lactone hydrolase
**XAC0372**	A0QR6737F10	III.D.2	hydrolase
**XAC0373**	A0AC6360F08	I.D.2	transcriptional regulator
**XAC0374**	A0QR9756C08	VIII.A	conserved hypothetical protein
**XAC0375**	A0QR5903C07	III.D.2	lipase
**XAC0376**	A0UT6817G03	VIII.A	conserved hypothetical protein
**XAC0380**	A0QR5408H07	VIII.A	conserved hypothetical protein

**XAC0382**	A0QR5323F11	II.A.2	aspartyl/asparaginyl beta-hydroxylase
**XAC0385**	A0UV6764C05	II.D.1	biotin biosynthesis protein
**XAC0387**	A0QR6107C02	II.D.1	8-amino-7-oxononanoate synthase
**XAC0388**	A0QR5902B06	II.D.1	biotin synthase
**XAC0389**	A0UV6733G07	VII.F	competence protein F
**XAC0390**	genomic_DNA	VIII.B	hypothetical protein
**XAC0391**	genomic_DNA	VIII.B	hypothetical protein
**XAC0392**	A0QR5704G08	VIII.B	hypothetical protein
**XAC0393**	genomic_DNA	VII.H	HpaF protein
**XAC0393**	A0EC1093D06	VII.B	HpaF protein
**XAC0394**	genomic_DNA	VII.B	HrpF protein
**XAC0394**	A0UT5316A09	VII.B	HrpF protein
**XAC0395**	genomic_DNA	VIII.B	hypothetical protein
**XAC0396**	genomic_DNA	VII.B	HpaB protein
**XAC0396**	A0QR6613B03	VII.B	HpaB protein
**XAC0397**	genomic_DNA	VII.B	HrpE protein
**XAC0398**	A0AC0113A01	VII.B	HrpD6 protein
**XAC0398**	genomic_DNA	VII.B	HrpD6 protein
**XAC0399**	genomic_DNA	VII.B	HrpD5 protein
**XAC0400**	genomic_DNA	VII.B	HpaA protein
**XAC0401**	genomic_DNA	VII.B	HrcS protein
**XAC0401**	A0JJ1016G09	VII.B	HrcS protein
**XAC0403**	A0CE9842G03	VII.B	HrcQ protein
**XAC0403**	genomic_DNA	VII.B	HrcQ protein
**XAC0404**	genomic_DNA	VII.B	HpaP protein
**XAC0405**	genomic_DNA	VII.B	HrcV protein
**XAC0405**	A0CE6342H06	VII.B	HrcV protein
**XAC0406**	genomic_DNA	VII.B	HrcU protein
**XAC0406**	A0QR6839E11	VII.B	HrcU protein
**XAC0407**	genomic_DNA	VII.B	HrpB1 protein
**XAC0407**	A0QR6002G09	VII.B	HrpB1 protein
**XAC0408**	genomic_DNA	VII.B	HrpB2 protein
**XAC0408**	A0UV6818E12	VII.B	HrpB2 protein
**XAC0410**	genomic_DNA	VII.B	HrpB4 protein
**XAC0411**	genomic_DNA	VII.B	HrpB5 protein
**XAC0411**	A0CE6315H09	VII.B	HrpB5 protein
**XAC0412**	genomic_DNA	VII.B	HrcN protein
**XAC0413**	genomic_DNA	VII.B	HrpB7 protein
**XAC0416**	genomic_DNA	VII.B	Hpa1 protein
**XAC0416**	A0UV6845E05	VII.B	Hpa1 protein
**XAC0417**	genomic_DNA	VII.B	Hpa2 protein
**XAC0418**	A0QR5902F03	VIII.C	Xanthomonas conserved hypothetical protein
**XAC0419**	A0QR5206B06	VIII.C	Xanthomonas conserved hypothetical protein
**XAC0420**	A0UV6734C11	VIII.A	conserved hypothetical protein
**XAC0421**	A0UV6765C11	IV.B	phosphoglycerol transferase I
**XAC0422**	A0RN1632F03	V.A.7	ABC transporter substrate binding protein
**XAC0423**	A0QR6802B09	VIII.A	conserved hypothetical protein
**XAC0424**	A0UV6739C11	VIII.A	conserved hypothetical protein
**XAC0425**	A0QR6389B08	III.D.1	glycogen synthase
**XAC0427**	A0QR9825E08	I.A.1	maltooligosyltrehalose trehalohydrolase
**XAC0432**	A0QH6309F03	VIII.A	conserved hypothetical protein
**XAC0435**	A0UV5308B10	VII.H	VirK protein
**XAC0437**	A0AM1393H03	I.D.2	transcriptional regulator tetR/acrR family
**XAC0438**	A0QR6730H07	V.A.7	component of multidrug efflux system

**XAC0439**	A0JJ1073F04	V.A.7	cation efflux system protein
**XAC0440**	A0UV6458D02	I.C.3	oxidoreductase
**XAC0441**	A0AC9764C06	VIII.C	Xanthomonas conserved hypothetical protein
**XAC0442**	A0AM1113C08	III.B.5	ATP-dependent RNA helicase
**XAC0443**	A0QR5704B06	I.C.6	dihydrolipoamide acyltransferase
**XAC0444**	A0RN1631E12	VIII.A	conserved hypothetical protein
**XAC0447**	A0QR5529G06	VII.F	nuclease
**XAC0448**	A0QR9776G05	II.D.17	tryptophan 2,3-dioxygenase
**XAC0449**	A0QR6802F08	V.A.1	di-tripeptide transporter
**XAC0450**	A0RN1627B01	VIII.A	conserved hypothetical protein
**XAC0453**	A0UV9750A07	VIII.A	conserved hypothetical protein
**XAC0457**	A0UV6206E05	VIII.A	conserved hypothetical protein
**XAC0458**	A0UV6843C11	VII.G	PhaF protein
**XAC0459**	A0UV6736E08	VII.G	PhaE protein
**XAC0460**	A0JJ1591D03	VII.G	PhaD protein
**XAC0464**	A0QH9769H04	VIII.A	conserved hypothetical protein
**XAC0468**	A0UV6736A06	VIII.A	conserved hypothetical protein
**XAC0469**	A0UT6817B01	VIII.C	Xanthomonas conserved hypothetical protein
**XAC0470**	A0UV6762F05	II.B.1	phosphoribosylaminoimidazole-succinocarboxamide synthase
**XAC0472**	A0AC6447F05	I.B.6	D-ribulose-5-phosphate 3-epimerase
**XAC0473**	A0QR6725B06	IV.A.1	membrane protein
**XAC0475**	A0UV5516B01	VIII.A	conserved hypothetical protein
**XAC0476**	A0QH6810D05	II.A.4	anthranilate synthase component I
**XAC0477**	A0QR6730C08	II.A.3	threonine aldolase
**XAC0478**	A0JJ1132B08	II.A.4	anthranilate synthase component II
**XAC0479**	A0AC6825E12	VIII.A	conserved hypothetical protein
**XAC0480**	A0QR6330B12	II.A.4	anthranilate synthase component II
**XAC0481**	A0UV6707B07	II.A.4	indole-3-glycerol phosphate synthase
**XAC0483**	A0QH6813E03	VII.H	CAP-like protein
**XAC0484**	A0JE5637B03	II.F	S-adenosyl methionine decarboxylase proenzyme
**XAC0485**	A0UV6711H10	III.C.2	SugE protein
**XAC0488**	A0UV6205H02	III.B.2	30S ribosomal protein S9
**XAC0491**	A0QR6002A09	I.B.7	probable (di)nucleoside polyphosphate hydrolase
**XAC0492**	A0QR5704G02	I.C.3	bacterioferritin-associated ferredoxin
**XAC0495**	A0UV6766C02	I.D.1	two-component system, regulatory protein
**XAC0497**	A0JJ1056C05	VIII.A	conserved hypothetical protein
**XAC0498**	A0UV6364F04	VIII.A	conserved hypothetical protein
**XAC0500**	A0QR6737B06	VIII.B	hypothetical protein
**XAC0501**	A0UV6735E05	VIII.A	conserved hypothetical protein
**XAC0502**	A0UT9876A01	VI.C	ISxac1 transposase
**XAC0504**	A0QH6836C08	IV.A.1	transmembrane protein
**XAC0507**	A0UV6766F03	III.D.2	2-acylglycerophosphoethanolamine acyltransferase
**XAC0510**	A0QR5407A08	VIII.A	conserved hypothetical protein
**XAC0512**	A0UV6707B09	VIII.B	hypothetical protein
**XAC0513**	A0QR6392E10	II.B.1	bifunctional purine biosynthesis protein
**XAC0516**	A0UV9904E04	VIII.A	conserved hypothetical protein
**XAC0517**	A0EC6324F06	VIII.A	conserved hypothetical protein
**XAC0518**	A0QR9759B02	VIII.C	Xanthomonas conserved hypothetical protein
**XAC0519**	A0AC6337H03	II.E	CDP-diacylglycerol--glycerol-3-phosphate 3-phosphatidyltransferase-related protein
**XAC0520**	A0UV9748A06	IV.C	acyltransferase
**XAC0521**	A0AC0113F06	I.B.8	phosphatidate cytidiltransferase
**XAC0526**	A0RN1324B11	III.B.3	ribosomal protein L11 methyltransferase
**XAC0527**	A0JE1147G09	VIII.B	hypothetical protein
**XAC0529**	A0QR5106D10	VIII.C	Xanthomonas conserved hypothetical protein

**XAC0530**	A0UV9884D05	II.E	biotin carboxylase subunit of acetyl CoA carboxylase
**XAC0532**	A0QR5702F05	II.E	biotin carboxyl carrier protein of acetyl-CoA carboxilase
**XAC0533**	A0UV6820G04	II.A.4	catabolic dehydroquinase
**XAC0536**	A0UV6818E05	VII.H	virulence regulating protein
**XAC0538**	A0AC6408F04	VIII.B	hypothetical protein
**XAC0539**	A0UT9876D05	I.C.3	oxidoreductase
**XAC0540**	A0UE6718F07	III.B.6	ribonuclease
**XAC0544**	A0RN1457A07	VIII.A	conserved hypothetical protein
**XAC0545**	A0QR9859F12	II.A.4	phospho-2-dehydro-3-deoxyheptonate aldolase, phe-sensitive
**XAC0546**	A0QH6835G08	VIII.C	Xanthomonas conserved hypothetical protein
**XAC0547**	A0QH6836D01	VIII.C	Xanthomonas conserved hypothetical protein
**XAC0548**	A0UV6711C07	II.C	gluconolactonase precursor
**XAC0549**	A0UV6733A07	VIII.C	Xanthomonas conserved hypothetical protein
**XAC0550**	A0UT9846G07	I.D.4	glutamine synthetase adenylyltransferase
**XAC0552**	A0CE6743E08	III.C.3	proteinase
**XAC0553**	A0AC1335A10	VIII.A	conserved hypothetical protein
**XAC0554**	A0UV6843H07	IX	nitroreductase
**XAC0555**	A0UV6818H09	VIII.A	conserved hypothetical protein
**XAC0556**	A0UV6762D04	VIII.B	hypothetical protein
**XAC0558**	A0QR6727H11	V.A.7	iron utilization protein
**XAC0560**	A0UT1518G10	II.E	alpha subunit of malonate decarboxylase
**XAC0561**	A0UV9805C11	II.E	delta subunit of malonate decarboxylase
**XAC0562**	A0QR9756D01	II.D.1	beta subunit of malonate decarboxylase
**XAC0564**	A0QR6389B09	II.D.1	malonate decarboxylase
**XAC0566**	A0UV6736D08	II.E	malonyl CoA-ACP transacylase
**XAC0567**	A0QR6749E10	V.A.3	dicarboxylate carrier protein
**XAC0570**	A0QR6483H09	I.D.4	anti-sigma F factor antagonist
**XAC0570**	genomic_DNA	I.D.4	anti-sigma F factor antagonist
**XAC0571**	A0QR5904H08	VIII.A	conserved hypothetical protein
**XAC0572**	A0QR9758C10	IX	IcfG protein
**XAC0573**	A0QR6002C11	VIII.C	Xanthomonas conserved hypothetical protein
**XAC0575**	A0CE6830E03	I.A.1	arabinogalactan endo-1,4-beta-galactosidase
**XAC0576**	A0QR5903D03	I.C.6	pyruvate dehydrogenase
**XAC0577**	A0UV6455E06	III.A.5	cytosine methyltransferase
**XAC0578**	A0QH6309D10	VI.C	ISxac3 transposase
**XAC0581**	A0JJ1056H09	I.D.2	transcriptional regulator araC family
**XAC0583**	A0AC6439C06	I.C.3	oxidoreductase
**XAC0584**	A0UV6514F12	VIII.B	hypothetical protein
**XAC0585**	A0QR6748F06	VIII.A	conserved hypothetical protein
**XAC0586**	A0QR9767H07	VIII.A	conserved hypothetical protein
**XAC0587**	A0QR5321D02	VIII.A	conserved hypothetical protein
**XAC0588**	A0UV6765H03	VIII.B	hypothetical protein
**XAC0591**	A0UV6711G03	III.C.3	dipeptidyl peptidase IV
**XAC0592**	A0UV9872B12	VIII.A	conserved hypothetical protein
**XAC0593**	A0QH6732E10	VIII.A	conserved hypothetical protein
**XAC0594**	A0JJ1541F09	VIII.C	Xanthomonas conserved hypothetical protein
**XAC0599**	A0UE6718F11	VIII.B	hypothetical protein
**XAC0601**	A0QH9769B02	VIII.B	hypothetical protein
**XAC0603**	A0AC6825C11	VIII.A	conserved hypothetical protein
**XAC0605**	A0EC6316B08	VIII.A	conserved hypothetical protein
**XAC0609**	A0UV6739A08	III.C.3	zinc protease
**XAC0610**	A0CE1624D01	I.D.3	histidine kinase/response regulator hybrid protein
**XAC0612**	A0QR5902D04	VII.D	cellulase
**XAC0616**	A0RN1447G01	VIII.B	hypothetical protein

**XAC0617**	A0UV6710G03	VIII.B	hypothetical protein
**XAC0618**	A0QR9767D08	VII.G	periplasmic glucan biosynthesis protein
**XAC0619**	A0UV9880G04	IX	carboxylesterase
**XAC0623**	A0UV6762G07	VIII.A	conserved hypothetical protein
**XAC0625**	A0QH6810G03	VIII.A	conserved hypothetical protein
**XAC0630**	A0AC6823G11	II.A.2	aminotransferase
**XAC0631**	A0EC6426B09	III.C.3	protease II (oligopeptidase B)
**XAC0633**	A0QR9739H11	VIII.A	conserved hypothetical protein
**XAC0634**	A0CE6480F07	II.A.2	diaminopimelate epimerase
**XAC0635**	A0QR6102H01	VIII.A	conserved hypothetical protein
**XAC0636**	A0UV6820D02	III.A.3	site-specific recombinase
**XAC0638**	A0QR6002C12	III.C.3	ATP-dependent HslUV protease ATP-binding subunit HslU
**XAC0641**	A0UV9882C07	VII.C	multidrug resistance efflux pump
**XAC0642**	A0QR6752G05	V.A.7	MFS transporter
**XAC0643**	A0UV6766E02	II.D.11	ubiquinone/menaquinone transferase
**XAC0646**	A0QR6752B04	VIII.C	Xanthomonas conserved hypothetical protein
**XAC0647**	A0QR5903A09	VIII.A	conserved hypothetical protein
**XAC0651**	A0UV9803D04	IV.C	surface antigen gene
**XAC0652**	A0EC0311G07	I.C.2	alcohol dehydrogenase class III
**XAC0653**	A0UV6710B09	V.A.7	TonB-dependent receptor
**XAC0656**	A0QR6730B09	IV.B	rod shape-determining protein
**XAC0658**	A0QR6725F07	IV.B	rod shape-determining protein
**XAC0659**	A0AC6358F03	IV.B	penicillin-binding protein 2
**XAC0660**	A0QR9825D02	IV.A.1	rod shape-determining protein
**XAC0663**	A0RN1497B05	III.D.3	rare lipoprotein A
**XAC0664**	A0CE9727H07	IV.A.1	penicillin-binding protein 6
**XAC0665**	A0UV6709H04	VIII.A	conserved hypothetical protein
**XAC0667**	A0UV6844B10	II.D.3	lipoate biosynthesis protein B
**XAC0668**	A0UV6736B07	II.D.3	lipoic acid synthetase
**XAC0669**	A0UV1613G04	III.C.3	tail-specific protease
**XAC0670**	A0AC0113G03	VIII.A	conserved hypothetical protein
**XAC0671**	A0UV1358D08	I.D.2	transcriptional regulator
**XAC0672**	A0JE1379F06	VIII.A	conserved hypothetical protein
**XAC0674**	A0UV6711H03	IX	pre-B cell enhancing factor related protein
**XAC0674**	genomic_DNA	IX	pre-B cell enhancing factor related protein
**XAC0676**	A0QH6309B08	VIII.C	Xanthomonas conserved hypothetical protein
**XAC0677**	genomic_DNA	VIII.C	Xanthomonas conserved hypothetical protein
**XAC0677**	A0UV6733G02	VIII.C	Xanthomonas conserved hypothetical protein
**XAC0678**	A0UV6821H03	VIII.A	conserved hypothetical protein
**XAC0679**	A0UV6761C11	VIII.C	Xanthomonas conserved hypothetical protein
**XAC0681**	A0UT9848F09	I.C.3	oxidoreductase
**XAC0682**	A0JJ1038G03	VIII.A	conserved hypothetical protein
**XAC0684**	A0QR5902H03	I.D.1	two-component system, regulatory protein
**XAC0685**	A0JJ1311A02	I.D.3	histidine kinase/response regulator hybrid protein
**XAC0687**	A0QR6377E07	VIII.A	conserved hypothetical protein
**XAC0690**	A0UV6738A04	V.A.7	TonB-dependent receptor
**XAC0691**	A0QH6732D05	VIII.A	conserved hypothetical protein
**XAC0692**	A0QR5902G07	VIII.A	conserved hypothetical protein
**XAC0694**	A0QR6730D07	VII.H	type II secretion system protein C
**XAC0695**	A0QR6730B11	VII.H	type II secretion system protein D
**XAC0697**	A0UV6735E08	VII.H	type II secretion system protein F
**XAC0697**	A0AC0115A12	VII.H	type II secretion system protein F
**XAC0699**	A0AC6357C10	VII.H	type II secretion system protein H
**XAC0700**	A0QR6002A03	VII.H	type II secretion system protein I

**XAC0701**	A0UV6707H02	VII.H	type II secretion system protein J
**XAC0704**	A0UV6843H03	VII.H	type II secretion system protein M
**XAC0707**	A0UV6766A07	I.A.1	beta-galactosidase (truncated)
**XAC0709**	A0QH6732A04	III.D.1	N-acetylglucosaminidase
**XAC0713**	A0AC6825F07	I.D.2	transcriptional regulator lacI family
**XAC0716**	A0QR6728F09	V.A.7	TonB-dependent receptor
**XAC0717**	A0UV6736H01	III.B.4	tRNA nucleotidyltransferase
**XAC0720**	A0QR9776E07	V.A.7	high-affinity choline transport
**XAC0722**	A0UT9703A10	III.C.1	thiol:disulfide interchange protein
**XAC0725**	A0EC6322D06	IX	GTP-binding protein
**XAC0727**	A0QH6810E01	I.D.2	transcriptional regulator
**XAC0728**	A0QR6801C12	II.D.10	glutamate-cysteine ligase precursor
**XAC0730**	A0UV6712E01	I.D.1	two-component system, regulatory protein
**XAC0738**	A0CE0105A07	I.C.3	oxidoreductase
**XAC0739**	A0QR6705C04	VIII.A	conserved hypothetical protein
**XAC0740**	A0AC6825H08	VIII.C	Xanthomonas conserved hypothetical protein
**XAC0741**	A0AC6336B05	V.A.7	ABC transporter ATP-binding protein
**XAC0741**	A0QR9739G05	V.A.7	ABC transporter ATP-binding protein
**XAC0743**	A0AC6358D03	II.A.3	serine hydroxymethyltransferase
**XAC0744**	A0UV6709F09	VIII.A	conserved hypothetical protein
**XAC0745**	A0QR6729G04	IV.C	acetyltransferase
**XAC0747**	A0JE1466A02	VIII.A	conserved hypothetical protein
**XAC0748**	A0UV6843A02	II.D.9	riboflavin synthase alpha chain
**XAC0750**	A0UT6817E08	II.D.9	6,7-dimethyl-8-ribityllumazine synthase
**XAC0753**	A0UT6814F07	VIII.A	conserved hypothetical protein
**XAC0754**	A0UV6766E12	VIII.B	hypothetical protein
**XAC0756**	A0QR6702B04	V.A.7	potassium-transporting ATPase, A chain
**XAC0757**	A0AM1592A02	V.A.7	potassium-transporting ATPase, B chain
**XAC0758**	A0QR5323E10	V.A.7	potassium-transporting ATPase, C chain
**XAC0759**	A0JJ1197D07	I.D.1	two-component system, sensor protein
**XAC0762**	A0QR9814G03	I.C.2	D-lactate dehydrogenase
**XAC0764**	A0QR9776E04	VIII.A	conserved hypothetical protein
**XAC0765**	A0UV6708H11	VIII.A	conserved hypothetical protein
**XAC0766**	A0UV5525H03	VIII.A	conserved hypothetical protein
**XAC0768**	A0QR6443E11	VIII.C	Xanthomonas conserved hypothetical protein
**XAC0770**	A0UV6843B06	VIII.A	conserved hypothetical protein
**XAC0773**	A0UV5525C11	V.B	cell division protein
**XAC0774**	A0CE9842A04	IV.B	penicillin-binding protein 3
**XAC0775**	A0CE6743H10	IV.B	UDP-N-acetylmuramoylalanyl-D-glutamate--2,6-diaminopimelate ligase
**XAC0778**	A0UE6464G09	V.B	cell division protein
**XAC0779**	A0JJ1101C04	IV.B	UDP-acetylglucosamine-N-acetylmuramyl-(pentapeptide)pyrophosphoryl-undecaprenol
**XAC0780**	A0QH6308G12	IV.B	UDP-N-acetylmuramate--alanine ligase
**XAC0783**	A0QR6804C07	V.B	cell division protein
**XAC0784**	A0CE6743B07	V.B	cell division protein
**XAC0787**	A0UV6349G11	III.C.3	peptidase
**XAC0788**	A0UE6718F01	V.A.6	preprotein translocase SecA subunit
**XAC0790**	A0QR6002E10	VIII.C	Xanthomonas conserved hypothetical protein
**XAC0791**	A0AM1393B06	II.A.2	5,10-methylenetetrahydrofolate reductase
**XAC0792**	A0QR9753C01	VIII.A	conserved hypothetical protein
**XAC0795**	A0UV6708B07	III.C.3	protease
**XAC0796**	A0JJ1342A04	VIII.B	hypothetical protein
**XAC0797**	A0AC6823F12	I.A.2	D-galactose 1-dehydrogenase
**XAC0799**	A0UV6710G07	VIII.A	conserved hypothetical protein

**XAC0800**	A0UT9701H12	VIII.C	Xanthomonas conserved hypothetical protein
**XAC0806**	A0AM1540D10	I.C.7	phosphoenolpyruvate carboxylase
**XAC0807**	A0QR6705D04	I.D.2	transcriptional regulator tetR/acrR family
**XAC0808**	A0QR6702E10	VIII.C	Xanthomonas conserved hypothetical protein
**XAC0810**	A0QR6489A09	VIII.C	Xanthomonas conserved hypothetical protein
**XAC0811**	A0JE1149B03	V.A.7	TonB-dependent receptor
**XAC0812**	A0RN1548A04	I.B.10	phosphoanhydride phosphohydrolase
**XAC0814**	A0QR9759B03	VIII.A	conserved hypothetical protein
**XAC0815**	A0UV6820D05	IX	methyltransferase
**XAC0816**	A0AC6827B04	VIII.A	conserved hypothetical protein
**XAC0817**	A0QR5104G09	VIII.B	hypothetical protein
**XAC0818**	A0QH6380E01	I.A.2	ribokinase
**XAC0820**	A0AC6450H03	VIII.B	hypothetical protein
**XAC0822**	A0UV6733D04	VIII.C	Xanthomonas conserved hypothetical protein
**XAC0826**	A0JJ1382F06	VIII.A	conserved hypothetical protein
**XAC0829**	A0UV9905F12	V.A.7	ABC transporter substrate binding protein
**XAC0830**	A0QR5512D08	I.B.12	taurine dioxygenase
**XAC0831**	A0UT9876A06	I.C.3	oxidoreductase
**XAC0833**	A0AC0113H01	II.E	acyl-CoA thioesterase I
**XAC0834**	A0UV9803E05	I.D.1	two-component system, regulatory protein
**XAC0835**	A0QR5701E06	I.D.1	two-component system, sensor protein
**XAC0838**	A0UV9871G08	VIII.A	conserved hypothetical protein
**XAC0840**	A0RN1587E01	VIII.A	conserved hypothetical protein
**XAC0843**	A0AC6429C08	VIII.B	hypothetical protein
**XAC0846**	A0UV9883G09	I.A.2	FMNH2-dependent monooxygenase
**XAC0847**	A0UV6435A01	V.A.7	ABC transporter ATP-binding protein
**XAC0849**	A0QR6730F05	V.A.7	sulfonate binding protein
**XAC0852**	A0UV6709D10	VII.C	TonB-dependent receptor
**XAC0853**	A0CE6480F04	VIII.B	hypothetical protein
**XAC0857**	A0QR6303E10	V.A.4	ABC transporter permease
**XAC0858**	A0QR6489D01	V.A.7	ABC transporter permease
**XAC0860**	A0JJ1536D10	V.A.1	ABC transporter ATP-binding protein
**XAC0861**	A0QH6313D01	II.B.4	diadenosine tetraphosphatase
**XAC0864**	A0QR5323G04	II.D.6	pyridoxal phosphate biosynthetic protein
**XAC0866**	A0QR5322C07	VII.C	organic solvent tolerance precursor
**XAC0870**	A0UV1534F03	VIII.A	conserved hypothetical protein
**XAC0871**	A0QR9717G08	VIII.C	Xanthomonas conserved hypothetical protein
**XAC0873**	A0CE9723F08	IX	VisC protein
**XAC0875**	A0QR6728H07	VIII.C	Xanthomonas conserved hypothetical protein
**XAC0876**	A0QR9815A01	VIII.A	conserved hypothetical protein
**XAC0877**	A0QR6705D03	I.D.2	transcriptional regulator gntR family
**XAC0878**	A0JJ0102D03	I.A.2	protocatechuate 3,4-dioxygenase beta chain
**XAC0880**	A0UV6709G01	I.D.2	transcriptional regulator
**XAC0882**	A0UV6349B08	I.C.3	aldehyde dehydrogenase
**XAC0885**	A0UV6710D12	VIII.C	Xanthomonas conserved hypothetical protein
**XAC0886**	A0UV6843H12	VIII.C	Xanthomonas conserved hypothetical protein
**XAC0888**	A0QR5704F07	V.D	glucose-fructose oxidoreductase
**XAC0890**	A0CE9842E01	VIII.A	conserved hypothetical protein
**XAC0891**	A0QR5311C09	VIII.B	hypothetical protein
**XAC0894**	A0RN1433D11	VII.C	glutathione S-transferase
**XAC0895**	A0CE9841H03	VIII.C	Xanthomonas conserved hypothetical protein
**XAC0896**	A0QR9814B08	I.D.1	two-component system, regulatory protein
**XAC0897**	A0QR6391F05	I.D.1	two-component system, sensor protein
**XAC0898**	A0UV6738G03	I.D.1	two-component system, regulatory protein

**XAC0899**	A0AC0115A07	VIII.A	conserved hypothetical protein
**XAC0900**	A0AC6822D08	III.C.1	peptide methionine sulfoxide reductase
**XAC0901**	A0QR6376B01	VIII.A	conserved hypothetical protein
**XAC0903**	A0UT9894D12	I.D.2	regulator of nucleoside diphosphate kinase
**XAC0904**	A0UV1502E01	VIII.A	conserved hypothetical protein
**XAC0905**	A0QR9757D08	I.D.2	oxidative stress transcriptional regulator
**XAC0907**	A0QR6768A02	VII.C	alkyl hydroperoxide reductase subu
**XAC0908**	A0UV6734G12	II.D.12	protoporphyrinogen oxidase
**XAC0910**	A0UV6708B01	VIII.A	conserved hypothetical protein
**XAC0913**	A0AC0113A06	VIII.A	conserved hypothetical protein
**XAC0915**	A0JJ0104E10	VIII.A	conserved hypothetical protein
**XAC0917**	A0UV6733A03	I.D.2	transcriptional regulator
**XAC0918**	A0CE6743B10	I.B.10	pyridine nucleotide transhydrogenase
**XAC0919**	A0JJ0102D01	I.B.10	pyridine nucleotide transhydrogenase
**XAC0920**	A0UT6817C06	VIII.C	Xanthomonas conserved hypothetical protein
**XAC0922**	genomic_DNA	I.D.4	ECF sigma factor
**XAC0923**	A0JJ1382C08	I.B.10	pyridine nucleotide transhydrogenase, subunit alpha
**XAC0925**	A0UV6734E07	VIII.A	conserved hypothetical protein
**XAC0926**	A0UV6708E12	VIII.A	conserved hypothetical protein
**XAC0927**	A0UV9900F09	II.A.2	branched-chain amino acid aminotransferase
**XAC0928**	A0QR9776G09	III.C.3	extracellular protease
**XAC0929**	A0AC6822D09	III.C.3	extracellular protease
**XAC0930**	A0QR6730F02	III.C.3	extracellular protease
**XAC0931**	A0UV6710H08	I.D.2	transcriptional regulator
**XAC0933**	A0UE9888B07	I.A.1	xylanase (truncated)
**XAC0935**	A0CE6740D11	VIII.C	Xanthomonas conserved hypothetical protein
**XAC0937**	A0UV6766B12	III.B.6	ribonuclease BN
**XAC0938**	A0QR6730A07	II.D.10	thioredoxin
**XAC0940**	A0UV6843C09	VIII.A	conserved hypothetical protein
**XAC0941**	A0UV6820A02	I.D.2	transcriptional regulator
**XAC0942**	A0QR6737D11	II.D.4	molybdopterin biosynthesis protein B
**XAC0944**	A0UV5523D08	III.C.1	peptide chain release factor 1
**XAC0948**	A0UV9903D07	I.B.10	4-diphosphocytidyl-2-C-methyl-D-erythritol kinase
**XAC0950**	A0CE6830D06	II.B.1	phosphoribosyl pyrophosphate synthetase
**XAC0951**	A0QR6803C01	III.B.2	50S ribosomal protein L25
**XAC0957**	A0QR5902F09	III.C.1	elongation factor Tu
**XAC0959**	A0QR6727H12	V.A.6	preprotein translocase subunit
**XAC0960**	A0UT6815D06	III.B.5	transcription antitermination factor
**XAC0963**	A0AC0115C03	III.B.2	50S ribosomal protein L10
**XAC0966**	A0UV6739G11	III.B.5	RNA polymerase beta' subunit
**XAC0968**	A0QR9732D01	III.B.2	30S ribosomal protein S7
**XAC0969**	A0CE6828D06	III.C.1	elongation factor G
**XAC0970**	A0UV6710B01	III.C.1	elongation factor Tu
**XAC0972**	A0UV9882H06	III.B.2	50S ribosomal protein L3
**XAC0974**	A0UV1503C09	III.B.2	50S ribosomal protein L23
**XAC0975**	A0QR6802H01	III.B.2	50S ribosomal protein L2
**XAC0976**	A0UE6610B04	III.B.2	30S ribosomal protein S19
**XAC0977**	A0UV6362F06	III.B.2	50S ribosomal protein L22
**XAC0978**	A0QR5701A03	III.B.2	30S ribosomal protein S3
**XAC0979**	A0UV6764E11	III.B.2	50S ribosomal protein L16
**XAC0981**	A0QR5302A08	III.B.2	30S ribosomal protein S17
**XAC0982**	A0UE6759A02	III.B.2	50S ribosomal protein L14
**XAC0983**	A0UT9703H01	III.B.2	50S ribosomal protein L24
**XAC0986**	A0UV5502B11	III.B.2	30S ribosomal protein S8

**XAC0987**	A0AM1595G04	III.B.2	50S ribosomal protein L6
**XAC0990**	A0QH6383E03	III.B.2	50S ribosomal protein L30
**XAC0991**	A0AC6430D09	III.B.2	50S ribosomal protein L15
**XAC0992**	A0UT9875G10	V.A.6	preprotein translocase SecY subunit
**XAC0997**	A0AC6450B03	III.B.2	50S ribosomal protein L17
**XAC0999**	A0UV6736E09	IV.A.2	colicin I receptor
**XAC1000**	A0UE9857B11	II.A.4	family II 2-keto-3-deoxy-D-arabino-heptulosonate 7-phosphate synthase
**XAC1001**	A0QR6393C05	IV.A.1	membrane protein
**XAC1002**	A0QR9767D03	III.B.4	Glu-tRNAGln amidotransferase A subunit
**XAC1003**	A0QR6749G10	VIII.A	conserved hypothetical protein
**XAC1004**	A0RN1171D08	V.B	GTP-binding elongation factor protein
**XAC1005**	A0QR6391G07	III.C.1	peptidyl-prolyl cis-trans isomerase
**XAC1006**	A0AC6825E05	I.C.7	malate dehydrogenase
**XAC1007**	A0JE5637E12	VII.C	glutathione S-transferase
**XAC1008**	A0UV6733H05	VIII.A	conserved hypothetical protein
**XAC1009**	A0QR5904G10	VIII.B	hypothetical protein
**XAC1010**	A0AM1616D10	I.A.2	2,4-dienoyl-CoA reductase
**XAC1011**	A0QR9757E02	I.C.3	oxidoreductase
**XAC1013**	A0QR5701F12	VIII.A	conserved hypothetical protein
**XAC1015**	A0QH6313F08	VIII.C	Xanthomonas conserved hypothetical protein
**XAC1016**	A0QR9706E11	II.A.3	2-amino-3-ketobutyrate CoA ligase
**XAC1018**	A0QR5317A08	V.A.2	ABC transporter sulfate permease
**XAC1020**	A0UT9849H09	V.A.2	sulfate ABC transporter ATP-binding protein
**XAC1023**	A0QR9864A12	V.A.7	TonB-dependent receptor
**XAC1024**	A0UT9850B07	III.D.2	non-hemolytic phospholipase C
**XAC1026**	A0QR6803C02	VIII.A	conserved hypothetical protein
**XAC1030**	A0QR6729D10	VIII.A	conserved hypothetical protein
**XAC1031**	A0UT9891C04	VII.C	colicin V production protein
**XAC1032**	A0AC6430G09	II.B.1	amidophosphoribosyltransferase
**XAC1034**	A0UV6845D06	III.C.3	peptidyl-Asp metalloendopeptidase
**XAC1036**	A0UE9858G06	VIII.A	conserved hypothetical protein
**XAC1037**	A0QR9860E02	VIII.A	conserved hypothetical protein
**XAC1038**	A0QH6813C11	IV.C	glycosyl transferase
**XAC1039**	A0UE6398F12	I.B.9	exopolyphosphatase
**XAC1047**	A0CE6315A06	VIII.A	conserved hypothetical protein
**XAC1052**	A0QR9732E07	VI.C	ISxac3 transposase
**XAC1053**	A0UV9900G05	VI.C	ISxac3 transposase
**XAC1054**	A0CE6742D11	VI.A	integrase
**XAC1055**	A0UV6734H03	VIII.B	hypothetical protein
**XAC1057**	A0QR6377B03	VIII.B	hypothetical protein
**XAC1058**	A0QR6805F07	VIII.B	hypothetical protein
**XAC1061**	A0QR5520B10	VIII.A	conserved hypothetical protein
**XAC1062**	A0UV6846A04	VIII.B	hypothetical protein
**XAC1063**	A0QR5704E09	VI.A	phage-related lysozyme
**XAC1064**	A0AC0113D01	VI.A	phage-related DNA maturase
**XAC1065**	A0AC6356G07	VIII.B	hypothetical protein
**XAC1066**	A0UV6456H07	VI.A	phage-related protein
**XAC1068**	A0CE6340D07	VI.A	phage-related tail protein
**XAC1069**	A0UT6817A11	VIII.B	hypothetical protein
**XAC1070**	A0QR6389C01	VI.C	ISxac3 transposase
**XAC1071**	A0CE5318C08	VI.C	ISxac3 transposase
**XAC1074**	A0QR5902H06	I.D.3	sensor histidine kinase
**XAC1075**	A0UV9748F06	I.D.3	sensor histidine kinase
**XAC1077**	A0UV6708B04	III.C.2	peptidyl-prolyl cis-trans isomerase

**XAC1078**	A0JJ1159G08	III.C.3	ATP-dependent Clp protease proteolytic subunit
**XAC1080**	A0UV1533G01	III.C.3	ATP-dependent serine proteinase La
**XAC1081**	A0AC6360H09	III.A.2	histone-like protein
**XAC1085**	A0QH6713A01	III.C.1	peptidyl-prolyl cis-trans isomerase
**XAC1086**	genomic_DNA	VIII.A	conserved hypothetical protein
**XAC1089**	A0UV6710F11	III.B.6	ribonuclease H
**XAC1090**	genomic_DNA	VIII.C	Xanthomonas conserved hypothetical protein
**XAC1091**	genomic_DNA	VIII.C	Xanthomonas conserved hypothetical protein
**XAC1092**	genomic_DNA	VIII.A	conserved hypothetical protein
**XAC1093**	genomic_DNA	I.D.2	conditioned medium factor
**XAC1094**	A0UE9829C03	IV.C	saccharide biosynthesis regulatory protein
**XAC1096**	A0UV6712D12	VIII.A	conserved hypothetical protein
**XAC1097**	A0JE1211G09	II.D.4	molybdenum cofactor biosynthesis protein A
**XAC1099**	A0QR9817A01	II.D.4	molybdopterin-converting factor chain 1
**XAC1101**	A0QR6802H12	III.C.2	heat shock protein G homolog
**XAC1102**	A0QR6366C08	VI.C	ISxac3 transposase
**XAC1103**	A0QR9818G06	VI.C	ISxac3 transposase
**XAC1104**	A0QH6731D06	VI.B	plasmid mobilization protein
**XAC1105**	A0QR6614D04	VIII.B	hypothetical protein
**XAC1106**	A0UV6515D03	VIII.B	hypothetical protein
**XAC1107**	A0UV6739H05	VI.A	integrase
**XAC1109**	A0JE1034B09	III.A.1	DNA polymerase III tau and gamma subunits
**XAC1110**	A0UT9843A02	VIII.A	conserved hypothetical protein
**XAC1111**	A0AC6824F03	III.A.3	recombination protein RecR
**XAC1112**	A0UV9751H04	I.A.2	histidine triad protein homolog (HIT-like protein)
**XAC1113**	A0UV6511H05	IV.A.2	outer membrane protein Slp
**XAC1119**	A0QH9865A09	VIII.C	Xanthomonas conserved hypothetical protein
**XAC1120**	A0UV6819G07	VIII.A	conserved hypothetical protein
**XAC1121**	A0AC6450A07	VIII.A	conserved hypothetical protein
**XAC1122**	A0AC6825B08	III.B.2	50S ribosomal protein L32
**XAC1123**	A0AC6823D11	II.E	beta-ketoacyl-[ACP] synthase III
**XAC1124**	A0QR6706H12	VIII.A	conserved hypothetical protein
**XAC1128**	A0UV6765D02	II.E	acyl carrier protein
**XAC1129**	A0UE6718H07	II.E	3-oxoacyl-[ACP] synthase II
**XAC1132**	A0QR6705C03	III.A.1	DNA polymerase III, delta' subunit
**XAC1133**	A0QR6723A03	IV.D	type IV fimbriae assembly protein
**XAC1136**	A0QR6730H04	I.D.2	propionate catabolism regulatory protein
**XAC1138**	A0QR9825B02	I.A.2	citrate synthase 2
**XAC1140**	A0JJ1003G11	VIII.A	conserved hypothetical protein
**XAC1144**	A0QR6752C06	III.B.5	inosine-uridine preferring nucleoside hydrolase
**XAC1148**	A0UV6819B12	IV.A.1	bifunctional penicillin-binding protein 1C
**XAC1152**	A0UV6711F01	III.C.2	curved DNA binding protein
**XAC1153**	A0UV6351D03	VIII.B	hypothetical protein
**XAC1154**	A0UV6846C09	I.D.2	regulatory protein pilH family
**XAC1157**	A0UV6709D05	VIII.A	conserved hypothetical protein
**XAC1158**	A0UV9905D05	II.B.1	adenylosuccinate synthetase
**XAC1160**	A0CE6742E07	I.C.3	oxidoreductase
**XAC1161**	A0AC6827C09	VIII.A	conserved hypothetical protein
**XAC1163**	A0UV6736E11	VIII.C	Xanthomonas conserved hypothetical protein
**XAC1165**	A0QR6702G06	VIII.C	Xanthomonas conserved hypothetical protein
**XAC1166**	A0RN1548D04	VIII.C	Xanthomonas conserved hypothetical protein
**XAC1168**	A0QR5103F10	VIII.A	conserved hypothetical protein
**XAC1169**	A0CE6507B08	VIII.B	hypothetical protein
**XAC1170**	A0UV6708E04	VIII.B	hypothetical protein

**XAC1171**	A0QH6731G10	I.D.3	serine/threonine kinase
**XAC1173**	A0UV6844E10	I.D.2	transcriptional regulator uid family
**XAC1176**	A0QR6802E02	IV.C	glycosyl hydrolase
**XAC1179**	A0QH6835F09	VIII.A	conserved hypothetical protein
**XAC1180**	A0CE0105B11	I.C.3	oxidoreductase
**XAC1181**	A0UV6485F06	VIII.A	conserved hypothetical protein
**XAC1182**	A0QH6732F07	II.D.10	thioredoxin reductase
**XAC1184**	A0QR9860B10	VIII.A	conserved hypothetical protein
**XAC1185**	A0JJ1118F10	VIII.A	conserved hypothetical protein
**XAC1190**	A0UV5525G03	VIII.A	conserved hypothetical protein
**XAC1191**	A0UV5524D12	VIII.C	Xanthomonas conserved hypothetical protein
**XAC1192**	A0UV6712F09	VII.C	tetracenomycin polyketide synthesis protein
**XAC1193**	A0QR6730H08	VIII.A	conserved hypothetical protein
**XAC1195**	A0QR6614E08	VIII.B	hypothetical protein
**XAC1196**	A0UE6759H07	I.D.2	LexA repressor
**XAC1199**	A0JE5637B08	III.A.1	DNA polymerase III, alpha chain
**XAC1200**	A0QR6840H04	III.C.3	prolyl oligopeptidase family protein
**XAC1201**	A0UE6718F05	VIII.A	conserved hypothetical protein
**XAC1203**	A0UV6707G02	VIII.C	Xanthomonas conserved hypothetical protein
**XAC1204**	A0RN1195F02	III.C.3	alanyl dipeptidyl peptidase
**XAC1205**	A0UV6764E06	VIII.A	conserved hypothetical protein
**XAC1206**	A0UE9829C11	VIII.C	Xanthomonas conserved hypothetical protein
**XAC1207**	A0UV6733D02	I.D.2	transcriptional regulator araC/xylS family
**XAC1208**	A0QR5903B01	VIII.C	Xanthomonas conserved hypothetical protein
**XAC1209**	A0AC9835A01	VIII.B	hypothetical protein
**XAC1212**	A0UV9870B09	I.C.3	oxidoreductase
**XAC1213**	A0QR5508A07	III.D.2	carboxylesterase, type B
**XAC1214**	A0UV1356H05	I.A.2	glycine decarboxylase
**XAC1216**	A0UV6734G05	VIII.C	Xanthomonas conserved hypothetical protein
**XAC1217**	A0QR5530C03	III.C.3	dipeptidyl carboxypeptidase
**XAC1220**	A0UV6485H09	VIII.A	conserved hypothetical protein
**XAC1224**	A0UV6765C04	V.B	cell division topological specificity factor
**XAC1226**	A0UV6736C11	V.B	cell division inhibitor
**XAC1228**	A0QR5704C05	I.D.1	two-component system, sensor protein
**XAC1230**	A0UV5307D10	VIII.A	conserved hypothetical protein
**XAC1231**	A0EC1095H07	VIII.A	conserved hypothetical protein
**XAC1234**	A0CE0105C09	VIII.A	conserved hypothetical protein
**XAC1235**	A0UV6712H03	VIII.C	Xanthomonas conserved hypothetical protein
**XAC1237**	A0AC6824C12	II.B.1	phosphoribosylglycinamide formyltransferase 2
**XAC1240**	A0JJ1004A12	VIII.A	conserved hypothetical protein
**XAC1242**	A0UV6820C12	VII.H	pathogenicity-related protein
**XAC1243**	A0JE1208E03	III.D.2	acyl-CoA thiolesterase II
**XAC1245**	A0CE6828B09	VIII.A	conserved hypothetical protein
**XAC1246**	A0UV6710H02	VIII.A	conserved hypothetical protein
**XAC1250**	A0JJ1421C01	IX	GTP-binding protein
**XAC1251**	A0UV6818F06	III.B.2	30S ribosomal protein S20
**XAC1252**	A0QR6749A05	VII.H	virulence factor
**XAC1254**	A0QR9717B01	III.B.4	isoleucyl-tRNA synthetase
**XAC1255**	A0JJ1101F08	III.C.1	lipoprotein signal peptidase
**XAC1256**	A0UV6210A11	VII.C	penicillin tolerance protein
**XAC1258**	A0QR9756A02	I.C.1	cytochrome O ubiquinol oxidase, subunit II
**XAC1259**	A0UV6819F02	I.C.1	cytochrome O ubiquinol oxidase, subunit I
**XAC1260**	A0CE1623F05	I.C.1	cytochrome O ubiquinol oxidase, subunit III
**XAC1263**	A0UV6845D04	III.A.4	DNA repair protein

**XAC1264**	A0AC6827F11	VIII.A	conserved hypothetical protein
**XAC1265**	genomic_DNA	VII.B	HrpG protein
**XAC1266**	genomic_DNA	VII.B	HrpX protein
**XAC1266**	A0UV6711A02	VII.B	HrpX protein
**XAC1267**	A0AC6361A07	VII.G	Hsp90xo protein
**XAC1268**	A0AM1111G06	VIII.A	conserved hypothetical protein
**XAC1269**	A0QR6725A03	I.D.2	positive regulator of sigma-B
**XAC1269**	genomic_DNA	I.D.2	positive regulator of sigma-B
**XAC1270**	A0UV6820E10	I.D.2	negative regulator of sigma-B
**XAC1271**	genomic_DNA	I.D.2	sigma-B negative effector
**XAC1271**	A0QR5704A09	I.D.2	sigma-B negative effector
**XAC1275**	A0QH6811A01	I.A.1	xylosidase/arabinosidase
**XAC1276**	A0JJ1536G05	V.A.7	TonB-dependent receptor
**XAC1279**	A0AM1363E05	I.D.1	two-component system, regulatory protein
**XAC1281**	A0AC0115E12	V.C	chemotaxis protein
**XAC1282**	A0EC1088H01	I.D.1	two-component system, sensor protein
**XAC1282**	genomic_DNA	I.D.1	two-component system, sensor protein
**XAC1283**	A0CE9842E10	I.D.1	two-component system, sensor protein
**XAC1284**	A0UE6759G02	I.D.1	two-component system, regulatory protein
**XAC1286**	A0AC6496B06	I.A.1	alpha-L-arabinosidase
**XAC1287**	A0UV9819G02	I.B.10	aldose 1-epimerase
**XAC1289**	A0JE1333C11	V.A.6	signal recognition particle protein
**XAC1292**	A0QR9705B04	III.B.2	30S ribosomal protein S16
**XAC1295**	A0AC6827B11	III.B.2	50S ribosomal protein L19
**XAC1296**	A0QR9732G07	VIII.A	conserved hypothetical protein
**XAC1297**	A0AC0113B03	VIII.A	conserved hypothetical protein
**XAC1298**	A0CE9839F02	I.D.2	transcriptional regulator
**XAC1299**	A0JE1636A02	VII.C	glutathione S-transferase
**XAC1301**	A0QR6752A06	VII.C	catalase/peroxidase
**XAC1302**	A0CE6740E03	VIII.A	conserved hypothetical protein
**XAC1303**	A0UV6821E02	III.A.4	DNA mismatch repair protein
**XAC1304**	A0QR6702B05	VIII.B	hypothetical protein
**XAC1305**	A0UV6734G09	IV.A.2	wall-associated protein
**XAC1306**	A0QR9739F08	VIII.A	conserved hypothetical protein
**XAC1307**	A0QR6730F07	VIII.C	Xanthomonas conserved hypothetical protein
**XAC1310**	A0UT6817F08	V.A.7	TonB-dependent receptor
**XAC1311**	A0UV6845F08	I.D.2	transcriptional regulator
**XAC1313**	A0JJ1537D09	I.A.3	acyl-CoA dehydrogenase
**XAC1314**	A0UV9819A08	I.A.3	enoyl-CoA hydratase
**XAC1315**	A0UE9886D10	I.A.3	enoyl-CoA hydratase
**XAC1316**	A0UT6815F10	I.A.2	3-hydroxyisobutirate dehydrogenase
**XAC1319**	A0UT6815A06	I.D.4	RNA polymerase sigma-H factor
**XAC1321**	A0EC6316B12	III.C.3	periplasmic protease
**XAC1323**	A0QH6326C11	III.C.1	signal peptidase I
**XAC1324**	A0CE6345G11	VIII.A	conserved hypothetical protein
**XAC1327**	A0UV9901C06	III.A.3	DNA repair protein
**XAC1328**	A0JJ1626B12	VIII.A	conserved hypothetical protein
**XAC1329**	A0QR5902A01	III.B.3	RNA methyltransferase
**XAC1330**	A0UT6815B05	VIII.A	conserved hypothetical protein
**XAC1333**	A0QR5904A09	VIII.A	conserved hypothetical protein
**XAC1334**	A0AC6429D09	I.B.1	N-acetyl-beta-glucosaminidase
**XAC1335**	A0JE5637D08	II.B.4	hypoxanthine-guanine phosphoribosyltransferase
**XAC1336**	A0UV9881G05	II.B.4	purine nucleoside phosphorylase
**XAC1337**	A0UV6844G05	VII.G	cold shock protein

**XAC1339**	A0QH6713E05	VIII.A	conserved hypothetical protein
**XAC1340**	A0QR6490F04	III.A.1	helicase-related protein
**XAC1342**	A0UT9894B02	III.B.5	mRNA 3'-end processing factor
**XAC1344**	A0UV9746D08	VIII.A	conserved hypothetical protein
**XAC1345**	A0QR6802E09	I.D.3	sensor histidine kinase
**XAC1347**	A0QR9815A07	IV.A.2	outer membrane protein
**XAC1349**	A0QR5106A11	III.C.3	serine protease
**XAC1350**	A0QH6412H04	I.A.2	leucine dehydrogenase
**XAC1351**	A0UV6821B05	VIII.A	conserved hypothetical protein
**XAC1352**	A0QR6728B07	VIII.A	conserved hypothetical protein
**XAC1353**	A0CE9840H04	VIII.A	conserved hypothetical protein
**XAC1354**	A0QR9815E05	VIII.C	Xanthomonas conserved hypothetical protein
**XAC1355**	A0UT9894C02	VIII.A	conserved hypothetical protein
**XAC1357**	A0QR5904D08	VII.G	heat shock protein
**XAC1360**	A0AC6827F03	VIII.A	conserved hypothetical protein
**XAC1363**	A0AC1314F05	V.A.7	MFS transporter
**XAC1364**	A0QH6731A12	VIII.C	Xanthomonas conserved hypothetical protein
**XAC1369**	A0UV6363F10	VIII.A	conserved hypothetical protein
**XAC1370**	A0QR6801B04	VIII.C	Xanthomonas conserved hypothetical protein
**XAC1372**	A0UV6708F07	VIII.A	conserved hypothetical protein
**XAC1374**	A0UV9880E06	VIII.C	Xanthomonas conserved hypothetical protein
**XAC1376**	A0QR9759D12	VIII.A	conserved hypothetical protein
**XAC1377**	A0CE0105A02	I.C.3	dehydrogenase
**XAC1378**	A0UV6711G12	III.D.2	delta 9 acyl-lipid fatty acid desaturase
**XAC1379**	A0QR5407F06	VIII.A	conserved hypothetical protein
**XAC1380**	genomic_DNA	I.D.4	RNA polymerase sigma factor
**XAC1380**	A0UV9747D03	I.D.4	RNA polymerase sigma factor
**XAC1381**	A0AC6824G06	VIII.C	Xanthomonas conserved hypothetical protein
**XAC1382**	A0QH6313B06	VIII.C	Xanthomonas conserved hypothetical protein
**XAC1384**	A0UV9879B07	I.C.3	ferredoxin II
**XAC1385**	A0UV6736F02	V.B	conserved hypothetical protein
**XAC1386**	A0JE1211H05	III.B.4	methionyl-tRNA synthetase
**XAC1387**	A0QH6309D01	VIII.A	conserved hypothetical protein
**XAC1391**	A0AC6405C02	V.A.1	S-methylmethionine permease
**XAC1393**	A0UV5502C11	VIII.A	conserved hypothetical protein
**XAC1394**	A0AC6440G07	VIII.A	conserved hypothetical protein
**XAC1396**	A0CE6341D04	VIII.C	Xanthomonas conserved hypothetical protein
**XAC1399**	A0QR6840G03	IX	sensor protein
**XAC1401**	A0CE9839E07	VIII.A	conserved hypothetical protein
**XAC1402**	A0AC6827B01	VIII.A	conserved hypothetical protein
**XAC1403**	A0UV6818F10	VIII.B	hypothetical protein
**XAC1404**	A0UV6458E06	VIII.A	conserved hypothetical protein
**XAC1405**	A0UV6818H05	II.E	acetyl-coenzyme A carboxylase carboxyl transferase
**XAC1408**	A0QR6393B10	IV.C	lipid A disaccharide synthase
**XAC1409**	A0QH6731G05	IV.C	UDP-N-acetylglucosamine acyltransferase
**XAC1412**	A0CE6480A04	VIII.C	Xanthomonas conserved hypothetical protein
**XAC1413**	A0AC6446H11	IV.A.2	outer membrane antigen
**XAC1418**	A0QR6749H10	III.C.1	ribosome recycling factor
**XAC1419**	A0QH6382C03	I.B.8	uridylate kinase
**XAC1423**	A0UV6846C06	III.C.2	pili assembly chaperone
**XAC1425**	A0UV6764F12	IV.A.2	outer membrane usher protein FasD
**XAC1426**	A0UV6845E04	III.C.2	pili assembly chaperone
**XAC1427**	A0UV6350F11	VII.F	protein U
**XAC1428**	A0UV6761A04	III.C.1	methionine aminopeptidase

**XAC1431**	A0UT6211B06	VIII.A	conserved hypothetical protein
**XAC1432**	A0UV6710E06	II.A.2	succinyl-diaminopimelate desuccinylase
**XAC1433**	A0UV6843F01	II.A.2	asparagine synthase B
**XAC1434**	A0AC6406B12	VIII.A	conserved hypothetical protein
**XAC1435**	A0UE6610E07	V.A.7	iron receptor
**XAC1437**	A0UT9878G03	VII.C	penicillin acylase II
**XAC1438**	A0AC6825B03	V.A.4	bacterioferritin
**XAC1439**	A0QR5205H12	VII.C	thiopurine methyltransferase
**XAC1445**	A0UV6820H09	VII.C	multidrug resistance efflux pump
**XAC1448**	A0AM1402G03	I.A.1	beta-glucosidase
**XAC1449**	A0QR5902G10	VIII.A	conserved hypothetical protein
**XAC1451**	A0UV6734A09	VIII.A	conserved hypothetical protein
**XAC1452**	A0CE6829F10	VIII.B	hypothetical protein
**XAC1454**	A0QH6306H08	VIII.C	Xanthomonas conserved hypothetical protein
**XAC1456**	A0QH9865D07	III.C.3	peptidyl-dipeptidase
**XAC1457**	A0UV6488G11	VII.C	glutathione peroxidase-like protein
**XAC1458**	A0JE1142A02	I.C.3	ferredoxin-NADP reductase
**XAC1459**	A0QH6732C10	V.A.7	ABC transporter ATP-binding protein
**XAC1461**	A0UV9880H08	VII.C	glutathione S-transferase
**XAC1463**	A0UV6708D05	III.D.2	phospholipase
**XAC1464**	A0UV6845G02	VIII.A	conserved hypothetical protein
**XAC1465**	A0UV6733H03	VII.G	major cold shock protein
**XAC1467**	A0CE6742E05	II.D.4	molybdopterin biosynthesis protein
**XAC1469**	A0QR6001D02	VIII.A	conserved hypothetical protein
**XAC1470**	A0UV6712C04	VIII.C	Xanthomonas conserved hypothetical protein
**XAC1472**	A0UV6210H10	I.A.2	glutaryl-CoA dehydrogenase
**XAC1474**	A0UV6733E09	VII.C	glutathione S-transferase
**XAC1477**	A0JE1044A10	III.A.1	replicative DNA helicase
**XAC1479**	A0UV6819D05	IV.A.2	OmpA family protein
**XAC1480**	A0UE6759F11	I.D.2	transcriptional regulator
**XAC1481**	A0QR6730E06	I.C.3	dehydrogenase
**XAC1482**	A0UV6711D08	VII.C	RND multidrug efflux membrane fusion protein
**XAC1483**	A0QR6755E05	VII.C	RND multidrug efflux transporter MexF
**XAC1484**	A0AC6824C10	I.C.3	short chain dehydrogenase
**XAC1487**	A0QR6390F12	VIII.C	Xanthomonas conserved hypothetical protein
**XAC1489**	A0QR6725H12	VIII.C	Xanthomonas conserved hypothetical protein
**XAC1490**	A0CE6480B05	VIII.A	conserved hypothetical protein
**XAC1492**	A0UV6820C01	VIII.A	conserved hypothetical protein
**XAC1494**	A0AC6827G03	VI.A	phage-related protein
**XAC1495**	genomic_DNA	VII.H	virulence regulator
**XAC1498**	A0QH6836E07	VI.A	integrase
**XAC1499**	A0AM1050F07	I.D.2	transcriptional regulator
**XAC1501**	A0QR6729F02	VIII.B	hypothetical protein
**XAC1502**	A0JE1376A05	VIII.B	hypothetical protein
**XAC1503**	A0QH9737C01	VIII.C	Xanthomonas conserved hypothetical protein
**XAC1504**	A0QR5101G05	VI.C	ISxcd1 transposase
**XAC1506**	A0QR6704E02	VIII.A	conserved hypothetical protein
**XAC1507**	A0QR6768G03	VI.B	plasmid mobilization protein
**XAC1509**	A0UT9742C09	VIII.A	conserved hypothetical protein
**XAC1510**	A0QH6835H03	VI.A	phage-related integrase
**XAC1511**	A0UV9901D05	III.B.1	tmRNA
**XAC1512**	A0UV6487C09	III.C.3	serine peptidase
**XAC1517**	A0UE6759C05	I.D.2	ferric uptake regulator
**XAC1518**	A0EC6316C07	VIII.A	conserved hypothetical protein

**XAC1519**	A0QH6307G12	III.A.3	recombination protein N
**XAC1521**	A0UV9900F05	III.C.2	heat shock protein GrpE
**XAC1523**	A0UV6434C01	III.C.2	DnaJ protein
**XAC1524**	A0JJ1004G06	II.D.6	pyridoxine kinase
**XAC1525**	A0QR5206B07	II.A.4	chorismate mutase/prephenate dehydrogenase
**XAC1526**	A0QR6460G08	VII.C	outer membrane component of multidrug efflux pump
**XAC1527**	A0UV6485H08	VII.C	RND efflux membrane fusion protein
**XAC1529**	A0CE6746D03	I.D.2	hydrogen peroxide-inducible genes activator
**XAC1532**	A0EC6474F07	VIII.A	conserved hypothetical protein
**XAC1534**	A0UV6762G05	I.C.7	dihydrolipoamide S-succinyltransferase
**XAC1538**	A0UV6511H09	VIII.C	Xanthomonas conserved hypothetical protein
**XAC1540**	A0CE6742H08	VIII.B	hypothetical protein
**XAC1542**	A0QH9769G01	I.C.7	fumarate hydratase
**XAC1543**	A0UE9886B12	VIII.A	conserved hypothetical protein
**XAC1544**	A0QR6334C07	VIII.C	Xanthomonas conserved hypothetical protein
**XAC1549**	A0AC6823B06	V.A.7	ABC transporter vitamin B12 uptakepermease
**XAC1550**	A0QR6728G09	III.C.1	FKBP-type peptidyl-prolyl cis-trans isomerase (rotamase)
**XAC1551**	A0UV6765G04	I.B.11	UDP-glucose dehydrogenase
**XAC1552**	A0QR6725B03	VIII.A	conserved hypothetical protein
**XAC1553**	A0AC9896B08	VIII.A	conserved hypothetical protein
**XAC1554**	A0QR5407H09	VIII.C	Xanthomonas conserved hypothetical protein
**XAC1555**	A0UV1358B10	I.D.2	transcriptional regulator
**XAC1556**	A0UT9877E01	V.A.3	glucose/galactose transporter
**XAC1557**	A0UV9750G10	I.A.2	fructokinase
**XAC1558**	A0UV6733G09	VIII.A	conserved hypothetical protein
**XAC1560**	A0UV6710B08	II.A.2	5-methyltetrahydrofolate-homocysteine methyl transferase
**XAC1561**	A0UT9876G07	I.D.2	transcriptional regulator
**XAC1568**	genomic_DNA	VIII.A	conserved hypothetical protein
**XAC1568**	A0QR5702D09	VIII.A	conserved hypothetical protein
**XAC1571**	A0UT6814E08	III.B.6	ribonuclease T
**XAC1572**	A0UV6733C03	VIII.B	hypothetical protein
**XAC1573**	A0QR6369A06	I.D.2	phosphate regulon transcriptional regulator
**XAC1577**	A0QR6723A07	V.A.2	ABC transporter phosphate binding
**XAC1579**	A0UV6736D10	IV.A.2	polyphosphate-selective porin O
**XAC1580**	A0QR5904E06	I.B.10	carbonic anhydrase
**XAC1586**	A0AR0913E08	III.A.4	MutT/nudix family protein
**XAC1587**	A0QR6728A05	I.B.12	thiosulfate sulfurtransferase
**XAC1588**	A0UV6351A05	VIII.A	conserved hypothetical protein
**XAC1591**	A0UT6817E06	VIII.A	conserved hypothetical protein
**XAC1594**	A0QR6804B03	VIII.A	conserved hypothetical protein
**XAC1596**	A0AC0115H07	II.B.4	5'-nucleotidase
**XAC1597**	A0UV6733D09	VIII.A	conserved hypothetical protein
**XAC1599**	A0QR9705F04	III.A.4	exodeoxyribonuclease I
**XAC1601**	A0UV6820E11	II.D.17	kynureninase
**XAC1602**	A0CE6830E02	VIII.A	conserved hypothetical protein
**XAC1605**	A0QR6704B07	VIII.A	conserved hypothetical protein
**XAC1606**	A0UV6734G02	VIII.B	hypothetical protein
**XAC1607**	A0UV6736D09	VIII.A	conserved hypothetical protein
**XAC1608**	A0UV6710G08	VIII.B	hypothetical protein
**XAC1609**	A0QH6835H02	VIII.B	hypothetical protein
**XAC1611**	A0UV6739F02	VIII.B	hypothetical protein
**XAC1613**	A0UV6821E01	VIII.B	hypothetical protein
**XAC1615**	A0JJ1541D02	I.D.2	transcriptional regulator protein Pai2
**XAC1616**	A0AM1540D02	VIII.C	Xanthomonas conserved hypothetical protein

**XAC1617**	A0QR6614F04	VIII.C	Xanthomonas conserved hypothetical protein
**XAC1618**	A0QR6482E05	III.B.4	asparaginyl-tRNA synthetase
**XAC1620**	A0AM1424A11	III.B.2	30S ribosomal protein S6
**XAC1621**	A0JJ1439G07	III.B.2	30S ribosomal protein S18
**XAC1627**	A0AC1335D07	III.A.1	DNA ligase
**XAC1629**	A0UV6733C04	VIII.A	conserved hypothetical protein
**XAC1631**	A0QR6729G01	III.A.1	DNA gyrase subunit A
**XAC1632**	A0UV6736D02	VIII.B	hypothetical protein
**XAC1633**	A0JJ1131A01	I.C.5	glucose dehydrogenase
**XAC1636**	A0JJ0102G11	I.A.2	formylglutamate amidohydrolase
**XAC1638**	A0AC0113H02	I.A.2	imidazolonepropionase
**XAC1639**	A0AM1452B11	I.A.2	atrazine chlorohydrolase
**XAC1644**	A0QR5904F07	VIII.A	conserved hypothetical protein
**XAC1648**	A0UT5315A07	II.A.3	phosphoserine aminotransferase
**XAC1649**	A0QR6729H05	II.A.4	P-protein
**XAC1653**	A0JJ1166F08	III.B.4	seryl-tRNA synthetase
**XAC1655**	A0AC0113G09	I.D.2	transcriptional regulator
**XAC1657**	A0CE6743D06	VIII.B	hypothetical protein
**XAC1658**	A0CE6740B08	VIII.B	hypothetical protein
**XAC1660**	A0UT9846G09	VI.C	ISxac3 transposase
**XAC1662**	A0UV6843G12	VI.A	phage-related protein
**XAC1663**	A0JJ1437G11	VIII.B	hypothetical protein
**XAC1664**	A0QH6810A05	VI.A	phage-related integrase
**XAC1666**	A0QR6839F02	V.C	chemotaxis protein
**XAC1667**	A0UV6766D08	I.C.3	oxidoreductase
**XAC1668**	A0JJ0101B03	I.D.2	transcriptional regulator for cryptic hemolysin
**XAC1669**	A0QR6409B06	I.D.3	histidine kinase/response regulator hybrid protein
**XAC1673**	A0UE6757F06	VIII.A	conserved hypothetical protein
**XAC1674**	A0JE5637B11	I.C.3	C-type cytochrome biogenesis protein
**XAC1677**	A0AC6429E07	I.C.3	C-type cytochrome biogenesis protein
**XAC1680**	A0RN1628B05	III.C.3	serine protease
**XAC1682**	genomic_DNA	I.D.4	RNA polymerase sigma-E factor
**XAC1684**	A0UV6712F06	I.C.3	cytochrome C2
**XAC1685**	A0JJ1312D09	I.C.3	cytochrome C
**XAC1687**	A0JJ1049E04	VIII.A	conserved hypothetical protein
**XAC1688**	A0UE6759H09	VIII.A	conserved hypothetical protein
**XAC1689**	A0CE6480F01	VIII.A	conserved hypothetical protein
**XAC1691**	A0QR6804H07	IV.B	aminotransferase
**XAC1692**	A0UV6819H06	IV.C	lipopolysaccharide biosynthesis protein
**XAC1693**	A0QR5701A09	IV.C	glycosyl transferase
**XAC1694**	A0AC6825G10	VIII.C	Xanthomonas conserved hypothetical protein
**XAC1695**	A0UV9880F08	VIII.C	Xanthomonas conserved hypothetical protein
**XAC1700**	A0UV6711G04	III.D.1	hexosyltransferase
**XAC1701**	A0JJ1160H11	VIII.C	Xanthomonas conserved hypothetical protein
**XAC1703**	A0QR5902F06	VIII.C	Xanthomonas conserved hypothetical protein
**XAC1705**	A0UV6764H04	V.A.7	MFS transporter
**XAC1706**	A0UV6733A02	VIII.A	conserved hypothetical protein
**XAC1707**	A0QR9739G04	VII.G	general stress protein
**XAC1708**	A0QR5104B11	IV.C	ExoD protein
**XAC1710**	A0UV9750H08	VIII.A	conserved hypothetical protein
**XAC1711**	A0CE5310F06	V.A.7	transport protein
**XAC1712**	A0JJ1167A10	VIII.A	conserved hypothetical protein
**XAC1713**	A0CE6478E03	III.C.3	carboxypeptidase-related protein
**XAC1714**	A0UT6817D06	III.A.1	topoisomerase IV subunit B

**XAC1717**	A0QR5904A01	IV.C	2-dehydro-3-deoxyphosphooctonate aldolase
**XAC1718**	A0QH9865C01	VIII.C	Xanthomonas conserved hypothetical protein
**XAC1719**	A0UV6457A11	I.C.4	enolase
**XAC1720**	A0UE6379E04	VIII.A	conserved hypothetical protein
**XAC1721**	A0QR6730B05	II.E	4-diphosphocytidyl-2C-methyl-D-erythritol synthase
**XAC1722**	A0UV6843A09	II.E	2C-methyl-D-erythritol 2,4-cyclodiphosphate synthase
**XAC1723**	A0AM1113F06	IX	hydrogenase subunit
**XAC1725**	A0JE1377G06	VII.G	survival protein
**XAC1726**	A0JJ0209E09	III.C.1	L-isoaspartate protein carboxylmethyltransferase type II
**XAC1727**	A0UE6757G05	VIII.A	conserved hypothetical protein
**XAC1728**	A0QR5904G08	III.D.3	lipoprotein
**XAC1729**	A0AC6824F10	VIII.A	conserved hypothetical protein
**XAC1730**	A0UV1535A05	VIII.A	conserved hypothetical protein
**XAC1736**	A0JJ1304C12	III.C.3	GTP-binding protein
**XAC1738**	A0EC6474C05	II.D.11	ubiquinone biosynthesis protein
**XAC1739**	A0QH6326B08	I.D.2	LexA repressor
**XAC1740**	A0QR5704A08	III.A.3	RecA protein
**XAC1742**	genomic_DNA	III.B.4	alanyl-tRNA synthetase
**XAC1742**	A0UV9871D12	III.B.4	alanyl-tRNA synthetase
**XAC1743**	A0QR6839D09	I.D.2	carbon storage regulator
**XAC1745**	A0UV6733D08	VIII.B	hypothetical protein
**XAC1746**	A0QR6701H12	V.C	chemotaxis protein
**XAC1747**	A0UE6757E06	VIII.A	conserved hypothetical protein
**XAC1752**	A0UV9884F11	VIII.A	conserved hypothetical protein
**XAC1753**	A0QR6393G02	II.D.8	phosphomethylpyrimidine kinase
**XAC1754**	A0JJ1343C04	VIII.A	conserved hypothetical protein
**XAC1756**	A0QR6805F05	VIII.A	conserved hypothetical protein
**XAC1758**	A0QR9768H06	VIII.C	Xanthomonas conserved hypothetical protein
**XAC1759**	A0QR6749A01	I.D.2	glycine cleavage system transcriptional repressor
**XAC1760**	A0JJ0101B08	II.A.2	dihydroxydipicolinate synthase
**XAC1761**	A0CE6478F04	VIII.A	conserved hypothetical protein
**XAC1765**	A0QH9730E11	I.A.2	4-hydroxy-2-oxoglutarate aldolase/2-deydro-3-deoxyphosphogluconate aldolase
**XAC1766**	A0AC6825D09	I.A.2	4-hydroxy-2-oxoglutarate aldolase/2-deydro-3-deoxyphosphogluconate aldolase
**XAC1767**	A0UV9905E10	I.D.2	galactose-binding protein regulator
**XAC1768**	A0EC6475B01	V.A.7	TonB-dependent receptor
**XAC1770**	A0UV6820B11	VII.D	cellulase
**XAC1771**	A0QR6346E11	I.A.2	sialic acid-specific 9-O-acetylesterase
**XAC1772**	A0UV6708A12	VIII.A	conserved hypothetical protein
**XAC1773**	A0AM1161C01	I.A.1	alpha-xylosidase
**XAC1775**	A0QR6730B07	I.A.2	D-xylulokinase
**XAC1776**	A0UT9891B09	I.A.2	xylose isomerase
**XAC1779**	A0UV6734B07	VIII.C	Xanthomonas conserved hypothetical protein
**XAC1780**	A0UV6485C08	III.D.1	N-acetylmuramoyl-L-alanine amidase
**XAC1783**	A0QH6836C12	III.B.5	polynucleotide adenyltransferase
**XAC1785**	A0RN1306A09	II.D.5	3-methyl-2-oxobutanoate hydroxymethyltransferase
**XAC1786**	A0CE6746F05	II.D.5	pantoate-beta-alanine ligase
**XAC1787**	A0QR5324A02	II.D.5	aspartate 1-decarboxylase precursor
**XAC1788**	A0UV9751C06	I.C.4	glucose-6-phosphate isomerase
**XAC1790**	A0UV6351D08	VIII.A	conserved hypothetical protein
**XAC1792**	A0QR6704E10	I.A.2	alkaline phosphatase
**XAC1793**	A0UT6816C03	VII.D	glucan 1,4-beta-glucosidase
**XAC1796**	A0UV5523H05	I.A.1	mannan endo-1,4-beta-mannosidase
**XAC1800**	A0QR6805C04	III.D.2	phosphatidylglycerophosphatase B-related protein
**XAC1801**	A0QR6705C01	V.A.7	Prop transport protein

**XAC1803**	A0CE6740F05	IV.A.1	integral membrane protein
**XAC1806**	A0UV9750G03	VIII.A	conserved hypothetical protein
**XAC1807**	A0UV6485F05	VIII.A	conserved hypothetical protein
**XAC1808**	A0AC6823G01	I.C.3	aldehyde dehydrogenase
**XAC1812**	A0UV6845E06	IX	HmsF protein
**XAC1813**	A0UV6351H03	IX	HmsH protein
**XAC1814**	A0UV6739D02	VII.C	outer membrane hemolysin activator protein
**XAC1817**	A0QR6803D12	VIII.B	hypothetical protein
**XAC1818**	A0QR5704F05	VII.F	hemagglutinin/hemolysin-related protein
**XAC1819**	A0QR6805C10	I.D.2	tryptophan-rich sensory protein
**XAC1820**	A0QR6801A09	II.A.2	bifunctional aspartokinase/homoserine dehydrogenase I
**XAC1822**	A0UV6818B09	VIII.A	conserved hypothetical protein
**XAC1824**	A0CE6829B06	VIII.A	conserved hypothetical protein
**XAC1825**	A0JE1211G05	I.D.2	fumarate and nitrate reduction regulatory protein
**XAC1826**	A0QR6730G09	III.B.4	histidyl-tRNA synthetase
**XAC1829**	A0JE5637G06	II.A.5	histidinol dehydrogenase
**XAC1830**	A0QR5904F10	II.A.5	histidinol-phosphate aminotransferase
**XAC1831**	A0UV6845F04	II.A.5	imidazoleglycerolphosphate dehydratase/histidinol-phosphate phosphatase bifunctional enzyme
**XAC1832**	A0UT9878C05	II.A.5	amidotransferase
**XAC1833**	A0UV6739F05	II.A.5	phosphoribosylformimino-5- aminoimidazole carboxam
**XAC1836**	A0UT9875C05	VIII.C	Xanthomonas conserved hypothetical protein
**XAC1841**	A0QR6330F10	V.A.1	cationic amino acid transporter
**XAC1842**	A0CE9840B04	V.A.1	cationic amino acid transporter
**XAC1843**	A0CE9842A09	VIII.A	conserved hypothetical protein
**XAC1844**	A0UV6738G09	II.A.3	D-3-phosphoglycerate dehydrogenase
**XAC1847**	A0UV6765H07	VIII.A	conserved hypothetical protein
**XAC1849**	A0CE6743H09	III.C.1	elongation factor P
**XAC1850**	A0QR6729F01	I.A.3	3-hydroxyacyl-CoA dehydrogenase type II
**XAC1851**	A0JE1211F08	I.A.2	hydroxymethylglutaryl-CoA lyase
**XAC1853**	A0UE6718F04	I.A.3	enoyl-CoA hydratase
**XAC1854**	A0UE6718F03	V.A.4	ferrous iron transport protein
**XAC1856**	A0QR6002C04	VIII.A	conserved hypothetical protein
**XAC1857**	A0UV6736H03	VIII.C	Xanthomonas conserved hypothetical protein
**XAC1859**	A0UV5526G05	VIII.A	conserved hypothetical protein
**XAC1860**	A0QH6836F09	II.A.2	dihydrodipicolinate reductase
**XAC1861**	A0CE6740C06	II.B.2	carbamoyl-phosphate synthase small chain
**XAC1863**	A0UV6765E06	III.B.5	transcriptional elongation factor
**XAC1864**	A0UT6499C08	VII.H	regulatory protein
**XAC1864**	genomic_DNA	VII.H	regulatory protein
**XAC1866**	A0CE6740E01	IV.C	wall associated protein
**XAC1867**	A0QR6106H05	VIII.A	conserved hypothetical protein
**XAC1869**	A0UV6821B07	VIII.B	hypothetical protein
**XAC1870**	A0UV6350G04	VIII.B	hypothetical protein
**XAC1872**	A0CE5420G07	VI.C	transposase
**XAC1873**	A0UV6712B08	IV.A.1	inner membrane protein
**XAC1874**	genomic_DNA	VII.H	response regulator
**XAC1875**	A0UV6514H09	III.C.1	peptide chain release factor 2
**XAC1876**	A0JJ1434B09	III.B.4	lysyl-tRNA synthetase heat inducible
**XAC1877**	A0UE6398E11	VII.H	response regulator
**XAC1877**	genomic_DNA	VII.H	response regulator
**XAC1878**	A0QR9705E09	VII.H	RpfC protein
**XAC1878**	genomic_DNA	VII.H	RpfC protein
**XAC1879**	A0QH6713H04	VII.H	RpfF protein

**XAC1879**	genomic_DNA	VII.H	RpfF protein
**XAC1880**	genomic_DNA	VII.H	RpfB protein
**XAC1881**	A0UV6733C07	VIII.C	Xanthomonas conserved hypothetical protein
**XAC1882**	A0AM1427B07	VII.H	aconitase
**XAC1882**	genomic_DNA	VII.H	aconitase
**XAC1883**	A0QR5407A09	VIII.A	conserved hypothetical protein
**XAC1884**	A0QR6723A08	VIII.A	conserved hypothetical protein
**XAC1885**	A0UV6488D09	I.C.7	aconitate hydratase 2
**XAC1887**	A0EC6322B04	III.D.1	c-di-GMP phosphodiesterase A
**XAC1889**	A0AC1317A07	V.C	chemotaxis protein
**XAC1891**	A0UV9905A05	V.C	chemotaxis protein
**XAC1893**	A0QH6732B10	V.C	chemotaxis protein
**XAC1894**	A0UV9804A04	V.C	chemotaxis protein
**XAC1897**	A0QR5902F04	V.C	chemotaxis protein
**XAC1898**	A0QR6418C10	VIII.B	hypothetical protein
**XAC1899**	A0UT9891E08	V.C	chemotaxis protein
**XAC1900**	A0AC6407C10	V.C	chemotaxis protein
**XAC1901**	A0JE5637A11	VIII.C	Xanthomonas conserved hypothetical protein
**XAC1906**	A0UT9800D01	V.C	chemotaxis protein
**XAC1911**	A0QH6811B11	VIII.B	hypothetical protein
**XAC1912**	A0UV6711B07	I.D.3	serine/threonine kinase
**XAC1914**	A0UV6711G06	VIII.C	Xanthomonas conserved hypothetical protein
**XAC1915**	A0QR6725C09	VIII.C	Xanthomonas conserved hypothetical protein
**XAC1916**	A0UE9889E10	VI.C	ISxac1 transposase
**XAC1918**	A0CE6740G08	VII.C	hemolysin related protein
**XAC1919**	A0UV6761B10	VIII.B	hypothetical protein
**XAC1921**	A0UV9903A04	VI.C	ISxac3 transposase
**XAC1922**	A0UV6515G01	VIII.B	hypothetical protein
**XAC1923**	A0QH6732G03	VIII.B	hypothetical protein
**XAC1924**	A0UE6465C06	VI.C	transposase
**XAC1925**	A0AM1363D10	I.D.2	transcriptional regulator
**XAC1928**	A0UE6718E11	VIII.B	hypothetical protein
**XAC1929**	A0QR6304F02	VI.C	ISxac1 transposase
**XAC1932**	A0CE6831C02	V.C	chemotaxis protein
**XAC1934**	A0UV6709G07	V.B	flagellar biosynthesis switch protein
**XAC1936**	A0UV5307E02	V.C	flagellar protein
**XAC1938**	A0UV6736A05	I.D.4	GGDEF family protein
**XAC1939**	A0JJ1165H10	I.D.4	GGDEF family protein
**XAC1941**	A0UV6735E09	V.C	flagellar biosynthetic protein
**XAC1942**	A0QR6304H09	V.C	flagellar biosynthesis
**XAC1944**	A0JE1041F05	V.C	flagellar biosynthetic protein
**XAC1945**	A0RN1498A05	V.C	flagellar protein
**XAC1946**	A0EC6321E04	V.C	flagellar protein
**XAC1948**	A0QH6308F02	V.C	flagellar protein
**XAC1949**	A0AC6359E01	V.C	flagellar protein
**XAC1950**	A0EC6321H02	V.C	flagellar FliJ protein
**XAC1951**	A0EC6322B02	V.C	flagellar protein
**XAC1953**	A0JJ0101D08	V.C	flagellar protein
**XAC1954**	A0AC6822C03	V.C	flagellar protein
**XAC1955**	A0UV9750F07	V.C	flagellar protein
**XAC1956**	A0CE6743C06	VIII.A	conserved hypothetical protein
**XAC1957**	A0UV1650G05	IV.C	O-antigen biosynthesis protein
**XAC1958**	A0AM1402E11	VIII.A	conserved hypothetical protein
**XAC1959**	A0QR6801D12	VIII.A	conserved hypothetical protein

**XAC1963**	A0QR6730C02	II.E	3-oxoacyl-[ACP] reductase
**XAC1964**	A0UT6814G01	II.E	3-oxoacyl-[ACP] synthase
**XAC1966**	A0QR6417F05	IV.C	nucleotide sugar transaminase
**XAC1968**	A0AM1594G07	IX	response regulator
**XAC1969**	A0QR6106E06	I.D.4	RNA polymerase sigma-54 factor
**XAC1970**	A0AC6359C10	IX	response regulator
**XAC1971**	A0UV6351C12	VIII.C	Xanthomonas conserved hypothetical protein
**XAC1972**	A0CE9728H09	VIII.C	Xanthomonas conserved hypothetical protein
**XAC1973**	A0QR6366G09	V.C	flagellar protein
**XAC1974**	A0QR5904G07	V.C	flagellar protein
**XAC1975**	A0QH6810E06	V.C	flagellar protein
**XAC1977**	A0QH6326A08	V.C	flagellar protein
**XAC1979**	A0QR5206F07	V.C	flagellar protein
**XAC1980**	A0QR6389C07	V.C	flagellar L-ring protein
**XAC1983**	A0UV6711B02	V.C	flagellar biosynthesis, hook protein
**XAC1985**	A0QR6392D03	V.C	flagellar biosynthesis, cell-proximal portion of basal-body rod
**XAC1988**	A0QR9757C01	V.C	flagellar protein
**XAC1989**	genomic_DNA	V.C	flagellar protein
**XAC1990**	A0AC6405G08	VIII.C	Xanthomonas conserved hypothetical protein
**XAC1991**	A0QR9859D11	I.D.3	histidine kinase
**XAC1994**	genomic_DNA	VII.B	HrpX related protein
**XAC1996**	A0UE6612E05	V.C	chemotaxis protein
**XAC1997**	A0AC0115B12	VIII.A	conserved hypothetical protein
**XAC1998**	A0UV9822A04	III.B.4	tRNA methyltransferase
**XAC2000**	A0QH6811D05	VIII.A	conserved hypothetical protein
**XAC2001**	A0JJ1169A07	III.C.3	ATP-dependent Clp protease subunit
**XAC2004**	A0UV6512A06	VIII.A	conserved hypothetical protein
**XAC2005**	A0QR5206F01	II.B.3	thioredoxin reductase
**XAC2007**	A0QR6725E09	VIII.A	conserved hypothetical protein
**XAC2008**	A0AC0115H01	III.C.2	outer-membrane lipoproteins carrier protein precursor
**XAC2009**	A0UV6739D01	VIII.A	conserved hypothetical protein
**XAC2011**	A0QR5704A11	VIII.A	conserved hypothetical protein
**XAC2012**	A0UT9876A10	I.A.3	3-ketoacyl-CoA thiolase
**XAC2013**	A0AC6405C01	I.A.3	3-hydroxyacyl-CoA dehydrogenase
**XAC2017**	A0QR5903G03	IV.D	fimbrial biogenesis protein
**XAC2018**	A0QR6729F03	VIII.A	conserved hypothetical protein
**XAC2019**	A0QR5205E09	VIII.A	conserved hypothetical protein
**XAC2021**	A0RN1634F09	IX	GTP-binding protein
**XAC2022**	A0UV6363H11	II.D.4	molybdopterin biosynthesis
**XAC2023**	A0QR6749B11	II.D.4	molybdopterin biosynthesis protein
**XAC2024**	A0UT6386F04	V.A.7	TonB-dependent receptor
**XAC2025**	A0QR6749G11	VIII.C	Xanthomonas conserved hypothetical protein
**XAC2026**	A0UV5503C08	VIII.A	conserved hypothetical protein
**XAC2027**	A0UT9843F09	VIII.C	Xanthomonas conserved hypothetical protein
**XAC2030**	A0QR6768A07	III.A.4	exodeoxyribonuclease III
**XAC2033**	A0UV6710A10	II.D.4	molybdopterin guanine dinucleotide synthase
**XAC2035**	A0UT6816E09	I.C.3	non-heme chloroperoxidase
**XAC2041**	A0AC1317A11	I.B.3	phosphoenolpyruvate synthase
**XAC2044**	A0UV5307A02	III.A.4	7,8-dihydro-8-oxoguanine-triphosphatase
**XAC2045**	A0UV6739B01	I.C.3	D-beta-hydroxybutyrate dehydrogenase
**XAC2046**	A0UV6712C03	III.D.2	CDP-diacylglycerol--serine o-phospase, cytochrome C1 subunit
**XAC2047**	A0UV6738H03	IX	PHA synthase subunit
**XAC2047**	genomic_DNA	IX	PHA synthase subunit
**XAC2047**	genomic_DNA	IX	PHA synthase subunit

**XAC2048**	A0JE1039A04	II.E	poly (3-hydroxybutyric acid) synthase
**XAC2050**	A0UV6709H07	VIII.C	Xanthomonas conserved hypothetical protein
**XAC2051**	A0CE6504G12	I.C.3	oxidoreductase
**XAC2052**	A0QH6732B03	VIII.A	conserved hypothetical protein
**XAC2053**	A0AC6827A11	I.D.2	transcription-related protein
**XAC2055**	A0UV9711G01	I.D.1	two-component system, regulatory protein
**XAC2057**	A0UV6709H11	VIII.C	Xanthomonas conserved hypothetical protein
**XAC2062**	A0UV9709F08	I.C.3	cytochrome C552
**XAC2064**	A0AC6822D07	V.A.7	cation efflux system protein
**XAC2065**	A0JE1149F10	V.A.7	transport protein
**XAC2066**	A0QR5702G05	V.A.7	transport protein
**XAC2068**	A0JJ1389E04	I.B.2	6-phosphogluconate dehydratase
**XAC2069**	A0JJ1546A01	I.C.5	6-phosphogluconolactonase
**XAC2070**	A0QR6490H11	I.C.4	glucose kinase
**XAC2071**	A0CE6742C08	I.C.5	glucose-6-phosphate 1-dehydrogenase
**XAC2074**	A0QR5517C12	VIII.A	conserved hypothetical protein
**XAC2076**	A0QH6327E01	I.C.7	succinate dehydrogenase, membrane anchor subunit
**XAC2077**	A0AM1540G12	I.C.7	succinate dehydrogenase, flavoprotein subunit
**XAC2078**	A0JE1334E12	I.C.7	succinate dehydrogenase iron-sulfur protein
**XAC2079**	A0QR5704G12	VIII.A	conserved hypothetical protein
**XAC2080**	A0QR5703A09	VIII.A	conserved hypothetical protein
**XAC2081**	A0UV6458E12	III.D.3	lipoprotein releasing system transmembrane protein
**XAC2082**	A0UV6821G08	V.A.7	ABC transporter ATP-binding protein
**XAC2084**	A0UV6766C01	III.A.5	DNA uptake/competence protein
**XAC2085**	A0UE6759A01	V.A.7	biopolymer transport protein
**XAC2086**	A0UV6733E07	V.A.7	biopolymer transport protein
**XAC2087**	A0QR6729D02	V.A.7	ABC transporter ATP-binding protein
**XAC2089**	A0UV6734F10	IV.C	3-deoxy-manno-octulosonate cytidylyltransferase
**XAC2092**	A0UV6821B04	III.A.4	excinuclease ABC subunit C
**XAC2093**	A0QR5512D11	III.D.2	CDP-diacylglycerol-glycerol-3- phosphate 3-phosphatidyltransferase
**XAC2098**	A0JJ1070E12	II.D.15	ATP-dependent serine activating enzyme
**XAC2103**	A0UV9880B10	III.A.3	DNA recombinase
**XAC2105**	A0CE6831E07	IV.A.1	polysaccharide biosynthetic protein
**XAC2108**	A0QR6737C11	VIII.C	Xanthomonas conserved hypothetical protein
**XAC2109**	A0UV6708H09	VIII.A	conserved hypothetical protein
**XAC2112**	A0UV6843B12	III.B.5	pseudouridylate synthase
**XAC2113**	A0CE6344A06	VIII.A	conserved hypothetical protein
**XAC2114**	A0CE6505F06	VIII.A	conserved hypothetical protein
**XAC2115**	A0UV1650H03	VIII.A	conserved hypothetical protein
**XAC2117**	A0UV9709D02	VIII.A	conserved hypothetical protein
**XAC2121**	A0AR1557H09	VII.C	O-methyltransferase
**XAC2122**	A0JJ1537D08	I.C.3	dehydrogenase
**XAC2124**	A0QR6730H12	VIII.A	conserved hypothetical protein
**XAC2125**	A0UV6736B03	IV.C	glycosyl transferase related protein
**XAC2126**	A0EC6321E11	VIII.A	conserved hypothetical protein
**XAC2126**	genomic_DNA	VIII.A	conserved hypothetical protein
**XAC2127**	A0UV6709H05	VIII.B	hypothetical protein
**XAC2128**	A0UV6818H08	I.A.2	2-keto-gluconate dehydrogenase
**XAC2130**	A0QH6308B05	VIII.A	conserved hypothetical protein
**XAC2131**	A0QR9814C03	VI.C	ISxac3 transposase
**XAC2133**	A0UV6844A02	IX	oxidoreductase
**XAC2134**	A0QR6805E06	VIII.A	conserved hypothetical protein
**XAC2135**	A0UV6733E11	VIII.B	hypothetical protein
**XAC2136**	A0UV6763G07	I.C.3	oxidoreductase

**XAC2140**	A0UV9903G02	III.C.3	D-Ala-D-Ala carboxypeptidase
**XAC2141**	A0QR6730C04	I.D.1	two-component system, regulatory protein
**XAC2143**	A0AC6823C09	VIII.B	hypothetical protein
**XAC2146**	A0CE6828D10	VIII.B	hypothetical protein
**XAC2148**	A0AC6825C12	V.A.7	outer membrane efflux protein
**XAC2149**	A0UV6765G03	I.D.1	nodulation protein
**XAC2150**	genomic_DNA	I.D.1	nodulation protein
**XAC2151**	A0CE6746F11	VII.F	YapH protein
**XAC2152**	A0UV9804D10	VIII.A	conserved hypothetical protein
**XAC2155**	A0CE9724F02	VIII.A	conserved hypothetical protein
**XAC2157**	A0JJ1169A09	II.D.14	uroporphyrin-III C-methyltransferase
**XAC2158**	A0QR6390H12	I.D.3	histidine kinase/response regulator hybrid protein
**XAC2159**	A0UV6349C05	II.D.12	siroheme synthase
**XAC2166**	A0QR6002D09	I.D.2	transcriptional regulator
**XAC2167**	A0AM1113H12	I.D.1	two-component system, sensor protein
**XAC2170**	A0AC0113C04	VIII.C	Xanthomonas conserved hypothetical protein
**XAC2172**	A0JJ0101D04	I.C.1	NADH dehydrogenase
**XAC2174**	A0QR5317B09	VI.C	ISxac3 transposase
**XAC2175**	A0UV6456C10	VI.C	ISxac3 transposase
**XAC2179**	A0CE6743A12	III.A.4	RadC family protein
**XAC2181**	A0CE5309H01	VIII.A	conserved hypothetical protein
**XAC2182**	A0QR6755D05	VIII.A	conserved hypothetical protein
**XAC2184**	A0JE5637F07	VIII.B	hypothetical protein
**XAC2185**	A0QR6202D01	V.A.7	ferrichrome-iron receptor
**XAC2187**	A0UV6843A10	VIII.A	conserved hypothetical protein
**XAC2189**	A0QR6749B12	VIII.B	hypothetical protein
**XAC2190**	A0QH6308F06	VIII.A	conserved hypothetical protein
**XAC2191**	A0QR6389D01	I.D.1	two-component system, regulatory protein
**XAC2193**	A0RN1627F02	V.A.7	TonB-dependent receptor
**XAC2196**	A0QH6836E03	VIII.A	conserved hypothetical protein
**XAC2197**	A0UV6765E04	VII.C	hemolysin- type calcium binding protein
**XAC2198**	A0UV1609A01	VII.C	hemolysin- type calcium binding protein
**XAC2199**	A0UV6710D06	VIII.B	hypothetical protein
**XAC2200**	A0RN1627C07	VIII.B	hypothetical protein
**XAC2201**	A0UE6378F07	VII.C	hemolysin secretion protein D
**XAC2202**	A0QR5902B02	VII.C	hemolysin secretion protein B
**XAC2203**	A0UV6764B05	VIII.B	hypothetical protein
**XAC2205**	A0QR6392E07	V.B	chromosome partitioning related protein
**XAC2206**	A0UV9900C08	VIII.A	conserved hypothetical protein
**XAC2209**	A0UV9820B04	VIII.A	conserved hypothetical protein
**XAC2210**	A0JJ1110B05	VIII.A	conserved hypothetical protein
**XAC2212**	A0CE1622F02	III.A.1	DNA topoisomerase III
**XAC2214**	A0UV6736A10	VI.A	phage-related protein
**XAC2215**	A0AC6430H05	VI.A	phage-related protein
**XAC2217**	A0QR5704F02	VIII.A	conserved hypothetical protein
**XAC2218**	A0QR5704H06	VIII.A	conserved hypothetical protein
**XAC2220**	A0QR5704E08	VIII.B	hypothetical protein
**XAC2222**	A0CE6746E01	VI.A	phage-related integrase
**XAC2223**	A0UT9742F03	VIII.A	conserved hypothetical protein
**XAC2224**	A0UE6379C03	VI.C	ISxac3 transposase
**XAC2227**	A0JJ1035G03	VIII.A	conserved hypothetical protein
**XAC2227**	A0QR6391E10	VIII.A	conserved hypothetical protein
**XAC2228**	A0AC6823F04	I.D.2	transcriptional regulator tetR family
**XAC2229**	A0QR5205C02	I.C.3	NAD(P)H dehydrogenase

**XAC2230**	A0UV9819F04	VII.C	glutathione S-transferase
**XAC2231**	A0QR6728H12	IX	B-cell mitogen related protein
**XAC2231**	genomic_DNA	IX	B-cell mitogen related protein
**XAC2232**	A0UE9829F01	I.D.2	repressor binding protein
**XAC2233**	A0QR9757H03	I.C.3	short chain dehydrogenase
**XAC2234**	A0JJ1101B06	V.A.7	MFS transporter
**XAC2237**	A0UV6845D10	VIII.A	conserved hypothetical protein
**XAC2238**	A0AC0113F11	VI.B	plasmid-related protein
**XAC2239**	A0UV9880E09	VIII.A	conserved hypothetical protein
**XAC2242**	A0CE5309G05	VI.B	plasmid-related protein
**XAC2244**	A0AC6827A10	VIII.B	hypothetical protein
**XAC2245**	A0QR5407C08	VIII.B	hypothetical protein
**XAC2246**	A0QR6803F03	VIII.B	hypothetical protein
**XAC2249**	A0QR6768C03	VIII.B	hypothetical protein
**XAC2251**	A0QH6309B11	VIII.B	hypothetical protein
**XAC2252**	A0UT6817D05	VIII.B	hypothetical protein
**XAC2254**	A0AM1402D10	VIII.B	hypothetical protein
**XAC2256**	A0UV6761G01	VIII.A	conserved hypothetical protein
**XAC2259**	A0JJ1003G10	VIII.A	conserved hypothetical protein
**XAC2261**	A0AM1111F12	VI.B	plasmid-related protein
**XAC2262**	A0UV6709F10	VIII.B	hypothetical protein
**XAC2263**	A0UV6707B04	VIII.B	hypothetical protein
**XAC2264**	A0UV6205C06	VIII.B	hypothetical protein
**XAC2267**	A0QR9759C08	VIII.B	hypothetical protein
**XAC2268**	A0CE6740H03	VIII.B	hypothetical protein
**XAC2269**	A0EC6316F07	VIII.B	hypothetical protein
**XAC2270**	A0AC9763E08	VIII.B	hypothetical protein
**XAC2272**	A0JJ1069G04	VIII.B	hypothetical protein
**XAC2275**	A0CE0105A12	VIII.A	conserved hypothetical protein
**XAC2277**	A0UT9876C09	I.A.2	3-hydroxyisobutirate dehydrogenase
**XAC2278**	A0AC6824F02	VIII.A	conserved hypothetical protein
**XAC2279**	A0QR6752G03	VIII.A	conserved hypothetical protein
**XAC2281**	A0CE6345C01	III.A.4	RadC family protein
**XAC2284**	A0AC6827H05	VIII.C	Xanthomonas conserved hypothetical protein
**XAC2285**	A0UV6843F08	VI.A	phage-related protein
**XAC2287**	A0UV6712B02	II.B.1	glutamine amidotransferase
**XAC2290**	A0UV6762A10	VIII.A	conserved hypothetical protein
**XAC2291**	A0QR9812C06	V.A.7	transport protein
**XAC2294**	A0QR5205A01	IV.C	lipopolysaccharide core biosynthesis protein
**XAC2297**	A0QR6374G07	III.A.3	integration host factor, beta subunit
**XAC2299**	A0UV6846E06	II.B.2	cytidylate kinase
**XAC2300**	A0CE6743A09	III.B.2	50S ribosomal protein L36
**XAC2301**	A0JJ0705F07	VIII.A	conserved hypothetical protein
**XAC2302**	A0QR9775D08	VIII.A	conserved hypothetical protein
**XAC2305**	A0QR5517E11	VI.B	pheromone shutdown protein
**XAC2309**	A0QR6377C08	V.A.3	ABC transporter sugar permease
**XAC2311**	A0UE9890D05	VIII.A	conserved hypothetical protein
**XAC2312**	A0UV6456F01	VIII.A	conserved hypothetical protein
**XAC2313**	A0QR6728F03	I.D.2	transcriptional regulator lacI family
**XAC2315**	A0QR9874F02	VIII.A	conserved hypothetical protein
**XAC2316**	A0UT6817C02	VIII.A	conserved hypothetical protein
**XAC2318**	A0CE6746F10	III.B.5	pseudouridylate synthase
**XAC2319**	A0QR9756F03	VIII.A	conserved hypothetical protein
**XAC2320**	A0QH6326A11	III.C.1	glutamine cyclotransferase

**XAC2321**	A0UV6763D12	IX	hydrolase
**XAC2322**	A0UV6762G02	II.A.2	succinyldiaminopimelate transaminase
**XAC2324**	A0AM1540C10	V.A.6	ABC transporter heme permease
**XAC2326**	A0CE6345D07	VIII.A	conserved hypothetical protein
**XAC2327**	A0UV6711G08	I.C.3	C-type cytochrome biogenesis protein
**XAC2328**	A0JJ1328B09	I.C.3	C-type cytochrome biogenesis membrane protein
**XAC2331**	A0UV6739G08	I.C.3	C-type cytochrome biogenesis protein
**XAC2332**	A0JJ1065G04	II.A.2	homoserine O-acetyltransferase
**XAC2336**	A0UT9850A04	I.C.1	cytochrome D ubiquinol oxidase subunit I
**XAC2337**	A0UT6817E01	I.C.1	cytochrome D ubiquinol oxidase subunit II
**XAC2341**	A0UT1521D03	VII.C	glutaryl-7-ACA acylase precursor
**XAC2342**	A0JJ1536A12	II.A.1	gamma-glutamyl phosphate reductase
**XAC2344**	A0QR6367C11	VIII.A	conserved hypothetical protein
**XAC2349**	A0UV6458G12	II.A.1	acetylornithine deacetylase
**XAC2351**	A0CE5420D02	II.A.1	argininosuccinate synthase
**XAC2352**	A0CE6830B04	II.A.1	ornithine carbamoyltransferase
**XAC2356**	A0JE1380B10	VII.C	drug:proton antiporter
**XAC2358**	A0UE6465F02	IX	DnaK supressor
**XAC2361**	A0JE1208H07	III.C.3	peptidase
**XAC2362**	A0CE6829G05	VIII.A	conserved hypothetical protein
**XAC2363**	A0UV9901G01	VIII.B	hypothetical protein
**XAC2365**	A0CE6740B06	I.A.2	ethanolamine ammonia-lyase large subunit
**XAC2366**	A0UV9901F11	I.A.2	ethanolamine ammonia-lyase light chain
**XAC2367**	A0UT9701C09	VIII.C	Xanthomonas conserved hypothetical protein
**XAC2369**	A0QH6306H03	VII.G	general stress protein
**XAC2370**	A0UE6718H10	VIII.A	conserved hypothetical protein
**XAC2371**	A0UV6735A05	VI.C	IS1479 transposase
**XAC2372**	A0UE6718H01	VI.C	IS1479 transposase
**XAC2373**	A0QR6727H10	VII.D	pectate lyase (degenerated)
**XAC2374**	A0QR5904E12	VII.D	polygalacturonase
**XAC2375**	A0CE5309E12	VIII.A	conserved hypothetical protein
**XAC2380**	A0CE0105C02	III.C.1	elongation factor P
**XAC2381**	A0UV9870E02	VIII.A	conserved hypothetical protein
**XAC2383**	A0JJ1065F04	IX	phosphate-binding protein
**XAC2385**	A0QR6453G04	I.D.2	extragenic supressor protein SuhB
**XAC2386**	A0EC6324C01	VII.C	superoxidase dismutase
**XAC2388**	A0UV9750E01	VIII.A	conserved hypothetical protein
**XAC2390**	A0UV6708H10	III.B.5	ATP-dependent RNA helicase
**XAC2391**	A0QR6002C07	II.B.4	adenine phosphoribosyltransferase
**XAC2392**	A0CE6743A03	VIII.A	conserved hypothetical protein
**XAC2393**	A0QR6443G08	III.D.2	carboxylesterase
**XAC2394**	A0QR6613A01	VII.C	glutathione S-transferase
**XAC2401**	A0AM1365A08	II.E	acetoacetyl-CoA reductase
**XAC2405**	A0AM1084A08	III.A.4	DNA mismatch repair protein MutL
**XAC2406**	A0CE9842C02	IV.B	N-acetylmuramoyl-L-alanine amidase
**XAC2407**	A0QR6730D02	VIII.A	conserved hypothetical protein
**XAC2408**	A0QR6728E02	VIII.A	conserved hypothetical protein
**XAC2409**	A0UV6735H09	V.A.7	iron-sulfur cluster-binding protein
**XAC2410**	A0QR6414A07	III.A.4	exodeoxyribonuclease VII large subunit
**XAC2412**	A0AC0113D09	VIII.A	conserved hypothetical protein
**XAC2413**	A0QR6614A05	III.B.4	ribonuclease D
**XAC2415**	A0UV5503F01	VIII.A	conserved hypothetical protein
**XAC2421**	A0AC6825C07	VIII.B	hypothetical protein
**XAC2422**	A0QR6389E11	VI.B	plasmid-related protein

**XAC2423**	A0JJ1571E03	VI.C	IS1478 transposase
**XAC2424**	A0CE9725H09	VI.C	ISxcd1 transposase
**XAC2429**	A0QR6804A06	VIII.A	conserved hypothetical protein
**XAC2431**	A0UV6710B03	VI.C	transposase
**XAC2432**	A0AC6827D07	VI.C	transposase
**XAC2433**	A0QR6729D08	VI.B	resolvase
**XAC2434**	A0UE9829C06	VIII.A	conserved hypothetical protein
**XAC2435**	A0UV6765G02	VI.B	plasmid-related protein
**XAC2436**	A0AM1084H03	III.A.5	XamI DNA methyltransferase
**XAC2437**	A0EC6475E05	III.A.5	type II restriction enzyme XamI
**XAC2438**	A0QH6836C10	VIII.B	hypothetical protein
**XAC2439**	A0UV6209E03	VIII.A	conserved hypothetical protein
**XAC2440**	A0UV6712C01	VI.B	plasmid mobilization protein
**XAC2442**	A0CE9842A03	VIII.B	hypothetical protein
**XAC2443**	A0JE5637H06	VIII.A	conserved hypothetical protein
**XAC2444**	A0UV6712A10	VIII.A	conserved hypothetical protein
**XAC2445**	A0UV6436A10	VIII.C	Xanthomonas conserved hypothetical protein
**XAC2450**	A0JJ0901E01	III.A.1	ATP-dependent DNA helicase
**XAC2452**	A0QR6002E01	VIII.C	Xanthomonas conserved hypothetical protein
**XAC2453**	A0UV6712F08	I.D.2	stringent starvation protein B
**XAC2454**	A0CE6746B03	I.D.2	stringent starvation protein A
**XAC2455**	A0QR6730G02	I.C.1	ubiquinol cytochrome C oxidoreductase, cytochrome C1 subunit
**XAC2456**	A0JJ0102E05	I.C.1	ubiquinol cytochrome C oxidoreductase, cytochrome B subunit
**XAC2462**	A0QR5408H09	IX	ATP-binding protein
**XAC2464**	A0AC6336B04	VIII.A	conserved hypothetical protein
**XAC2466**	A0QH6732E06	V.A.4	polar amino acid transporter
**XAC2468**	A0JJ1203B12	V.A.4	magnesium and cobalt transport protein
**XAC2469**	A0QH6732C06	I.B.10	succinate-semialdehyde dehydrogenase
**XAC2470**	A0UV5307F09	V.A.1	putrescine transport protein; permease
**XAC2471**	A0UV6734G03	V.A.1	polyamine transport protein
**XAC2473**	A0UV9819H06	VII.C	outer membrane protein OprN precursor
**XAC2475**	A0UE6399B02	VII.C	transport protein
**XAC2480**	A0UT6815H11	II.A.1	glutamine synthase
**XAC2481**	A0JJ1205A02	I.C.3	oxidoreductase
**XAC2482**	A0UV6761D10	I.D.2	transcriptional regulator
**XAC2485**	A0UV6820E12	VIII.C	Xanthomonas conserved hypothetical protein
**XAC2486**	A0UV6210H06	I.C.2	formate dehydrogenase a chain
**XAC2487**	A0QR6755B11	I.C.2	formate dehydrogenase b chain
**XAC2488**	A0UV6364F08	V.A.7	integral membrane transporter
**XAC2489**	A0JE5637G08	II.A.2	omega-amino acid-pyruvate aminotransferase
**XAC2490**	A0QR5902C08	VIII.C	Xanthomonas conserved hypothetical protein
**XAC2491**	A0QH9738D01	VIII.A	conserved hypothetical protein
**XAC2493**	genomic_DNA	I.D.1	two-component system, regulatory protein
**XAC2494**	A0UV6762H07	VII.C	drug resistance translocase
**XAC2495**	A0QH6383G11	VIII.B	hypothetical protein
**XAC2496**	A0QR1369H04	VIII.A	conserved hypothetical protein
**XAC2497**	A0QH6836G10	I.D.2	transcriptional regulator tetR/acrR family
**XAC2498**	A0QR5902H09	VII.C	multidrug resistance protein
**XAC2499**	A0EC6426D07	VII.C	multidrug efflux transporter
**XAC2500**	A0UT9704H04	I.D.2	transcriptional regulator lacI family
**XAC2502**	A0UV6762F01	I.C.4	1-phosphofructokinase (fructose 1-phosphate kinase)
**XAC2504**	genomic_DNA	VII.H	regulator of pathogenicity factors
**XAC2504**	A0UV1502G04	VII.H	regulator of pathogenicity factors
**XAC2505**	A0JJ1441A12	VIII.B	hypothetical protein

**XAC2506**	A0QR6204E12	VIII.B	hypothetical protein
**XAC2508**	A0QR6803A04	VI.C	transposase
**XAC2512**	A0QH6836G12	V.A.6	preprotein translocase YajC subunit
**XAC2513**	A0UV6709C04	VII.H	queuine tRNA-ribosyltransferase
**XAC2514**	A0QR5407F08	III.B.4	S-adenosylmethionine:tRNA ribosyltransferase-isomerase
**XAC2515**	A0UE9858E07	I.D.2	transcriptional regulator asnC/lrp family
**XAC2516**	A0JE1208D07	I.A.2	L-lysine 6-aminotransferase
**XAC2520**	A0CE6503F05	V.A.7	TonB-dependent receptor
**XAC2522**	A0QR6702G12	VII.D	cellulase
**XAC2523**	A0UV6206F11	II.D.10	gamma-glutamyltranspeptidase
**XAC2525**	A0EC6322C08	VIII.A	conserved hypothetical protein
**XAC2528**	A0QR6304D02	III.C.2	heat shock protein G
**XAC2529**	A0UE6718C07	IX	RhsD protein
**XAC2530**	A0UV6712D08	VIII.A	conserved hypothetical protein
**XAC2531**	A0QH6732C04	V.A.7	TonB-dependent receptor
**XAC2535**	A0AC6825E06	V.A.7	TonB-dependent receptor
**XAC2536**	A0UE6758C07	VIII.C	Xanthomonas conserved hypothetical protein
**XAC2537**	A0AC6822E07	III.C.3	peptidase
**XAC2539**	A0UV6762E09	VIII.C	Xanthomonas conserved hypothetical protein
**XAC2541**	A0UV6512F12	III.C.3	peptidase
**XAC2547**	A0JE1206C10	II.A.2	dihydrodipicolinate synthetase
**XAC2551**	A0UV6763F01	I.D.2	transcriptional regulator
**XAC2552**	A0UV6733F01	V.B	cell division protein
**XAC2553**	A0CE6740B12	III.A.4	A/G-specific adenine glycosylase
**XAC2554**	A0QR5104D09	VIII.A	conserved hypothetical protein
**XAC2555**	A0AC0115A08	I.D.3	sensor histidine kinase
**XAC2555**	genomic_DNA	I.D.3	sensor histidine kinase
**XAC2555**	genomic_DNA	I.D.3	sensor histidine kinase
**XAC2556**	A0QR6839B09	VIII.C	Xanthomonas conserved hypothetical protein
**XAC2557**	A0UV9871E12	VIII.A	conserved hypothetical protein
**XAC2558**	A0QH6810D11	III.A.4	excinuclease ABC subunit C homolog
**XAC2559**	A0QR9814G07	VIII.C	Xanthomonas conserved hypothetical protein
**XAC2562**	A0UE6718D02	VIII.A	conserved hypothetical protein
**XAC2563**	A0QR5902F05	I.A.3	acyl-CoA dehydrogenase
**XAC2565**	A0QR6803C07	I.B.10	deoxyxylulose-5-phosphate synthase
**XAC2566**	A0QH6732G01	IV.C	glycosyltransferase
**XAC2567**	A0QR5904C05	VIII.C	Xanthomonas conserved hypothetical protein
**XAC2568**	A0QR9756B10	VIII.C	Xanthomonas conserved hypothetical protein
**XAC2570**	A0CE6341H04	IX	GumP protein
**XAC2572**	A0UE6759E02	IX	GumN protein
**XAC2574**	A0QR5701A12	VII.E	GumM protein
**XAC2575**	A0QR6404E10	VII.E	GumL protein
**XAC2576**	A0QR6730B08	VII.E	GumK protein
**XAC2577**	A0UV6821A07	VII.E	GumJ protein
**XAC2578**	A0QH6381H05	VII.E	GumI protein
**XAC2579**	A0UV9746E08	VII.E	GumH protein
**XAC2580**	A0CE6831A10	VII.E	GumG protein
**XAC2581**	A0QH6411H07	VII.E	GumF protein
**XAC2582**	A0UV9881F05	VII.E	GumE protein
**XAC2583**	A0AC6823C03	VII.E	GumD protein
**XAC2585**	A0UV6712G11	VII.E	GumB protein
**XAC2589**	A0AM1112D12	III.B.4	phenylalanyl-tRNA synthetase betachain
**XAC2594**	A0QR6840D03	III.B.4	threonyl-tRNA synthetase
**XAC2596**	A0UV6487F02	III.D.1	cyclomaltodextrin glucanotransferase (CGTase)

**XAC2598**	A0EC6323B03	VIII.C	Xanthomonas conserved hypothetical protein
**XAC2599**	A0JJ1343E05	I.A.1	alpha-glucosidase
**XAC2600**	A0UV1356E10	V.A.7	TonB-dependent receptor
**XAC2604**	A0QR5903A07	VI.C	ISxac4 transposase
**XAC2605**	A0QH6713B11	VIII.A	conserved hypothetical protein
**XAC2605**	genomic_DNA	VIII.A	conserved hypothetical protein
**XAC2606**	A0CE6480B03	VIII.C	Xanthomonas conserved hypothetical protein
**XAC2606**	genomic_DNA	VIII.C	Xanthomonas conserved hypothetical protein
**XAC2606**	genomic_DNA	VIII.A	conserved hypothetical protein
**XAC2607**	A0CE6746F08	VII.H	VirB6 protein
**XAC2609**	genomic_DNA	IV.B	carboxypeptidase
**XAC2609**	A0CE6828E07	IV.B	carboxypeptidase
**XAC2610**	genomic_DNA	VIII.B	hypothetical protein
**XAC2610**	A0QR6770A07	VIII.B	hypothetical protein
**XAC2610**	genomic_DNA	VIII.B	hypothetical protein
**XAC2611**	genomic_DNA	VIII.C	Xanthomonas conserved hypothetical protein
**XAC2611**	A0QR5902E05	VIII.C	Xanthomonas conserved hypothetical protein
**XAC2612**	A0QR5903E03	VII.H	VirB6 protein
**XAC2613**	genomic_DNA	VIII.B	hypothetical protein
**XAC2614**	genomic_DNA	VII.H	VirB4 protein
**XAC2614**	genomic_DNA	VII.H	VirB4 protein
**XAC2614**	A0AM1489E07	VII.H	VirB4 protein
**XAC2616**	genomic_DNA	VII.H	VirB2 protein
**XAC2617**	A0UV6709A03	VII.H	VirB1 protein
**XAC2617**	genomic_DNA	VII.H	VirB1 protein
**XAC2618**	genomic_DNA	VII.H	VirB11 protein
**XAC2618**	A0UV6711G09	VII.H	VirB11 protein
**XAC2618**	genomic_DNA	VII.H	VirB11 protein
**XAC2619**	A0QR6001A01	VII.H	VirB10 protein
**XAC2620**	genomic_DNA	VII.H	VirB9 protein
**XAC2620**	A0QH9737H04	VII.H	VirB9 protein
**XAC2620**	genomic_DNA	VII.H	VirB9 protein
**XAC2621**	A0CE6742C01	VII.H	VirB8 protein
**XAC2622**	A0UV9871D06	VIII.C	Xanthomonas conserved hypothetical protein
**XAC2622**	genomic_DNA	VIII.C	Xanthomonas conserved hypothetical protein
**XAC2623**	A0QR6204E07	VII.H	VirD4 protein
**XAC2623**	genomic_DNA	VII.H	VirD4 protein
**XAC2625**	A0UV5503H06	III.A.4	excinuclease ABC subunit B
**XAC2625**	genomic_DNA	III.A.4	excinuclease ABC subunit B
**XAC2626**	A0UV6821E09	IV.D	fimbrial biogenesis protein
**XAC2628**	A0UV6766H06	VI.A	phage-related integrase
**XAC2633**	A0UE9854H08	VI.C	ISxac3 transposase
**XAC2634**	A0AR1011B09	VI.C	ISxac3 transposase
**XAC2636**	A0JJ1569A11	VIII.B	hypothetical protein
**XAC2637**	A0QR6303D04	VIII.B	hypothetical protein
**XAC2638**	A0AC6406G08	VIII.B	hypothetical protein
**XAC2639**	A0QR6418G06	III.A.5	site-specific DNA-methyltransferase
**XAC2640**	A0AC6407E11	VIII.C	Xanthomonas conserved hypothetical protein
**XAC2641**	A0UV6709G10	VI.A	phage-related capsid packaging protein
**XAC2642**	A0QH6813A04	VI.A	phage-related terminase
**XAC2646**	A0QR5902H11	VI.A	phage-related capsid completion protein
**XAC2647**	A0QH6836C04	VI.A	phage-related tail protein
**XAC2648**	A0QR9815H11	VI.A	phage-related protein
**XAC2650**	A0QR6347B11	VI.A	phage-related lytic enzyme

**XAC2652**	A0UE9854D02	VI.A	phage-related tail protein
**XAC2654**	A0RN1498F11	VIII.A	conserved hypothetical protein
**XAC2655**	A0QR6704E07	VI.A	phage-related baseplate assembly protein
**XAC2657**	A0UV6485G11	VIII.A	conserved hypothetical protein
**XAC2657**	genomic_DNA	VIII.A	conserved hypothetical protein
**XAC2658**	A0QR6804B10	VIII.B	hypothetical protein
**XAC2660**	A0UT9875A08	VI.A	phage-related baseplate assembly protein
**XAC2661**	A0CE6344D09	VI.C	ISxac3 transposase
**XAC2663**	A0CE6830E06	VI.C	transposase
**XAC2664**	A0AM1405F09	IV.A.2	PilE protein
**XAC2666**	A0QR6737A09	IV.D	PilX protein
**XAC2668**	A0UV5307C07	IV.D	pre-pilin leader sequence
**XAC2669**	A0UV6818C11	IV.A.2	pre-pilin like leader sequence
**XAC2670**	A0QR6727F06	IX	alginate biosynthesis protein
**XAC2671**	A0EC6473D05	VIII.A	conserved hypothetical protein
**XAC2672**	A0QR6613G03	IV.A.2	Oar protein
**XAC2674**	A0AC6824E01	VIII.A	conserved hypothetical protein
**XAC2680**	A0UV6707B10	VIII.A	conserved hypothetical protein
**XAC2681**	A0QR6770A01	II.B.2	nicotinate-nucleotide pyrophosphorylase
**XAC2683**	A0QR6772H12	III.B.6	polynucleotide phosphorylase
**XAC2684**	A0UV6514H12	III.B.2	30S ribosomal protein S15
**XAC2685**	A0UT6817H04	III.B.4	tRNA pseudouridine synthase B
**XAC2687**	A0JJ1197C07	III.C.1	protein chain initiation factor IF-2
**XAC2691**	A0UV6486F10	I.C.1	NADH-ubiquinone oxidoreductase, NQO14 subunit
**XAC2693**	A0QR6725B08	I.C.1	NADH-ubiquinone oxidoreductase, NQO12 subunit
**XAC2694**	A0QR6418E03	I.C.1	NADH-ubiquinone oxidoreductase, NQO11 subunit
**XAC2695**	A0QR5704C06	I.C.1	NADH-ubiquinone oxidoreductase, NQO10 subunit
**XAC2697**	A0AC9896G11	I.C.1	NADH-ubiquinone oxidoreductase, NQO8 subunit
**XAC2698**	A0CE6742F03	I.C.1	NADH-ubiquinone oxidoreductase, NQO3 subunit
**XAC2699**	A0UV9869E01	I.C.1	NADH-ubiquinone oxidoreductase, NQO1 subunit
**XAC2700**	A0RN1551E05	I.C.1	NADH-ubiquinone oxidoreductase, NQO2 subunit
**XAC2701**	A0JJ1591E02	I.C.1	NADH-ubiquinone oxidoreductase, NQO4 subunit
**XAC2708**	A0UV6733C02	I.C.3	dehydrogenase
**XAC2709**	A0AC6827F10	IV.A.2	cyanoglobin
**XAC2710**	A0UV5307G04	VIII.C	Xanthomonas conserved hypothetical protein
**XAC2712**	A0UE9890C09	VIII.C	Xanthomonas conserved hypothetical protein
**XAC2713**	A0UV6736B08	I.C.3	oxidoreductase
**XAC2715**	A0QH6731H12	II.E	acetyl-coenzyme A carboxylase carboxyl transferase
**XAC2716**	A0QR9756A07	II.A.4	tryptophan synthase alpha chain
**XAC2718**	A0JJ0102H10	I.D.2	transcriptional regulator
**XAC2720**	A0AM1488G08	III.B.4	tRNA pseudouridine synthase A
**XAC2722**	A0JJ1110G03	IV.D	FimV protein
**XAC2723**	A0UE6718E03	II.A.2	aspartate semialdehyde dehydrogenase
**XAC2724**	A0QR6752G06	II.A.3	2-hydroxyacid dehydrogenase
**XAC2725**	A0UV6818H06	II.A.4	chorismate synthase
**XAC2727**	A0QH6810A11	VIII.A	conserved hypothetical protein
**XAC2728**	A0UV6845E12	II.E	phosphatidylserine decarboxylase
**XAC2729**	A0UT9850H09	IV.B	membrane-bound lytic murein transglycosylase D precursor
**XAC2731**	A0UV6762E02	VIII.A	conserved hypothetical protein
**XAC2731**	genomic_DNA	VIII.A	conserved hypothetical protein
**XAC2732**	A0UV9881F06	VIII.C	Xanthomonas conserved hypothetical protein
**XAC2733**	A0UE6757A02	IV.A.1	integral membrane protein
**XAC2734**	A0EC6474F01	III.B.5	transcription elongation factor and transcript cleavage
**XAC2735**	A0CE9841F02	VIII.A	conserved hypothetical protein

**XAC2736**	A0QR5702A03	I.A.2	carboxymethylenebutenolidase
**XAC2737**	A0QR6730H02	VIII.A	conserved hypothetical protein
**XAC2738**	A0UT6815D08	VIII.C	Xanthomonas conserved hypothetical protein
**XAC2739**	A0JJ0101G03	VIII.C	Xanthomonas conserved hypothetical protein
**XAC2741**	A0QR6375E06	VIII.A	conserved hypothetical protein
**XAC2742**	A0AC6337B06	V.A.7	TonB-dependent receptor
**XAC2743**	A0UV6766G06	IV.A.2	Oar protein
**XAC2744**	A0QH9738G07	II.D.17	phytoene dehydrogenase
**XAC2746**	A0QR5702E03	III.C.3	metallopeptidase
**XAC2748**	A0UV9882F03	IV.A.1	integral membrane protein
**XAC2750**	A0UV6351G03	I.C.3	reductase
**XAC2752**	A0UV6708F12	V.A.7	transport protein
**XAC2753**	A0UV9872C09	III.D.3	lipoprotein
**XAC2754**	A0CE6743G02	III.C.1	peptidyl-prolyl cis-trans isomerase
**XAC2755**	A0QR6770B02	VIII.A	conserved hypothetical protein
**XAC2756**	A0QR6705E04	II.E	acyl-CoA thioester hydrolase
**XAC2758**	A0QR6729E12	V.A.1	glutamate symport protein
**XAC2759**	A0JJ1599F12	I.B.9	alkaline phosphatase
**XAC2762**	A0UV6351B08	II.D.11	geranyltranstransferase (farnesyl-diphosphate synthase)
**XAC2767**	A0UV6739C08	III.C.3	TldD protein
**XAC2768**	A0QR5529E06	VIII.A	conserved hypothetical protein
**XAC2772**	A0UV6708A07	IV.A.2	outer membrane protein
**XAC2772**	A0QH6731G11	IV.A.2	outer membrane protein
**XAC2774**	A0UV6761A09	V.A.7	TonB like protein
**XAC2775**	A0AC6825C03	VIII.A	conserved hypothetical protein
**XAC2778**	A0CE6480G11	II.B.4	nicotinate-nucleotide adenylyltransferase
**XAC2779**	A0QR6737C04	III.A.1	DNA polymerase III, delta subunit
**XAC2781**	A0JJ1015G02	III.B.4	leucyl-tRNA synthetase
**XAC2783**	A0QR5206H01	II.D.10	thioredoxin
**XAC2784**	A0QH9730G11	VIII.A	conserved hypothetical protein
**XAC2785**	A0QR5520C04	VIII.B	hypothetical protein
**XAC2786**	A0QR5902G02	VIII.C	Xanthomonas conserved hypothetical protein
**XAC2787**	A0AC0113C03	VIII.B	hypothetical protein
**XAC2788**	A0QR6704E05	VIII.A	conserved hypothetical protein
**XAC2789**	A0QR5702A11	III.C.1	peptidyl-prolyl cis-trans isomerase
**XAC2792**	A0QR5702G11	VIII.A	conserved hypothetical protein
**XAC2793**	A0JJ0104E12	VIII.C	Xanthomonas conserved hypothetical protein
**XAC2795**	A0EC6476E08	VIII.A	conserved hypothetical protein
**XAC2796**	A0UE9886G07	VIII.A	conserved hypothetical protein
**XAC2798**	A0UV6762B01	VIII.A	conserved hypothetical protein
**XAC2801**	genomic_DNA	VIII.A	conserved hypothetical protein
**XAC2802**	A0JJ0101F06	IV.A.2	outer membrane channel protein
**XAC2804**	A0QR5320G09	I.D.1	two-component system, sensor protein
**XAC2805**	A0UV6733C12	V.A.4	cation:proton antiporter
**XAC2806**	A0UV6733A09	VII.C	beta-lactamase
**XAC2807**	A0UE9858E12	III.A.5	S-adenosylmethionine:2-demethylmenaquinone methyltransferase
**XAC2808**	A0QR6106H04	VIII.A	conserved hypothetical protein
**XAC2813**	A0AC6440G05	III.B.5	ATP-dependent RNA helicase
**XAC2814**	A0QH6835C10	I.D.4	RNA polymerase ECF-type sigma factor
**XAC2815**	A0QR5701F02	VIII.C	Xanthomonas conserved hypothetical protein
**XAC2816**	A0QR6770C02	VIII.A	conserved hypothetical protein
**XAC2818**	A0QR9756F07	IV.A.1	inner membrane protein
**XAC2820**	A0AC1384C06	VIII.C	Xanthomonas conserved hypothetical protein
**XAC2822**	A0AC6440D03	III.A.4	DNA methylation and regulatory protein

**XAC2823**	A0QH6731H04	III.A.4	6-O-methylguanine-DNA methyltransferase
**XAC2824**	A0QR5903G08	II.B.4	phosphodiesterase-nucleotide pyrophosphatase
**XAC2826**	A0QR5902E10	I.C.2	alcohol dehydrogenase
**XAC2828**	A0JE1129A05	VIII.A	conserved hypothetical protein
**XAC2831**	A0AC0113C05	III.C.3	extracellular serine protease
**XAC2833**	A0UV6458D01	III.C.3	extracellular serine protease
**XAC2835**	A0UV6712B07	I.C.3	oxidoreductase
**XAC2836**	A0UT9848A06	V.A.7	MFS transporter
**XAC2839**	A0CE6345D04	I.D.2	transcriptional regulator
**XAC2840**	A0AC6824D11	VIII.A	conserved hypothetical protein
**XAC2843**	A0JJ1197H03	VII.C	multidrug efflux transporter
**XAC2846**	A0UV6711F10	I.D.2	transcriptional regulator fur family
**XAC2851**	A0UV9905A07	VIII.C	Xanthomonas conserved hypothetical protein
**XAC2853**	A0RN1227A01	III.C.3	cysteine protease
**XAC2855**	A0UV6735B06	I.D.1	two-component system, regulatory protein
**XAC2857**	A0CE6831B10	VIII.A	conserved hypothetical protein
**XAC2858**	A0JE1637F04	III.A.4	transcription-repair coupling factor
**XAC2859**	A0QR6749F10	VIII.B	hypothetical protein
**XAC2860**	A0UV6821G02	VIII.B	hypothetical protein
**XAC2861**	A0AC6827E01	VIII.B	hypothetical protein
**XAC2862**	A0QR6728A12	VIII.B	hypothetical protein
**XAC2863**	A0QR9732F12	VIII.B	hypothetical protein
**XAC2864**	A0QH6713F05	VIII.C	Xanthomonas conserved hypothetical protein
**XAC2865**	A0AC6827F09	V.C	chemotaxis histidine protein kinase
**XAC2868**	A0QR6002G07	IX	response regulator
**XAC2871**	A0UV6208G11	III.D.2	cardiolipin synthase
**XAC2873**	A0QH1645H07	VIII.C	Xanthomonas conserved hypothetical protein
**XAC2875**	A0UV6821F08	III.A.4	endonuclease V
**XAC2876**	A0CE5319H04	VIII.B	hypothetical protein
**XAC2877**	A0UT6817A04	III.A.2	pirin-related protein
**XAC2878**	A0QH6813H02	VIII.A	conserved hypothetical protein
**XAC2879**	A0UV6710D10	VIII.A	conserved hypothetical protein
**XAC2880**	A0QR6303E06	III.A.2	pirin
**XAC2881**	A0UV6709F07	VII.G	carbon starvation protein A
**XAC2883**	A0UE9829H01	IX	hydrolase
**XAC2885**	A0UV6435G05	III.D.2	phospholipase A1
**XAC2886**	A0QR6755C06	VIII.A	conserved hypothetical protein
**XAC2888**	A0UE6758B09	VIII.A	conserved hypothetical protein
**XAC2889**	A0UV9882E10	VI.C	ISxac2 transposase
**XAC2891**	A0AM1112H04	VIII.A	conserved hypothetical protein
**XAC2893**	A0AC6439C07	I.C.3	oxidoreductase
**XAC2896**	A0QR9816A01	I.C.3	alcohol dehydrogenase
**XAC2900**	A0CE6743D05	III.A.5	type I restriction-modification system DNA methylase
**XAC2901**	A0QR6704A09	VIII.A	conserved hypothetical protein
**XAC2902**	A0UV6209D03	VIII.A	conserved hypothetical protein
**XAC2903**	A0AR1644F08	VIII.A	conserved hypothetical protein
**XAC2904**	A0QR5407A10	VI.A	integrase/recombinase
**XAC2905**	A0CE5420H09	III.A.2	single-stranded DNA binding protein
**XAC2907**	A0UV9709A11	VIII.A	conserved hypothetical protein
**XAC2908**	A0JJ0133H04	IV.B	UDP-N-acetylmuramoylalanine--D-glutamate ligase
**XAC2911**	A0AM1404C04	II.A.2	bifunctional diaminopimelate decarboxylase/asparta
**XAC2912**	A0UT6817F07	VIII.A	conserved hypothetical protein
**XAC2913**	A0UV6761D09	VIII.A	conserved hypothetical protein
**XAC2914**	A0UV5307E08	VIII.A	conserved hypothetical protein

**XAC2915**	A0UV6739A04	V.D	osmotically inducible protein
**XAC2916**	A0JE1075C02	II.B.2	aspartate carbamoyltransferase
**XAC2917**	A0UV6348D11	VIII.A	conserved hypothetical protein
**XAC2918**	A0UV9710H10	I.D.2	transcriptional regulator
**XAC2919**	A0QR9863F07	III.A.4	DNA-3-methyladenine glycosylase I
**XAC2920**	A0QR5407H11	VIII.A	conserved hypothetical protein
**XAC2921**	A0UE6759B01	VIII.A	conserved hypothetical protein
**XAC2923**	A0QH6835F06	IV.D	twitching motility protein
**XAC2924**	A0UV9822G07	IV.D	twitching motility protein
**XAC2926**	A0QH1368D04	II.A.1	pyrroline-5-carboxylate reductase
**XAC2927**	A0UV6843H08	III.A.2	histone-like protein
**XAC2932**	A0UV6488A10	III.C.3	protease
**XAC2933**	A0UV5526E01	VIII.A	conserved hypothetical protein
**XAC2934**	A0UV9747G02	VIII.A	conserved hypothetical protein
**XAC2935**	A0QH6811D03	V.A.7	ABC transporter permease
**XAC2937**	A0AC0113H06	V.A.7	ABC transporter permease
**XAC2938**	A0QH6731G06	II.A.2	cysteine desulfurase
**XAC2939**	A0CE5420H08	IX	acetyltransferase
**XAC2944**	A0UT9846H03	VIII.A	conserved hypothetical protein
**XAC2945**	A0UV1356F01	VIII.A	conserved hypothetical protein
**XAC2946**	A0UE6399A01	VIII.A	conserved hypothetical protein
**XAC2949**	A0CE6742C10	IX	calcium-binding protein
**XAC2950**	A0UV6821A12	VIII.C	Xanthomonas conserved hypothetical protein
**XAC2951**	A0CE6830E04	V.A.5	DNA transport competence protein
**XAC2952**	A0UV9900H03	II.A.2	succinyl-diaminopimelate desuccinylase
**XAC2954**	A0CE6743G07	VIII.A	conserved hypothetical protein
**XAC2956**	A0CE9841G04	III.A.1	replication related protein
**XAC2958**	A0EC6473A09	VIII.A	conserved hypothetical protein
**XAC2959**	A0UV6766H12	II.B.1	phosphoribosylformylglycinamide cyclo-ligase
**XAC2960**	A0QR6725B05	VIII.C	Xanthomonas conserved hypothetical protein
**XAC2961**	A0UV6433C09	II.B.1	5'-phosphoribosylglycinamide transformylase
**XAC2963**	A0QR6805C08	VIII.A	conserved hypothetical protein
**XAC2964**	A0UE9886H11	VIII.A	conserved hypothetical protein
**XAC2965**	A0QR6705D09	IV.C	UDP-N-acetylglucosamine 1-carboxyvinyltransferase
**XAC2966**	A0QH6313H04	VIII.A	conserved hypothetical protein
**XAC2968**	A0QR5317D10	VIII.A	conserved hypothetical protein
**XAC2969**	A0UV6733A08	VIII.A	conserved hypothetical protein
**XAC2970**	A0CE6828E06	VIII.A	conserved hypothetical protein
**XAC2972**	A0UV6739G09	I.D.4	RNA polymerase sigma-54 factor
**XAC2973**	A0AM1365G11	I.D.4	sigma-54 modulation protein
**XAC2974**	A0UV9709G05	I.D.4	nitrogen regulatory IIA protein
**XAC2975**	A0UV6733B04	V.A.3	HPr kinase/phosphatase
**XAC2978**	A0CE6345B10	V.A.3	phosphotransferase system HPr enzyme
**XAC2980**	A0UV1502C04	V.A.4	Mg++ transporter
**XAC2981**	A0AM1424E09	VIII.A	conserved hypothetical protein
**XAC2983**	A0UE6397B12	I.C.1	quinol oxidase, subunit I
**XAC2984**	A0UV6764C03	III.C.3	peptidase
**XAC2985**	A0QR6803H02	V.A.1	amino acid transporter
**XAC2986**	A0AC0115F04	VII.D	pectate lyase II
**XAC2987**	A0UV6349H02	III.C.3	proline imino-peptidase
**XAC2990**	A0QR6724G05	VIII.B	hypothetical protein
**XAC2991**	A0UV5307H09	VIII.B	hypothetical protein
**XAC2992**	A0QR6705F10	III.C.3	endoproteinase Arg-C
**XAC2993**	A0QR6002E06	VIII.C	Xanthomonas conserved hypothetical protein

**XAC2995**	A0UV6708D03	II.D.17	tryptophan halogenase
**XAC2996**	A0UE6759A08	VIII.A	conserved hypothetical protein
**XAC2997**	A0UV6711F06	VIII.A	conserved hypothetical protein
**XAC2998**	A0QR9864E12	V.A.7	TonB-dependent receptor
**XAC3001**	A0EC6323A09	V.A.7	MFS transporter
**XAC3006**	A0UV6763D05	V.A.7	potassium channel related protein
**XAC3009**	A0UV6486C09	II.D.6	pyridoxamine 5'-phosphate oxidase
**XAC3010**	A0QH9730E12	II.A.4	shikimate kinase
**XAC3011**	A0QH1368B09	II.A.4	3-dehydroquinate synthase
**XAC3013**	A0QR5701C06	II.D.12	uroporphyrinogen decarboxylase
**XAC3015**	A0AC6495A07	III.A.3	RebB protein
**XAC3016**	A0QH6308B03	III.A.3	RebA protein
**XAC3017**	A0UV6819B10	III.A.3	RebB protein
**XAC3019**	A0UT6815H01	VIII.B	hypothetical protein
**XAC3020**	A0JJ1070B04	VIII.B	hypothetical protein
**XAC3022**	A0QH6810E09	VIII.B	hypothetical protein
**XAC3023**	A0UV6205D02	VIII.B	hypothetical protein
**XAC3024**	A0QR5206B05	VIII.B	hypothetical protein
**XAC3025**	A0QR6729C09	VIII.B	hypothetical protein
**XAC3027**	A0QR9776F05	V.A.7	MFS transporter
**XAC3029**	A0JE5637E11	I.D.3	histidine kinase/response regulator hybrid protein
**XAC3031**	A0QR6730D08	I.D.3	histidine kinase/response regulator hybrid protein
**XAC3032**	A0AC6825A11	VIII.A	conserved hypothetical protein
**XAC3033**	A0UV6739B05	I.C.3	cytochrome B561
**XAC3034**	A0JJ0102F06	VIII.A	conserved hypothetical protein
**XAC3035**	A0QR9859C02	VIII.A	conserved hypothetical protein
**XAC3036**	A0UV6363B10	I.A.2	L-serine dehydratase
**XAC3037**	A0UV6709E10	IX	hydrolase
**XAC3038**	A0QR5704D11	II.A.2	homoserine dehydrogenase
**XAC3039**	A0UV6736C01	II.A.2	cystathionine gamma-synthase
**XAC3042**	A0QR9753D04	III.C.1	peptide chain release factor 3
**XAC3044**	A0AC0115G04	VIII.A	conserved hypothetical protein
**XAC3045**	A0QR6730C05	VIII.A	conserved hypothetical protein
**XAC3048**	A0QR5702E11	VII.G	heat shock protein
**XAC3049**	A0JE5637A05	VIII.A	conserved hypothetical protein
**XAC3051**	A0QR5407F02	VIII.A	conserved hypothetical protein
**XAC3052**	A0QR5205G06	I.D.2	transcriptional regulator
**XAC3053**	A0UE6718C01	VIII.A	conserved hypothetical protein
**XAC3054**	A0AC0115G05	I.A.3	acyl-CoA dehydrogenase
**XAC3056**	A0UT6211G01	VIII.A	conserved hypothetical protein
**XAC3057**	A0QR6705B06	VII.C	beta-lactamase
**XAC3058**	A0QR6203F09	III.A.2	histone H1
**XAC3060**	A0UV6761B08	I.B.10	glycine cleavage H protein
**XAC3063**	A0AC6408C01	VIII.A	conserved hypothetical protein
**XAC3064**	A0UV9805C07	VIII.A	conserved hypothetical protein
**XAC3065**	A0UT9878E04	VIII.A	conserved hypothetical protein
**XAC3067**	A0QR9864F01	III.A.4	ADP compounds hydrolase
**XAC3069**	A0QH9738F03	VIII.A	conserved hypothetical protein
**XAC3071**	A0UV6739C12	V.A.7	TonB-dependent receptor
**XAC3072**	A0UE9889B03	I.A.1	alpha-L-fucosidase
**XAC3073**	A0CE6477A05	VIII.A	conserved hypothetical protein
**XAC3074**	A0JE1034C03	IV.A.2	beta-hexosaminidase
**XAC3075**	A0CE9723E05	I.A.1	beta-mannosidase
**XAC3080**	A0QR5205C09	I.A.2	ribokinase

**XAC3082**	A0UV6762A05	VIII.A	conserved hypothetical protein
**XAC3083**	A0UT9846C04	VIII.A	conserved hypothetical protein
**XAC3084**	A0UV9822A05	I.A.1	beta-galactosidase
**XAC3085**	A0EC1095B04	VIII.C	Xanthomonas conserved hypothetical protein
**XAC3086**	A0UV6708B09	VIII.C	Xanthomonas conserved hypothetical protein
**XAC3088**	A0UV6707A05	VIII.B	hypothetical protein
**XAC3089**	A0UE5505E07	VIII.A	conserved hypothetical protein
**XAC3092**	A0EC6473G01	III.D.1	asparaginase
**XAC3093**	A0QR6805D02	VIII.B	hypothetical protein
**XAC3094**	A0UV6735E02	VIII.B	hypothetical protein
**XAC3095**	A0QR6603B06	III.B.3	ribosomal protein alanine acetyltransferase
**XAC3097**	A0QH6732E07	VIII.A	conserved hypothetical protein
**XAC3098**	A0JE5637F02	IV.D	PilL protein
**XAC3099**	A0UV6710C12	IV.D	pilus biogenesis protein
**XAC3100**	A0UV6739A02	IV.D	pilus biogenesis protein
**XAC3102**	A0UV6735C05	IV.D	pilus protein
**XAC3103**	A0QR5903G01	II.D.10	glutathione synthetase
**XAC3104**	A0UE6718A11	VII.C	TonB protein
**XAC3105**	A0QR5904B07	I.D.4	dinitrogenase reductase activationg glycohydrolase
**XAC3106**	A0UV6821C09	VIII.A	conserved hypothetical protein
**XAC3107**	A0QR6730G04	III.A.1	ATP-dependent helicase
**XAC3108**	A0UT9846E12	VIII.A	conserved hypothetical protein
**XAC3109**	A0EC1091D09	IV.A.1	penicillin-binding protein 1B
**XAC3110**	A0UV6734D11	IX	glycosyltransferase
**XAC3112**	A0UV5307D11	VIII.A	conserved hypothetical protein
**XAC3113**	A0JJ1110H07	I.D.4	ATP:GTP 3'-pyrophosphotranferase
**XAC3114**	A0QR6410F09	II.D.11	pyrroloquinoline quinone biosynthesis protein G
**XAC3115**	A0QR9717D03	II.D.11	PqqC protein
**XAC3116**	A0QR5111B02	II.D.11	PqqC/D protein
**XAC3117**	A0UV6843C10	II.D.11	PqqE protein
**XAC3118**	A0QR6376D07	VIII.C	Xanthomonas conserved hypothetical protein
**XAC3119**	A0UV9884D08	VIII.A	conserved hypothetical protein
**XAC3120**	A0UV6761H03	I.C.4	glucose kinase
**XAC3122**	A0QR6705B12	III.A.1	ATP-dependent RNA helicase
**XAC3123**	A0UT6817G06	III.A.2	DNA-binding related protein
**XAC3124**	A0JJ1536F06	III.A.3	DNA helicase
**XAC3125**	A0UV6736A07	VIII.A	conserved hypothetical protein
**XAC3128**	A0UE6612A02	VIII.B	hypothetical protein
**XAC3129**	A0UV6845G11	III.B.5	pseudouridylate synthase
**XAC3131**	A0UV6762H12	VIII.B	hypothetical protein
**XAC3135**	A0QR6705C11	I.D.1	two-component system, regulatory protein
**XAC3136**	genomic_DNA	I.D.1	two-component system, sensor protein
**XAC3136**	A0AC6407D05	I.D.1	two-component system, sensor protein
**XAC3137**	A0QR6770D01	I.D.2	transcriptional regulator
**XAC3139**	A0UV6515H02	IX	radical activating enzyme
**XAC3140**	A0QR5317H04	VIII.A	conserved hypothetical protein
**XAC3141**	A0EC1090B10	IV.B	outer membrane protein P6 precursor
**XAC3143**	A0JE5637C09	VII.C	TolA protein
**XAC3144**	A0QR6484B08	VII.C	TolR protein
**XAC3145**	A0UV6458D05	VII.C	TolQ protein
**XAC3146**	A0UV6486B07	VIII.A	conserved hypothetical protein
**XAC3147**	A0UV9902A03	III.A.3	holliday junction binding protein, DNA helicase
**XAC3148**	A0UV1501F08	V.A.4	potassium uptake protein
**XAC3149**	A0UV9804A05	III.A.3	holliday junction binding protein, DNA helicase

**XAC3150**	A0QR6002E02	III.A.3	holliday junction resolvase, endodeoxyribonuclease
**XAC3151**	A0QR6704A01	VIII.A	conserved hypothetical protein
**XAC3152**	A0JJ1599G11	VIII.A	conserved hypothetical protein
**XAC3155**	A0QR6460F11	VIII.A	conserved hypothetical protein
**XAC3157**	A0QR6704F03	V.A.1	transmembrane transport protein
**XAC3158**	A0JJ1465G12	V.A.7	TonB-dependent receptor
**XAC3159**	A0UV6711A05	I.B.9	phospholipase C
**XAC3160**	A0CE0105A05	I.B.9	phospholipase C
**XAC3161**	A0UV9880A05	VIII.B	hypothetical protein
**XAC3166**	A0CE6742B02	V.A.4	ferric enterobactin receptor
**XAC3167**	A0QR5407C11	VIII.A	conserved hypothetical protein
**XAC3168**	A0AM1161H10	V.A.4	ferric enterobactin receptor
**XAC3170**	A0QH6836E04	II.D.1	cytochrome P-450 hydroxylase
**XAC3171**	A0AC6824F11	V.A.4	cation:proton antiporter
**XAC3176**	A0UV6739C10	V.A.4	outer membrane receptor; citrate-dependent iron transporter
**XAC3177**	A0AM1452F12	VIII.A	conserved hypothetical protein
**XAC3178**	A0CE6344C10	VIII.A	conserved hypothetical protein
**XAC3179**	A0UV6362C12	V.A.7	transport protein
**XAC3180**	A0QR6002G02	V.A.7	iron transporter
**XAC3181**	A0UT1520D03	II.A.2	diaminopimelate decarboxylase
**XAC3184**	A0UE9854A06	II.D.14	cobalamin synthase
**XAC3185**	A0QR5205H07	VIII.A	conserved hypothetical protein
**XAC3188**	A0RN5611D07	II.D.14	cobyric acid synthase
**XAC3189**	A0AM1365C09	II.D.14	cobalamin biosynthetic protein
**XAC3191**	A0CE6831A05	II.D.14	cob(I)alamin adenolsyltransferase
**XAC3192**	A0UT9845H09	VIII.C	Xanthomonas conserved hypothetical protein
**XAC3193**	A0UV6708C04	VIII.C	Xanthomonas conserved hypothetical protein
**XAC3195**	A0CE9842D07	III.C.3	ATP-dependent Clp protease subunit
**XAC3196**	A0UV9880D09	V.A.7	ABC transporter ATP-binding subunit
**XAC3197**	A0UE9854G12	V.A.7	ABC transporter permease
**XAC3198**	A0QR5902H12	V.A.7	nitrate transport protein
**XAC3199**	A0UV6764B07	I.C.3	oxidoreductase
**XAC3201**	A0CE6743E01	V.A.7	TonB-dependent receptor
**XAC3203**	A0QR5508C08	II.D.10	glutathione transferase
**XAC3204**	A0AC0115H02	VIII.C	Xanthomonas conserved hypothetical protein
**XAC3205**	A0UT9844G06	VIII.A	conserved hypothetical protein
**XAC3206**	A0QR9732C03	VIII.A	conserved hypothetical protein
**XAC3207**	A0QR6389C08	V.A.4	ferric enterobactin receptor
**XAC3211**	A0UV9900B12	VII.G	trehalose-6-phosphate synthase
**XAC3212**	A0AC6824E11	I.C.5	glucose dehydrogenase
**XAC3213**	A0CE9839A04	V.C	chemotaxis protein
**XAC3214**	A0QR5407F12	VIII.A	conserved hypothetical protein
**XAC3216**	A0CE6480C08	VIII.A	conserved hypothetical protein
**XAC3217**	A0QR6839B05	III.B.3	ribosomal large subunit pseudouridine synthase D
**XAC3218**	A0UV9746C10	VII.F	competence lipoprotein
**XAC3219**	A0UV6819G08	VIII.C	Xanthomonas conserved hypothetical protein
**XAC3220**	A0UV6708F05	II.D.7	NH3-dependent NAD synthetase
**XAC3221**	A0UV5502H11	VI.C	ISxac3 transposase
**XAC3223**	A0AR1086E11	VI.C	ISxac3 transposase
**XAC3224**	A0AM1489A12	VII.A	avirulence protein
**XAC3230**	A0UV6844D03	VIII.B	hypothetical protein
**XAC3231**	A0UE6610B09	VIII.B	hypothetical protein
**XAC3233**	A0QH1645G09	VI.C	transposase
**XAC3234**	A0UV6844D07	VIII.B	hypothetical protein

**XAC3236**	A0UV6761B12	I.C.7	succinyl-CoA synthetase, beta subunit
**XAC3237**	A0JE1334D10	I.D.1	two-component system, sensor protein
**XAC3238**	A0QH6810F04	I.D.1	two-component system, regulatory protein
**XAC3239**	A0UV6734H04	IV.D	pilus biogenesis protein
**XAC3240**	A0UT9845G08	IV.D	fimbrillin
**XAC3241**	A0QR5508B06	IV.D	fimbrillin
**XAC3243**	A0QR6460F05	IV.D	type IV pre-pilin leader peptidase
**XAC3244**	A0UE6718B01	VIII.A	conserved hypothetical protein
**XAC3245**	genomic_DNA	IX	RhsD protein
**XAC3245**	A0UV6711H09	IX	RhsD protein
**XAC3247**	A0UV6456H08	VI.C	ISxac3 transposase
**XAC3248**	A0QR6389D09	VI.C	ISxac3 transposase
**XAC3249**	A0QR6704E03	I.D.1	two-component system, sensor protein
**XAC3250**	A0JE1138D03	I.D.1	two-component system, regulatory protein
**XAC3251**	A0EC6476F02	VIII.B	hypothetical protein
**XAC3252**	A0UV6763B11	III.B.3	ribosomal protein S6 modification protein
**XAC3253**	A0UV5526H02	III.B.3	ribosomal protein S6 modification protein
**XAC3254**	A0AC6824D06	I.A.1	glycogen debranching enzyme
**XAC3255**	A0CE6340D09	VIII.B	hypothetical protein
**XAC3256**	A0QR6704D08	VII.H	virulence regulator
**XAC3259**	A0UV6708C03	VIII.B	hypothetical protein
**XAC3260**	A0QR6706B01	VI.B	plasmid mobilization protein
**XAC3261**	A0QH6810D04	VIII.B	hypothetical protein
**XAC3262**	A0QR6838A01	VIII.B	hypothetical protein
**XAC3264**	A0UV6820G11	VIII.B	hypothetical protein
**XAC3267**	A0UV6709C12	VIII.C	Xanthomonas conserved hypothetical protein
**XAC3268**	A0CE6743E07	VIII.B	hypothetical protein
**XAC3269**	A0QR6443E06	III.A.4	RadC family protein
**XAC3270**	A0UE6397D04	VIII.B	hypothetical protein
**XAC3271**	A0UV6766B08	V.C	chemotaxis transducer
**XAC3272**	A0QR6748G06	VIII.A	conserved hypothetical protein
**XAC3273**	A0AC0113B08	I.D.3	histidine kinase/response regulator hybrid protein
**XAC3273**	genomic_DNA	I.D.3	histidine kinase/response regulator hybrid protein
**XAC3274**	A0QH6835B05	I.D.2	single-domain response regulator
**XAC3275**	A0QR6418E09	VIII.B	hypothetical protein
**XAC3276**	A0QR6805E12	VIII.B	hypothetical protein
**XAC3277**	A0UE6463D09	VIII.B	hypothetical protein
**XAC3278**	A0CE9728G11	VIII.B	hypothetical protein
**XAC3279**	A0UV6739D10	VIII.B	hypothetical protein
**XAC3280**	A0QR5323A12	VIII.C	Xanthomonas conserved hypothetical protein
**XAC3281**	A0QH6835E02	VIII.C	Xanthomonas conserved hypothetical protein
**XAC3283**	A0AC6357D06	VI.C	ISxac2 transposase
**XAC3284**	A0QH6309B02	VI.C	ISxac2 transposase
**XAC3285**	genomic_DNA	VIII.B	hypothetical protein
**XAC3285**	genomic_DNA	VIII.B	hypothetical protein
**XAC3287**	A0UV6766B05	VIII.B	hypothetical protein
**XAC3288**	A0QR6366E02	VIII.A	conserved hypothetical protein
**XAC3289**	A0JJ0101E01	VIII.C	Xanthomonas conserved hypothetical protein
**XAC3291**	A0AC6441G04	VIII.C	Xanthomonas conserved hypothetical protein
**XAC3292**	A0AC6823A05	I.D.3	histidine kinase/response regulator hybrid protein
**XAC3293**	A0QR6802C03	VIII.B	hypothetical protein
**XAC3294**	genomic_DNA	VIII.B	hypothetical protein
**XAC3294**	A0QH6382A07	VIII.B	hypothetical protein
**XAC3295**	A0QR6725E12	VIII.B	hypothetical protein

**XAC3298**	A0CE6341E08	VI.A	integrase
**XAC3301**	A0UV9819D11	VIII.A	conserved hypothetical protein
**XAC3303**	A0QR5529G08	III.A.4	DNA mismatch repair protein
**XAC3305**	A0QR5702B01	VIII.A	conserved hypothetical protein
**XAC3306**	A0QR5702H08	VIII.A	conserved hypothetical protein
**XAC3308**	A0QR6483H04	V.A.7	large-conductance mechanosensitive channel
**XAC3309**	A0UE6759C02	III.C.3	aminopeptidase
**XAC3310**	A0QR6729A10	I.D.2	transcriptional regulator lacI family
**XAC3312**	A0JJ1004A08	I.A.1	glycosyl hydrolase
**XAC3314**	A0QR6462H04	VIII.A	conserved hypothetical protein
**XAC3315**	A0JJ1553D03	IX	carboxylesterase
**XAC3316**	A0UV9712H02	III.B.4	tRNA/rRNA methyltransferase
**XAC3317**	A0CE6478A11	III.B.3	acetyltransferase
**XAC3319**	A0UV6487E06	VIII.B	hypothetical protein
**XAC3321**	A0QR6347A10	VI.C	ISxac3 transposase
**XAC3322**	A0QH6836F08	VIII.C	Xanthomonas conserved hypothetical protein
**XAC3323**	A0QR5702D10	IX	acidic amino acid rich protein
**XAC3324**	A0UV6485D07	VIII.C	Xanthomonas conserved hypothetical protein
**XAC3329**	A0UV6764B02	I.B.12	ATP sulfurylase, small subunit
**XAC3330**	A0AM1365H02	I.B.12	NADPH-sulfite reductase, flavoprotein subunit
**XAC3331**	A0UV6711D05	I.B.12	NADPH-sulfite reductase, iron-sulfur protein
**XAC3332**	A0UV6210G07	I.B.12	3'-phosphoadenosine 5'-phosphosulfate reductase
**XAC3333**	A0UV6765A07	VIII.A	conserved hypothetical protein
**XAC3335**	A0AC6823E09	I.D.3	sensor histidine kinase
**XAC3336**	A0JJ0104E07	VIII.C	Xanthomonas conserved hypothetical protein
**XAC3337**	A0EC6474G10	VIII.B	hypothetical protein
**XAC3338**	A0QR5508D03	VIII.C	Xanthomonas conserved hypothetical protein
**XAC3339**	A0UV9805D06	I.D.2	transcriptional regulator lysR family
**XAC3340**	A0QR6413A02	II.D.12	siroheme synthase
**XAC3343**	A0UV6739A05	VIII.A	conserved hypothetical protein
**XAC3344**	A0JJ0102D12	I.C.4	fructose-bisphosphate aldolase
**XAC3347**	A0UE9830F02	I.C.4	phosphoglycerate kinase
**XAC3348**	A0UV6736D07	VIII.A	conserved hypothetical protein
**XAC3352**	A0CE9838C10	I.C.4	glyceraldehyde-3-phosphate dehydrogenase
**XAC3354**	A0CE6740G03	IV.A.2	outer membrane protein W
**XAC3358**	A0QR6375H05	V.A.2	molybdate-binding periplasmic protein; permease
**XAC3360**	A0QR6303H12	V.A.2	ATP-binding component of molybdate transport
**XAC3361**	A0AM0909H04	VIII.C	Xanthomonas conserved hypothetical protein
**XAC3362**	A0QH6731E05	VII.C	TonB-like protein
**XAC3363**	A0UV6820H11	I.D.2	transcriptional regulator blaI family
**XAC3364**	A0QR6730H03	VIII.A	conserved hypothetical protein
**XAC3365**	A0QR6839C01	VIII.A	conserved hypothetical protein
**XAC3368**	A0UV6761D07	VIII.A	conserved hypothetical protein
**XAC3369**	A0UV6734H07	VIII.B	hypothetical protein
**XAC3370**	A0AC6823C04	V.A.4	outer membrane receptor for ferriciron uptake
**XAC3371**	A0AC6824D07	III.C.3	proline imino-peptidase
**XAC3372**	A0UV6764B08	I.B.6	transketolase 1
**XAC3373**	A0UV6486D07	V.A.1	proton glutamate symport protein
**XAC3374**	A0UV6351E09	VIII.A	conserved hypothetical protein
**XAC3375**	A0UV6712F12	VIII.A	conserved hypothetical protein
**XAC3378**	A0UV5307C05	VIII.A	conserved hypothetical protein
**XAC3380**	A0UV9884F02	VIII.B	hypothetical protein
**XAC3381**	A0UV6711A03	IV.D	fimbrial assembly protein
**XAC3383**	A0QR5321F07	IV.D	fimbrial assembly membrane protein

**XAC3385**	A0QR6606F05	IV.D	fimbrial assembly membrane protein
**XAC3388**	A0UV6766B01	I.C.7	citrate synthase
**XAC3389**	A0UV5307A09	III.B.2	50S ribosomal protein L31
**XAC3390**	A0RN1194B11	II.B.4	inosine-uridine preferring nucleoside hydrolase
**XAC3391**	A0QR5904C11	III.A.3	ATP-dependent DNA helicase
**XAC3393**	A0AC6446A01	I.D.4	pentaphosphate guanosine-3'-pyrophosphohydrolase
**XAC3394**	A0UV6819F08	III.B.5	RNA polymerase omega subunit
**XAC3395**	A0UV6733G12	II.B.1	guanylate kinase
**XAC3396**	A0UV9872A02	VIII.A	conserved hypothetical protein
**XAC3398**	A0QR6728D12	VIII.A	conserved hypothetical protein
**XAC3401**	A0UT9846D11	VIII.A	conserved hypothetical protein
**XAC3402**	A0UV9804D09	VIII.A	conserved hypothetical protein
**XAC3403**	A0QR6391F08	III.C.3	proline dipeptidase
**XAC3404**	A0JJ1392B10	VIII.B	hypothetical protein
**XAC3405**	A0RN1116D05	III.C.3	aminopeptidase P
**XAC3406**	A0QR6805G03	VIII.A	conserved hypothetical protein
**XAC3407**	A0QR6804H11	VIII.A	conserved hypothetical protein
**XAC3408**	A0JJ0101F10	VIII.A	conserved hypothetical protein
**XAC3411**	A0UE6464B05	I.B.6	ribose-5-phosphate isomerase A
**XAC3415**	A0AC9896F12	II.D.8	thiamin-phosphate pyrophosphorylase
**XAC3417**	A0QR6460D02	VIII.B	hypothetical protein
**XAC3418**	A0AC0115E05	IV.A.2	Oar protein
**XAC3420**	A0QR9705A06	II.D.12	glutamate-1-semialdehyde 2,1-aminomutase
**XAC3422**	A0QR5704E12	I.C.3	electron transfer protein azurin I
**XAC3423**	A0UV6733A12	II.A.5	histidinol-phosphate aminotransferase
**XAC3424**	A0UV6821A10	IX	TdcF protein
**XAC3425**	A0UV6709G11	I.C.3	cytochrome C6
**XAC3426**	A0AR1086H06	II.D.9	flavin monoamine oxidase-related protein
**XAC3427**	A0UV6843H04	V.A.7	TonB-dependent receptor
**XAC3428**	A0UV6821G12	IX	hydrolase
**XAC3429**	A0JE1209E08	II.A.1	acetylornithine aminotransferase
**XAC3431**	A0UV9903F03	I.C.3	short chain dehydrogenase
**XAC3437**	A0UT9877E06	II.B.1	adenylate kinase
**XAC3438**	A0QR6302H01	I.C.4	6-phosphofructokinase
**XAC3439**	A0QR6839E02	VIII.C	Xanthomonas conserved hypothetical protein
**XAC3440**	A0QR5704H04	I.B.9	H+ translocating pyrophosphate synthase
**XAC3441**	A0QR5904A08	VIII.C	Xanthomonas conserved hypothetical protein
**XAC3443**	A0AC6359C09	I.D.2	response regulator
**XAC3446**	A0EC1468G03	VIII.C	Xanthomonas conserved hypothetical protein
**XAC3448**	A0UV6819G03	V.A.7	TonB-dependent receptor
**XAC3449**	A0UV6708D12	V.C	chemotaxis protein
**XAC3450**	A0JJ1169E10	VII.C	gamma-glutamyltranspeptidase
**XAC3451**	A0QR6704D11	II.A.2	ketol-acid reductoisomerase
**XAC3453**	A0CE6344H01	II.A.2	acetolactate synthase isozyme II, small subunit
**XAC3454**	A0UV9710F07	I.A.2	threonine dehydratase catabolic
**XAC3455**	A0JJ0101D12	II.A.2	2-isopropylmalate synthase
**XAC3457**	A0UV9881F07	II.A.2	3-isopropylmalate dehydratase small subunit
**XAC3458**	A0JE1210D03	II.A.2	3-isopropylmalate dehydratase large subunit
**XAC3459**	A0CE6743G09	I.D.2	transcriptional regulator lysR family
**XAC3460**	A0UV6733H02	VIII.A	conserved hypothetical protein
**XAC3462**	A0UT6814F08	III.C.1	L-isoaspartate protein carboxylmethyltransferase
**XAC3463**	A0UV6739A12	VII.C	TolC protein
**XAC3464**	A0CE6507B09	IV.C	3-deoxy-D-manno-octulosonic acid transferase
**XAC3467**	A0EC1093E08	IX	glycosyltransferase

**XAC3468**	A0UV6766D03	VIII.A	conserved hypothetical protein
**XAC3473**	A0UV6821D09	I.D.3	sensor histidine kinase
**XAC3476**	A0AC9895B11	I.D.2	transcriptional regulator
**XAC3477**	A0RN1196E12	I.C.3	rhizopine catabolism protein mocA
**XAC3479**	A0UV6351E06	VIII.A	conserved hypothetical protein
**XAC3480**	A0AM1594B03	I.D.2	transcriptional regulator luxR/uhpA family
**XAC3486**	A0JE1208C11	II.E	acetoacetyl-CoA reductase
**XAC3487**	A0JJ1442E11	I.D.2	transcriptional regulator
**XAC3490**	A0QR6804D02	III.D.1	amylosucrase or alpha amylase
**XAC3491**	A0UV6734A08	VII.C	NonF-related protein
**XAC3492**	A0AC0113H05	VIII.A	conserved hypothetical protein
**XAC3495**	A0AM1318C01	VIII.A	conserved hypothetical protein
**XAC3496**	A0UV6350A12	VIII.C	Xanthomonas conserved hypothetical protein
**XAC3497**	A0JE5637A08	VIII.B	hypothetical protein
**XAC3498**	A0JJ1577H11	V.A.4	outer membrane receptor for ferric iron uptake
**XAC3500**	A0UV6733G11	II.D.10	glutaredoxin-like protein
**XAC3501**	A0CE6507E11	VIII.C	Xanthomonas conserved hypothetical protein
**XAC3502**	A0UV6819D06	VIII.A	conserved hypothetical protein
**XAC3505**	A0QR6804D04	I.A.1	rhamnogalacturonase B
**XAC3506**	A0UV6734H11	VII.D	cellulase S (truncated)
**XAC3507**	A0JJ1001G02	VII.D	cellulase S (truncated)
**XAC3508**	A0AM1489A01	VIII.A	conserved hypothetical protein
**XAC3511**	A0QR6417F12	VIII.B	hypothetical protein
**XAC3512**	A0CE9842H05	VII.C	arsenate reductase
**XAC3514**	A0AM1491D12	III.C.3	serine protease
**XAC3516**	A0QR6802E03	VII.D	cellulase
**XAC3517**	A0QR5904H11	VIII.A	conserved hypothetical protein
**XAC3518**	A0EC6427B05	III.D.1	celullose synthase
**XAC3519**	A0UV6765B02	VIII.B	hypothetical protein
**XAC3521**	A0QR6437A01	II.D.7	nicotinate phosphoribosyltransferase
**XAC3523**	A0AM1309C07	VIII.C	Xanthomonas conserved hypothetical protein
**XAC3525**	A0QR6204F07	VIII.A	conserved hypothetical protein
**XAC3529**	A0UV6348E01	V.A.7	iron receptor
**XAC3530**	A0JE1044B04	V.A.7	iron receptor
**XAC3531**	A0UE6718H06	V.A.5	PnuC protein
**XAC3536**	A0QR6388H06	VII.H	general secretion pathway protein M
**XAC3538**	A0UV6765B09	VII.H	general secretion pathway protein K
**XAC3539**	A0JJ1072B03	VII.H	general secretion pathway protein J
**XAC3540**	A0UV6709D02	VII.H	general secretion pathway protein I
**XAC3541**	A0UV6709B05	VII.H	general secretion pathway protein H
**XAC3542**	A0EC1091E04	VII.H	general secretion pathway protein G
**XAC3543**	A0AM1404D09	VII.H	general secretion pathway protein F
**XAC3545**	A0UV6485C05	III.C.3	protease
**XAC3546**	A0QR6728H08	VII.F	outer membrane protein
**XAC3547**	A0QR1369H12	III.C.3	serine protease
**XAC3548**	A0AC1317A05	VII.F	outer membrane protein
**XAC3550**	A0QR5704G06	III.C.1	disulfide isomerase
**XAC3551**	A0UV6761A08	III.A.3	integrase/recombinase XerD
**XAC3553**	A0QH6810H12	VIII.B	hypothetical protein
**XAC3555**	A0UV6514C04	VIII.A	conserved hypothetical protein
**XAC3556**	A0JJ0101G09	III.C.3	aminopeptidase A/I
**XAC3557**	A0QH6412B10	VIII.B	hypothetical protein
**XAC3558**	A0UV6761H11	III.A.1	DNA polymerase III holoenzyme chi subunit
**XAC3560**	A0QH1645B04	V.A.7	TonB-dependent receptor

**XAC3561**	A0AM1309B03	IV.B	soluble lytic murein transglycosylase
**XAC3565**	A0QR5702C10	III.D.2	phosphatidylserine synthase
**XAC3566**	A0EC6475E09	VIII.A	conserved hypothetical protein
**XAC3573**	A0CE0105A06	VIII.A	conserved hypothetical protein
**XAC3574**	A0JE1044B02	III.A.4	DNA repair system specific for alkylated DNA
**XAC3575**	A0QR6392H04	I.C.3	flavoprotein-ubiquinone oxidoreductase
**XAC3576**	A0JJ1329D09	VIII.A	conserved hypothetical protein
**XAC3578**	A0UV6734C08	IV.A.1	IpsJ protein
**XAC3579**	A0UV6709B03	VII.E	phosphoglucomutase/phosphomannomutase
**XAC3581**	A0CE6742A11	I.B.11	UDP-glucose dehydrogenase
**XAC3583**	A0CE6828B08	IV.C	dTDP-4-dehydrorhamnose 3,5-epimerase
**XAC3584**	A0UT6814G10	IV.A.1	glucose-1-phosphate thymidylyltransferase
**XAC3585**	A0QR6803G01	IV.C	dTDP-glucose 4,6-dehydratase
**XAC3587**	A0AC6494F09	I.C.3	electron transfer flavoprotein alpha subunit
**XAC3589**	A0UV6712E09	IV.A.1	integral membrane protein
**XAC3590**	A0CE6829A08	I.C.3	oxidoreductase
**XAC3591**	A0AC6827F01	I.C.3	short chain dehydrogenase
**XAC3594**	A0QR6729G10	II.D.17	phytoene desaturase
**XAC3598**	A0UT9800E08	IV.C	O-antigen biosynthesis protein (truncated)
**XAC3599**	A0CE6828B01	VIII.B	hypothetical protein
**XAC3601**	A0QR6727G10	V.A.7	ABC transporter permease
**XAC3603**	A0CE9839F11	II.A.3	cystathionine beta-synthase
**XAC3605**	A0UV6711A09	IV.A.2	outer membrane protein
**XAC3607**	A0UV6845C08	IV.A.2	type II secretion system protein-like protein
**XAC3607**	A0QR9753D06	IV.A.2	type II secretion system protein-like protein
**XAC3610**	A0QR5407C09	III.B.5	ATP-dependent RNA helicase
**XAC3612**	A0QR6749B04	IX	peptidase
**XAC3613**	A0UV6738F07	V.A.7	TonB-dependent receptor
**XAC3617**	A0EC6473G06	VIII.A	conserved hypothetical protein
**XAC3618**	A0UE6759F06	VIII.A	conserved hypothetical protein
**XAC3619**	A0AC6449D12	VIII.A	conserved hypothetical protein
**XAC3620**	A0JJ0101B11	V.A.7	siderophore receptor protein
**XAC3623**	A0UV6514G03	II.E	beta-hydroxydecanoyl-ACP dehydratase
**XAC3625**	A0UV6762H08	II.E	beta-ketoacyl-[ACP] synthase I
**XAC3626**	A0QR5703C09	VIII.B	hypothetical protein
**XAC3627**	A0QR9812E12	III.C.3	oligopeptidase A
**XAC3628**	A0UT9875H06	II.A.3	cysteine synthase
**XAC3630**	A0UV6738A05	VII.C	copper resistance protein A precursor
**XAC3632**	A0CE6740H08	I.B.10	lactoylglutathione lyase
**XAC3634**	A0UV6844G04	VIII.A	conserved hypothetical protein
**XAC3636**	A0QR6750A12	VIII.C	Xanthomonas conserved hypothetical protein
**XAC3641**	A0CE6746C05	V.A.7	ABC transporter permease
**XAC3643**	A0JJ1015G05	I.D.3	histidine kinase/response regulator hybrid protein
**XAC3645**	A0QR6705E12	VIII.C	Xanthomonas conserved hypothetical protein
**XAC3647**	A0UT6817D11	II.A.4	chorismate mutase/prephenate dehydratase
**XAC3648**	A0QR6804H10	I.C.8	ATP synthase, epsilon chain
**XAC3649**	A0CE0105B09	I.C.8	ATP synthase, beta chain
**XAC3650**	A0UV6707F08	I.C.8	ATP synthase, gamma chain
**XAC3651**	A0UV6818F05	I.C.8	ATP synthase, alpha chain
**XAC3653**	A0UV6736G02	I.C.8	ATP synthase, B chain
**XAC3654**	A0UE6759C03	I.C.8	ATP synthase, C chain
**XAC3655**	A0JE1211A02	I.C.8	ATP synthase, A chain
**XAC3657**	A0QH6811G03	VIII.C	Xanthomonas conserved hypothetical protein
**XAC3658**	A0AC9895E08	VIII.B	hypothetical protein

**XAC3659**	A0QR6768E09	I.C.6	dihydrolipoamide dehydrogenase
**XAC3661**	A0QH6382D04	I.C.6	dihydrolipoamide acetyltranferase
**XAC3662**	A0EC6427H05	VIII.A	conserved hypothetical protein
**XAC3664**	A0CE6828B06	IV.A.2	outer membrane protein
**XAC3665**	A0CE9837G04	VIII.C	Xanthomonas conserved hypothetical protein
**XAC3667**	A0UV6351B09	IV.A.2	outer membrane protein
**XAC3668**	A0UV9711C06	V.A.7	ABC transporter permease
**XAC3669**	A0UV6766C05	V.A.7	ABC transporter ATP-binding protein
**XAC3670**	A0AM1617C01	VIII.A	conserved hypothetical protein
**XAC3672**	A0QR6748H10	VIII.B	hypothetical protein
**XAC3673**	A0AC6823F01	I.D.3	histidine kinase
**XAC3674**	A0CE6341C02	VIII.A	conserved hypothetical protein
**XAC3676**	A0UV6763A04	I.C.3	oxidoreductase
**XAC3677**	A0AC6431A04	IX	conserved hypothetical protein
**XAC3678**	A0UV6738H02	III.B.3	ribosomal small subunit pseudouridylate synthase
**XAC3679**	A0UV6846F03	III.B.3	ribosomal RNA small subunit methyltransferase C
**XAC3680**	A0QH6810A12	VIII.C	Xanthomonas conserved hypothetical protein
**XAC3681**	A0QR6705F08	I.A.2	L-sorbosone dehydrogenase
**XAC3682**	A0QR6730A03	VIII.C	Xanthomonas conserved hypothetical protein
**XAC3683**	A0UV6708B12	I.D.3	sensor histidine kinase
**XAC3684**	A0QH6810G02	VIII.C	Xanthomonas conserved hypothetical protein
**XAC3685**	A0UV6766F06	VIII.C	Xanthomonas conserved hypothetical protein
**XAC3689**	A0JE5637H08	I.D.2	leucine responsive regulatory protein
**XAC3691**	A0AR1008E04	VIII.A	conserved hypothetical protein
**XAC3692**	A0UV9748E09	VIII.A	conserved hypothetical protein
**XAC3693**	A0QR6730E11	V.C	chemotaxis protein
**XAC3694**	A0QH9865A02	V.C	chemotaxis MotB protein
**XAC3696**	A0UV6363B08	VIII.A	conserved hypothetical protein
**XAC3699**	A0UV6434E09	V.A.7	ABC-2 type transporter
**XAC3700**	A0QR5902C04	V.A.1	ABC transporter ATP-binding protein
**XAC3701**	A0UV6514C09	V.A.4	Na+:H+ antiporter
**XAC3702**	A0UV6761F09	VIII.B	hypothetical protein
**XAC3703**	A0UV9709A04	VIII.A	conserved hypothetical protein
**XAC3705**	A0UV9710G08	VIII.A	conserved hypothetical protein
**XAC3707**	A0QR6770B09	VIII.A	conserved hypothetical protein
**XAC3708**	A0QR6002D08	I.D.2	transcriptional regulator
**XAC3709**	A0UV6708G05	I.D.2	tryptophan repressor binding protein
**XAC3711**	A0QR6804G05	VIII.A	conserved hypothetical protein
**XAC3712**	A0JJ1577F08	III.C.3	metallopeptidase
**XAC3713**	A0CE9842A06	III.C.3	peptidase
**XAC3714**	A0UV6206D02	VIII.A	conserved hypothetical protein
**XAC3716**	A0QH6731H06	VIII.B	hypothetical protein
**XAC3718**	A0UV6736C04	VIII.C	Xanthomonas conserved hypothetical protein
**XAC3719**	A0AM0909B02	I.D.2	transcriptional regulator protein
**XAC3720**	A0UT6815C04	VIII.A	conserved hypothetical protein
**XAC3721**	A0UV6739C03	I.A.2	D-amino acid oxidase
**XAC3723**	A0AR1102A02	VIII.B	hypothetical protein
**XAC3723**	genomic_DNA	VIII.B	hypothetical protein
**XAC3724**	A0UV6710F02	VIII.C	Xanthomonas conserved hypothetical protein
**XAC3726**	A0QR6489E03	VIII.A	conserved hypothetical protein
**XAC3728**	A0UV6712E06	VIII.C	Xanthomonas conserved hypothetical protein
**XAC3732**	A0QR6203E09	VIII.C	Xanthomonas conserved hypothetical protein
**XAC3734**	A0UV6761H09	VIII.C	Xanthomonas conserved hypothetical protein
**XAC3735**	A0QR6770C03	I.C.3	cyanide insensitive terminal oxidase

**XAC3736**	A0QH6810B01	I.C.3	cyanide insensitive terminal oxidase
**XAC3739**	A0UV6711H08	VIII.A	conserved hypothetical protein
**XAC3740**	A0CE9723F10	I.A.2	UDP-glucose 4-epimerase
**XAC3741**	A0QR6730E05	VIII.A	conserved hypothetical protein
**XAC3742**	A0JE1331H08	IV.C	UDP-galactopyranose mutase
**XAC3743**	A0CE6830H01	VIII.A	conserved hypothetical protein
**XAC3744**	A0QR6804D07	V.A.7	ATP binding transporter 1
**XAC3747**	A0UV6710E07	I.C.3	alcohol dehydrogenase (Zn-dependent)
**XAC3751**	A0QR9767E08	VIII.C	Xanthomonas conserved hypothetical protein
**XAC3752**	A0UV6739G05	VIII.C	Xanthomonas conserved hypothetical protein
**XAC3753**	A0QR5520D04	VIII.A	conserved hypothetical protein
**XAC3755**	A0QR6802A08	VIII.C	Xanthomonas conserved hypothetical protein
**XAC3757**	A0AM1542C08	VIII.A	conserved hypothetical protein
**XAC3759**	A0QR5701C12	V.A.7	ABC transporter ATP-binding protein
**XAC3762**	A0UT6817B06	VIII.A	conserved hypothetical protein
**XAC3764**	A0UT1515C10	VI.C	ISxac2 transposase
**XAC3766**	A0UV6846C05	VIII.B	hypothetical protein
**XAC3769**	A0QR5327H09	III.A.5	endonuclease precursor
**XAC3770**	A0UV6348F07	VIII.A	conserved hypothetical protein
**XAC3775**	A0UV9746D12	VIII.B	hypothetical protein
**XAC3779**	A0QR9814H10	VIII.B	hypothetical protein
**XAC3780**	A0AC6825E10	V.A.7	chloride channel
**XAC3784**	A0QH6412H03	VIII.B	hypothetical protein
**XAC3788**	genomic_DNA	I.D.4	RNA polymerase sigma-70 factor
**XAC3789**	A0CE6743B01	VIII.A	conserved hypothetical protein
**XAC3792**	A0QH6732C05	II.D.9	riboflavin biosynthesis protein
**XAC3794**	A0UE9858C06	VIII.A	conserved hypothetical protein
**XAC3795**	A0UV6733A05	VIII.A	conserved hypothetical protein
**XAC3798**	A0QR6752G11	VIII.A	conserved hypothetical protein
**XAC3799**	A0QR5704B09	IX	Sun protein
**XAC3800**	A0CE6743C01	III.B.4	10-Formyltetrahydrofolate:L-methionyl-tRNA(fMet) N-formyltransferase
**XAC3802**	A0UV9805F05	VIII.A	conserved hypothetical protein
**XAC3803**	A0QR6729G09	III.A.5	DNA processing chain A
**XAC3804**	A0UV6708B02	IX	Smg protein
**XAC3806**	A0UE6759C01	VIII.A	conserved hypothetical protein
**XAC3807**	A0UV6846E01	III.A.1	DNA topoisomerase I
**XAC3808**	A0JE1333F09	VIII.A	conserved hypothetical protein
**XAC3810**	A0CE6743D02	VIII.C	Xanthomonas conserved hypothetical protein
**XAC3811**	A0UV9872C07	I.B.10	tropinone reductase
**XAC3813**	A0UV6206H02	III.C.3	protease IV
**XAC3814**	A0QR5311D08	VII.C	multidrug efflux protein
**XAC3815**	A0UV6208H01	VIII.A	conserved hypothetical protein
**XAC3816**	A0QR5529E02	VIII.A	conserved hypothetical protein
**XAC3817**	A0QR6334A05	VIII.A	conserved hypothetical protein
**XAC3818**	A0UV6845H07	III.A.1	primosomal protein N'
**XAC3822**	A0JJ1656C06	I.C.1	NADH dehydrogenase
**XAC3823**	A0UE6464E04	IV.A.1	conserved hypothetical protein
**XAC3824**	genomic_DNA	I.D.4	RNA polymerase sigma-32 factor
**XAC3824**	A0QR5520F08	I.D.4	RNA polymerase sigma-32 factor
**XAC3825**	A0UV6709G02	III.A.4	uracil-DNA glycosylase
**XAC3826**	A0UV6735H06	IX	response regulator protein
**XAC3828**	A0EC6322H04	V.A.7	ABC transporter ATP-binding protein
**XAC3830**	A0QR6705D10	II.D.10	thioredoxin
**XAC3831**	A0QR9753H08	III.B.5	transcription termination factor Rho

**XAC3833**	A0UV9819D07	I.D.2	B-lactamase regulatory protein
**XAC3836**	A0QR5702D06	VIII.A	conserved hypothetical protein
**XAC3838**	A0QR6752A05	VIII.A	conserved hypothetical protein
**XAC3840**	A0CE6345D09	VIII.A	conserved hypothetical protein
**XAC3841**	A0UV9900C01	VIII.A	conserved hypothetical protein
**XAC3843**	A0QR5701B02	VIII.A	conserved hypothetical protein
**XAC3844**	A0QR5206A02	VIII.C	Xanthomonas conserved hypothetical protein
**XAC3846**	A0UV9709G03	VIII.A	conserved hypothetical protein
**XAC3847**	A0QR5518B04	III.C.3	N-acyl-L-amino acid amidohydrolase
**XAC3848**	A0QR6840E05	VII.C	membrane fusion protein precursor
**XAC3849**	A0RN1627D03	VII.C	acriflavin resistance protein
**XAC3850**	A0CE6743G05	VII.C	acriflavin resistance protein
**XAC3851**	A0QR6204C10	VIII.A	conserved hypothetical protein
**XAC3852**	A0UE6718H08	VIII.A	conserved hypothetical protein
**XAC3854**	A0AC6827A05	I.A.2	hydrolase, haloacid delahogenase-like family
**XAC3857**	A0QR6702A05	VIII.C	Xanthomonas conserved hypothetical protein
**XAC3859**	A0CE6743F06	III.C.3	D-alanyl-D-alanine dipeptidase
**XAC3860**	A0QR5407H10	IV.B	N-acetylmuramoyl-L-alanine amidase
**XAC3861**	A0UV6819C06	VIII.A	conserved hypothetical protein
**XAC3862**	A0UV6709A02	I.A.2	chloromuconate cycloisomerase
**XAC3863**	A0CE6742G04	VIII.A	conserved hypothetical protein
**XAC3864**	A0AC9763E09	V.A.4	cationic amino acid transporter
**XAC3865**	A0CE6831D06	VIII.A	conserved hypothetical protein
**XAC3866**	A0UE9721H09	VIII.C	Xanthomonas conserved hypothetical protein
**XAC3867**	A0UV9904B06	IV.A.1	membrane protein
**XAC3868**	A0CE0105C06	I.C.3	dehydrogenase
**XAC3869**	A0JE1079C03	I.A.1	beta-glucosidase
**XAC3870**	A0CE6740E06	II.D.2	dihydroneopterin aldolase
**XAC3871**	A0QR6303E04	III.C.3	O-sialoglycoprotein endopeptidase
**XAC3873**	A0UV6819A06	VIII.A	conserved hypothetical protein
**XAC3874**	A0CE6743C04	VIII.B	hypothetical protein
**XAC3875**	A0QR6614E04	III.B.4	ribonuclease BN
**XAC3878**	A0UV9879H12	IX	disulphide-isomerase
**XAC3879**	A0CE6341E05	I.C.3	cytochrome C oxidase assembly factor
**XAC3882**	A0AC6358D06	VIII.A	conserved hypothetical protein
**XAC3883**	A0UV6207H05	VIII.A	conserved hypothetical protein
**XAC3884**	A0QH6306C09	I.C.1	cytochrome C oxidase, polypeptide III
**XAC3888**	A0UT6815A09	I.C.1	cytochrome C oxidase, polypeptide II
**XAC3890**	A0QH6309G08	I.A.2	bifunctional PutA protein
**XAC3891**	A0CE6829E08	VIII.B	hypothetical protein
**XAC3898**	A0UE9829D08	VIII.A	conserved hypothetical protein
**XAC3900**	A0UV6820D09	VIII.C	Xanthomonas conserved hypothetical protein
**XAC3902**	A0UE6718A09	III.A.4	exodeoxyribonuclease III
**XAC3903**	A0AC1383E07	II.B.2	orotate phosphoribosyl transferase
**XAC3904**	A0JJ1167E03	VIII.A	conserved hypothetical protein
**XAC3906**	A0QR6369G12	V.B	chromosome partitioning protein
**XAC3907**	A0UV9822F04	VII.C	mitomycin resistance protein
**XAC3909**	A0UV5526C09	III.D.1	dolichol-phosphate mannosyltransferase
**XAC3911**	A0QH6731D04	VIII.C	Xanthomonas conserved hypothetical protein
**XAC3912**	A0QR6730D09	IV.A.1	phosphomannomutase
**XAC3913**	A0UV6712G12	II.B.4	dUTPase
**XAC3914**	A0CE6831F09	III.A.1	DNA/pantothenate metabolism flavoprotein
**XAC3918**	A0UV9822F10	VIII.C	Xanthomonas conserved hypothetical protein
**XAC3921**	A0UT9845G11	IX	glucosyltransferase

**XAC3922**	A0QR6840G09	II.D.15	ATP-dependent serine activating enzyme
**XAC3925**	A0QH6307G05	VIII.A	conserved hypothetical protein
**XAC3929**	A0UV6709B06	I.D.2	nitrogen regulatory protein P-II
**XAC3931**	A0CE6341D10	VII.F	competence related protein
**XAC3932**	A0QR6613A10	VI.C	integrase/recombinase
**XAC3933**	A0CE9726D08	VIII.A	conserved hypothetical protein
**XAC3935**	A0CE6740F04	VI.C	IS1389 transposase
**XAC3936**	A0UV6846E09	VI.C	IS1389 transposase
**XAC3938**	A0UE9858A03	VI.C	ISxac3 transposase
**XAC3941**	A0CE6829B07	III.A.4	DNA helicase
**XAC3946**	A0UE6757F04	VIII.B	hypothetical protein
**XAC3948**	A0JJ1002D08	VIII.A	conserved hypothetical protein
**XAC3950**	A0UV6736H08	VIII.A	conserved hypothetical protein
**XAC3951**	A0QR6601F08	VIII.B	hypothetical protein
**XAC3952**	A0JJ0705F03	VIII.A	conserved hypothetical protein
**XAC3954**	A0AC6430D04	VIII.B	hypothetical protein
**XAC3956**	A0UV6739F12	III.D.3	outer membrane lipoprotein Blc
**XAC3957**	A0UT6387C05	VIII.C	Xanthomonas conserved hypothetical protein
**XAC3959**	A0UV6763A08	VIII.A	conserved hypothetical protein
**XAC3960**	A0QR5904H09	I.C.3	oxidoreductase
**XAC3961**	A0UV6434F04	I.D.2	transcriptional regulator tetR family
**XAC3962**	A0UV1534F12	VIII.B	hypothetical protein
**XAC3965**	A0QR6304B05	VIII.A	conserved hypothetical protein
**XAC3966**	A0UV6765F01	VIII.C	Xanthomonas conserved hypothetical protein
**XAC3970**	A0QR6443G07	VIII.C	Xanthomonas conserved hypothetical protein
**XAC3971**	A0QR9718A03	VIII.C	Xanthomonas conserved hypothetical protein
**XAC3972**	A0UV6762G08	VIII.A	conserved hypothetical protein
**XAC3973**	A0CE6506D09	V.B	cell division inhibitor
**XAC3975**	A0QR6727F12	I.D.1	two-component system, sensor protein
**XAC3976**	A0UT6815C07	VIII.C	Xanthomonas conserved hypothetical protein
**XAC3977**	A0UV6710D04	VIII.C	Xanthomonas conserved hypothetical protein
**XAC3982**	A0QR5903D09	VIII.A	conserved hypothetical protein
**XAC3983**	A0CE6746A11	VIII.A	conserved hypothetical protein
**XAC3984**	genomic_DNA	VIII.B	hypothetical protein
**XAC3985**	A0CE9724E02	V.A.7	transport protein
**XAC3986**	A0UV6710F07	IX	hydrolase
**XAC3987**	A0QH6810F05	III.C.3	leucine aminopeptidase
**XAC3988**	A0UV6351H04	I.D.2	transmembrane regulator protein prtR
**XAC3989**	A0UV6736A01	I.D.4	ECF sigma factor
**XAC3991**	A0QR6728D01	I.C.3	cytochrome B561
**XAC3993**	A0UV6712D02	I.D.1	two-component system, regulatory protein
**XAC3995**	A0QH6810G09	VII.C	acriflavin resistance protein
**XAC3996**	A0QR6737A06	V.A.7	ABC transporter ATP-binding protein
**XAC3998**	A0UV6709G03	VIII.A	conserved hypothetical protein
**XAC4001**	A0CE9725C03	IV.A.1	integral membrane protein
**XAC4002**	A0QR6729C08	VIII.A	conserved hypothetical protein
**XAC4004**	A0UV5515G12	III.C.3	peptidase
**XAC4005**	A0QR5704G03	VII.C	beta-lactamase related protein
**XAC4006**	A0UV6819D11	III.B.4	tryptophanyl-tRNA synthetase
**XAC4008**	A0AC6824G12	VII.C	entericidin A
**XAC4009**	A0UV6846B01	I.A.2	arginase
**XAC4012**	A0JJ1311A05	VIII.C	Xanthomonas conserved hypothetical protein
**XAC4013**	A0QR5518E12	VIII.A	conserved hypothetical protein
**XAC4016**	A0AC0115F09	VIII.A	conserved hypothetical protein

**XAC4017**	A0QR6490H06	I.D.2	transcriptional regulator
**XAC4018**	A0UV5502C12	I.D.2	bifunctional transcriptional repressor of the biotin operon/biotin acetyl-CoA-carboxylase synthetase
**XAC4019**	A0QR6705D05	VIII.A	conserved hypothetical protein
**XAC4020**	A0CE6740D01	VIII.A	conserved hypothetical protein
**XAC4021**	A0UV9870E01	VIII.A	conserved hypothetical protein
**XAC4022**	A0AC0115B07	I.D.1	two-component system, sensor protein
**XAC4023**	A0AC6825G01	I.D.1	two-component system, regulatory protein
**XAC4027**	A0QR6729E07	VIII.B	hypothetical protein
**XAC4028**	A0CE5319F01	IV.A.1	ankyrin like protein
**XAC4029**	A0UV6766G10	VII.C	catalase [precursor]
**XAC4030**	A0QR5702D01	VII.C	catalase
**XAC4031**	A0UV6456E02	III.A.4	ATP-dependent helicase
**XAC4032**	A0QR6728E11	VIII.C	Xanthomonas conserved hypothetical protein
**XAC4033**	A0CE6740D02	VIII.C	Xanthomonas conserved hypothetical protein
**XAC4035**	A0CE9837F11	VIII.C	Xanthomonas conserved hypothetical protein
**XAC4037**	A0AC0113B07	III.A.5	endonuclease
**XAC4039**	A0UV6766A02	VIII.A	conserved hypothetical protein
**XAC4040**	A0UV6433E07	II.D.12	delta-aminolevulinic acid dehydratase
**XAC4044**	A0CE6508A11	VIII.A	conserved hypothetical protein
**XAC4045**	A0UE6718G10	VIII.B	hypothetical protein
**XAC4046**	A0UE6758A07	III.C.3	dipeptidyl peptidase IV
**XAC4047**	A0UV6708F09	VII.C	glutathione S-transferase
**XAC4048**	A0QR6725G09	V.A.7	TonB-dependent receptor
**XAC4049**	A0UT9800D12	IV.A.2	SapC protein
**XAC4050**	A0QR6705E08	IX	Pass1-related protein
**XAC4051**	A0AC6824A06	II.D.17	tryptophan halogenase
**XAC4052**	A0QR6613C10	VII.C	TonB-like protein
**XAC4053**	A0UV6819C04	V.A.4	ABC transporter sodium permease
**XAC4054**	A0UV6733H12	V.A.4	sodium ABC transporter ATP-binding protein
**XAC4055**	A0QR9705E12	III.C.3	cysteine proteinase
**XAC4057**	A0QR6803D01	VIII.A	conserved hypothetical protein
**XAC4058**	A0AC0113D07	I.A.1	beta-xylosidase
**XAC4059**	A0UE6759H12	VIII.A	conserved hypothetical protein
**XAC4060**	A0CE6740G01	V.A.7	heavy metal transporter
**XAC4061**	A0UT6814D02	VIII.A	conserved hypothetical protein
**XAC4062**	A0QR5529A02	V.A.7	TonB-dependent receptor
**XAC4064**	A0UE9890E10	I.D.2	transcriptional regulator araC family
**XAC4065**	A0UV6736A08	V.A.7	ABC transporter ATP-binding protein
**XAC4066**	A0QR6840F06	I.A.2	phenol hydroxylase
**XAC4067**	A0QR6330A02	V.A.2	permease
**XAC4068**	A0QR6729E04	II.B.2	2-dehydropantoate 2-reductase
**XAC4071**	A0UV6734C10	VIII.B	hypothetical protein
**XAC4072**	A0QH6810E08	II.D.10	thioredoxin
**XAC4073**	A0CE5309D04	VIII.A	conserved hypothetical protein
**XAC4075**	A0AC6827C10	II.B.3	ribonucleoside-diphosphate reductase alpha chain
**XAC4076**	A0UV9819G09	IV.A.1	integral membrane protein
**XAC4077**	A0UV6733F10	VIII.A	conserved hypothetical protein
**XAC4079**	A0UV9870E11	I.B.10	a-type carbonic anhydrase
**XAC4082**	A0QH6836E02	VIII.A	conserved hypothetical protein
**XAC4085**	A0QR6839A11	VIII.A	conserved hypothetical protein
**XAC4086**	A0UV5307B04	II.E	beta-ketoacyl-[ACP] synthase II
**XAC4087**	A0UV9870C07	VIII.A	conserved hypothetical protein
**XAC4089**	A0UE6463C09	II.D.17	halogenase

**XAC4091**	A0CE9723A11	VIII.B	hypothetical protein
**XAC4094**	A0QR6768F01	IV.A.1	membrane protein
**XAC4095**	A0UV9905C04	VIII.A	conserved hypothetical protein
**XAC4096**	A0QR6730E10	II.E	fatty acyl CoA synthetase
**XAC4099**	A0QR5205D08	III.D.2	acyltransferase
**XAC4101**	A0UE6759G07	II.E	acyl carrier protein
**XAC4102**	genomic_DNA	II.D.17	hydroxylase
**XAC4105**	A0RN1632B02	VII.C	AMP-ligase
**XAC4106**	A0RN1457G01	III.C.3	dipeptidyl peptidase
**XAC4107**	A0UV6763G08	VIII.C	Xanthomonas conserved hypothetical protein
**XAC4108**	A0JJ1304G04	VIII.A	conserved hypothetical protein
**XAC4109**	A0QH6306E03	II.D.12	coproporphyrinogen III oxidase, aerobic
**XAC4111**	A0UV6739F07	VIII.A	conserved hypothetical protein
**XAC4118**	A0UT9891E01	VIII.A	conserved hypothetical protein
**XAC4119**	A0AC0113B06	VIII.A	conserved hypothetical protein
**XAC4121**	A0AM1111C09	VIII.A	conserved hypothetical protein
**XAC4122**	A0CE5319B12	VIII.A	conserved hypothetical protein
**XAC4124**	A0UV6708E07	VIII.A	conserved hypothetical protein
**XAC4127**	A0QH6732E02	I.D.3	serine/threonine kinase
**XAC4128**	genomic_DNA	I.D.3	extracytoplasmic sigma factor
**XAC4128**	A0QH6713B12	I.D.3	extracytoplasmic sigma factor
**XAC4129**	genomic_DNA	I.D.4	ECF sigma factor
**XAC4129**	A0QR5902H04	I.D.4	ECF sigma factor
**XAC4131**	A0QR6377E09	VIII.A	conserved hypothetical protein
**XAC4133**	A0CE6743E02	VIII.A	conserved hypothetical protein
**XAC4137**	A0AR1102A01	VI.C	ISxac1 transposase
**XAC4139**	A0UV6736E01	VIII.B	hypothetical protein
**XAC4140**	A0QR5904F09	III.C.2	ClpB
**XAC4142**	A0UV6710D09	VIII.A	conserved hypothetical protein
**XAC4145**	A0QR5701A01	VIII.A	conserved hypothetical protein
**XAC4146**	A0QR5702C01	VIII.A	conserved hypothetical protein
**XAC4147**	A0UV6735E04	VIII.A	conserved hypothetical protein
**XAC4148**	A0EC0204F05	I.D.2	transcriptional regulator
**XAC4150**	A0UV6845F05	IV.C	nodulation protein
**XAC4154**	A0QR9756C01	I.D.2	transcriptional regulator
**XAC4156**	A0UV9880G11	I.C.3	FldA protein
**XAC4157**	A0QR6804D05	I.A.2	4-oxalomesaconate hydratase
**XAC4158**	A0QH6836C02	III.B.2	50S ribosomal protein L33
**XAC4159**	A0QR5902D09	III.B.2	50S ribosomal protein L28
**XAC4160**	A0QR5322D03	VII.C	cation efflux system protein
**XAC4161**	A0QR6203D08	VII.C	cation efflux system protein
**XAC4162**	A0AM1490C05	VII.C	cation efflux system protein
**XAC4165**	A0UV9805C01	III.A.1	glucose inhibited division protein B
**XAC4166**	A0UT5316B08	I.B.9	alkaline phosphatase D
**XAC4170**	A0QH9737E12	VIII.A	conserved hypothetical protein
**XAC4171**	A0UV6436A12	III.A.4	exodeoxyribonuclease III
**XAC4173**	A0UV9900C07	VIII.C	Xanthomonas conserved hypothetical protein
**XAC4176**	A0UV6739A01	V.A.7	solute:Na+ symporter
**XAC4179**	A0QR5902H10	II.E	acetyl coenzyme A synthetase
**XAC4180**	A0QR6770F02	I.D.1	two-component system, regulatory protein
**XAC4181**	A0UV9904C05	VIII.A	conserved hypothetical protein
**XAC4183**	A0UV6709B07	I.A.1	xylosidase
**XAC4186**	A0CE6345D12	I.C.3	oxidoreductase
**XAC4187**	A0JE5637F05	I.A.2	2-hydroxyhepta-2,4-diene-1,7-dioateisomerase/5-carboxymethyl-2-oxo-hex-3-ene-1,7-

			dioatedecarboxylase
**XAC4188**	A0UV6821D06	IX	RTS beta protein
**XAC4189**	A0UE6759H04	VIII.A	conserved hypothetical protein
**XAC4190**	A0QR6752D12	V.A.3	fucose permease
**XAC4192**	A0CE6831A06	VIII.A	conserved hypothetical protein
**XAC4193**	A0QR5704D01	I.D.1	two-component system, sensor protein
**XAC4194**	A0AM1424F01	VIII.A	conserved hypothetical protein
**XAC4195**	A0UE6759E06	IV.C	NdvB protein
**XAC4196**	A0JE5637C08	V.A.7	cation symporter
**XAC4197**	A0JJ0133G11	I.A.2	gluconokinase
**XAC4198**	A0CE6344H02	VIII.B	hypothetical protein
**XAC4199**	A0UV9746F02	I.C.3	polyvinylalcohol dehydrogenase
**XAC4200**	A0UV6846F06	VIII.C	Xanthomonas conserved hypothetical protein
**XAC4202**	A0UV9805G09	VIII.C	Xanthomonas conserved hypothetical protein
**XAC4204**	A0QR6804E08	VIII.A	conserved hypothetical protein
**XAC4206**	A0UV9710E01	VIII.B	hypothetical protein
**XAC4210**	A0QR6729H03	III.B.4	glycyl-tRNA synthetase beta chain
**XAC4211**	A0AC6827H07	III.B.4	glycyl-tRNA synthetase alpha chain
**XAC4212**	A0JJ0101H09	VII.H	general secretory pathway relatedprotein
**XAC4213**	A0UV6739E10	II.A.4	shikimate kinase
**XAC4214**	A0UT9849C09	II.B.1	GMP synthase
**XAC4216**	A0QR6803G02	V.A.6	sec-independent protein translocase
**XAC4217**	A0QR6728C09	IV.A.1	SEC-independent protein translocase
**XAC4219**	A0UE6759D02	VIII.A	conserved hypothetical protein
**XAC4220**	A0UV6712B04	II.D.12	ferrochelatase
**XAC4221**	A0UE6759D03	IX	hydrolase
**XAC4222**	A0UV6844G10	VIII.A	conserved hypothetical protein
**XAC4223**	A0QH6713H07	VIII.A	conserved hypothetical protein
**XAC4225**	A0UV6734F12	I.A.2	xylose isomerase
**XAC4226**	A0UV6735B12	I.D.2	sal operon transcriptional repressor
**XAC4227**	A0QR6491H08	I.A.1	alpha-glucuronidase
**XAC4228**	A0CE5420A10	IV.C	sialic acid-specific 9-O-acetylesterase
**XAC4229**	A0QR5512C08	I.D.4	starvation sensing protein
**XAC4230**	A0CE5422A09	I.A.1	xylosidase/arabinosidase
**XAC4231**	A0JJ0707A01	VII.D	glucan 1,4-beta-glucosidase
**XAC4232**	A0QR6804A07	I.A.2	mannitol dehydrogenase
**XAC4233**	A0UV9905A10	VII.C	bleomycin resistance protein
**XAC4234**	A0UV9748A07	VIII.B	hypothetical protein
**XAC4235**	A0UT6816D03	VIII.B	hypothetical protein
**XAC4236**	A0UV6709C11	IX	ring canal kelch - like protein
**XAC4241**	A0UT6815G11	II.D.12	glutamate-1-semialdehyde aminotransferase (aminomutase)
**XAC4243**	A0UT9891A10	I.A.2	L-fuculose-phosphate aldolase
**XAC4244**	A0CE6829F03	III.D.1	xylulose kinase
**XAC4245**	A0QH6309D04	VIII.B	hypothetical protein
**XAC4248**	A0QR6730B10	I.A.2	gluconolactonase precursor
**XAC4250**	A0RN1193G11	I.A.1	beta-galactosidase
**XAC4253**	A0QH6380C04	VIII.A	conserved hypothetical protein
**XAC4254**	A0UV6210E03	I.A.1	xylanase
**XAC4255**	A0UV5307E10	V.A.3	hexuranate transporter
**XAC4256**	A0RN1630G06	V.A.7	TonB-dependent receptor
**XAC4260**	A0UT6814G02	VIII.C	Xanthomonas conserved hypothetical protein
**XAC4261**	A0QR6705F02	VIII.B	hypothetical protein
**XAC4263**	A0UV9872G02	VIII.B	hypothetical protein
**XAC4263**	genomic_DNA	VIII.B	hypothetical protein

**XAC4265**	A0AM1405E08	VIII.B	hypothetical protein
**XAC4267**	A0QR6802B03	VIII.C	Xanthomonas conserved hypothetical protein
**XAC4273**	A0AR1060F11	VII.F	OmpA-related protein
**XAC4274**	A0QR6729C07	VII.F	OmpA-related protein
**XAC4275**	A0UE6378D11	II.D.17	tryptophan halogenase
**XAC4278**	A0JJ1553C07	VIII.A	conserved hypothetical protein
**XAC4279**	A0QR9739A10	VIII.C	Xanthomonas conserved hypothetical protein
**XAC4283**	A0UV6435H03	I.D.3	sensor histidine kinase
**XAC4285**	A0AC6823F05	VIII.C	Xanthomonas conserved hypothetical protein
**XAC4294**	A0QR6409B04	VIII.C	Xanthomonas conserved hypothetical protein
**XAC4296**	A0UT5316D03	IX	transglycolase/epimerase
**XAC4297**	A0JJ1578A09	VIII.A	conserved hypothetical protein
**XAC4299**	A0QR6755F11	VIII.A	conserved hypothetical protein
**XAC4300**	A0JJ1178E07	VIII.C	Xanthomonas conserved hypothetical protein
**XAC4302**	A0UV6433B10	II.D.2	GTP cyclohydrolase I
**XAC4304**	A0UT9846G08	VIII.C	Xanthomonas conserved hypothetical protein
**XAC4305**	A0QR9767F09	VII.C	fusaric acid resistance protein
**XAC4307**	A0JJ1072E02	VIII.A	conserved hypothetical protein
**XAC4308**	A0QR6768D08	V.A.7	dicarboxylate transport protein
**XAC4313**	A0QR6770B03	VIII.A	conserved hypothetical protein
**XAC4314**	A0UV1358B01	VI.B	plasmid stability protein
**XAC4315**	A0AC6440D02	VI.B	plasmid stability protein
**XAC4316**	A0UE9889C04	VIII.A	conserved hypothetical protein
**XAC4319**	A0QR5701E04	VIII.C	Xanthomonas conserved hypothetical protein
**XAC4323**	A0AM1403G12	VI.C	ISxac3 transposase
**XAC4324**	A0EC1462C07	VIII.A	conserved hypothetical protein
**XAC4326**	A0AC6827D10	I.A.2	urea amidolyase
**XAC4327**	A0JE1321A12	I.A.2	urea amidolyase
**XAC4328**	A0RN1548D09	VI.C	ISxac1 transposase
**XAC4329**	A0QR6755A05	VIII.A	conserved hypothetical protein
**XAC4330**	A0QR5904D07	VIII.C	Xanthomonas conserved hypothetical protein
**XAC4331**	A0QR9825A08	VIII.A	conserved hypothetical protein
**XAC4334**	A0QH6732G02	VIII.B	hypothetical protein
**XAC4336**	A0JJ1440C09	III.A.3	exodeoxyribonuclease V beta chain
**XAC4339**	A0AC9896A06	VII.G	toluene tolerance protein
**XAC4342**	A0QR5521D08	VII.G	toluene tolerance protein
**XAC4344**	A0UV6819F06	III.D.3	lipoprotein
**XAC4345**	A0QR6840C01	IX	enterocin biosynthesis related protein
**XAC4352**	A0CE6314E07	VII.C	glutathione S-transferase
**XAC4354**	A0JJ1037C10	V.A.1	amino acid transporter
**XAC4358**	A0JE1210H03	VIII.A	conserved hypothetical protein
**XAC4359**	A0QR6802C08	I.D.2	sugar diacide regulator
**XAC4360**	A0QR9717C04	I.A.2	glycerate kinase
**XAC4362**	A0CE6828A09	III.C.3	dipeptidase
**XAC4364**	A0QR5904F03	I.C.3	oxidoreductase
**XAC4365**	A0QH6713H05	V.A.7	export protein
**XAC4367**	A0UV9871G04	III.D.2	glycerophosphoryl diester phosphodiesterase
**XAC4368**	A0RN1551F10	V.A.4	TonB-dependent receptor
**XAC4369**	A0UT6386F11	I.B.9	phosphatase precursor
**XAC4370**	A0AC6827A04	VII.C	thiophene and furan oxidation protein
**XAC4371**	A0QH6813E01	IX	polysaccharide deacetylase
**XAC4372**	A0UV6712D07	IV.A.1	60kDa inner-membrane protein
**XAC4373**	A0QR5704E10	III.B.4	ribonuclease P, protein component
**XACa0001**	A0UV6845A08	VIII.A	conserved hypothetical protein

**XACa0005**	A0QR6729B12	VI.C	ISxac2 transposase
**XACa0009**	A0UE6718C05	VI.C	ISxac3 transposase
**XACa0010**	A0CE6505A02	VI.C	ISxac3 transposase
**XACa0011**	A0QR5701C07	VIII.B	hypothetical protein
**XACa0012**	A0CE6740A04	VIII.B	hypothetical protein
**XACa0027**	A0UV6818E02	VI.B	plasmid stable inheritance protein K
**XACa0028**	A0AC0115A01	VI.B	plasmid stable inheritance protein I
**XACa0029**	A0QR6702H02	VI.C	transposase
**XACa0032**	A0QR6752G10	VIII.A	conserved hypothetical protein
**XACa0034**	A0UV6433D05	VI.C	Tn5045 transposase
**XACa0041**	A0QR6704E11	VI.B	partition protein A
**XACa0042**	A0UV6766A11	VI.B	KfrA protein
**XACb0002**	A0UE6759E09	VIII.B	hypothetical protein
**XACb0003**	A0QR6705H11	VIII.B	hypothetical protein
**XACb0011**	A0JJ0104E03	VII.A	avirulence protein
**XACb0015**	genomic_DNA	VII.A	avirulence protein
**XACb0019**	genomic_DNA	VI.B	partition protein A
**XACb0020**	genomic_DNA	VIII.B	hypothetical protein
**XACb0021**	genomic_DNA	VIII.B	hypothetical protein
**XACb0022**	genomic_DNA	VIII.A	conserved hypothetical protein
**XACb0023**	genomic_DNA	VIII.A	conserved hypothetical protein
**XACb0024**	genomic_DNA	VIII.B	hypothetical protein
**XACb0025**	genomic_DNA	VIII.B	hypothetical protein
**XACb0026**	genomic_DNA	VIII.B	hypothetical protein
**XACb0027**	genomic_DNA	VIII.B	hypothetical protein
**XACb0028**	genomic_DNA	VIII.B	hypothetical protein
**XACb0029**	genomic_DNA	VIII.B	hypothetical protein
**XACb0030**	A0QR6702F05	VI.B	TrwB protein
**XACb0030**	genomic_DNA	VI.B	TrwB protein
**XACb0031**	A0RN1459D02	VI.B	TrwC protein
**XACb0032**	A0QR6803F06	VIII.A	conserved hypothetical protein
**XACb0033**	genomic_DNA	VIII.A	conserved hypothetical protein
**XACb0034**	A0QR5407G02	VIII.B	hypothetical protein
**XACb0034**	genomic_DNA	VIII.B	hypothetical protein
**XACb0035**	A0JJ0104F07	VIII.B	hypothetical protein
**XACb0035**	genomic_DNA	VIII.B	hypothetical protein
**XACb0036**	A0QR9864G07	VII.H	VirB1 protein
**XACb0037**	A0UT6815B09	VII.H	VirB11 protein
**XACb0037**	genomic_DNA	VII.H	VirB11 protein
**XACb0038**	A0UV6735F02	VII.H	VirB10 protein
**XACb0039**	genomic_DNA	VII.H	VirB9 protein
**XACb0040**	A0UV9872A07	VII.H	VirB8 protein
**XACb0041**	genomic_DNA	VII.H	VirB6 protein
**XACb0041**	A0QR6730A05	VII.H	VirB6 protein
**XACb0041**	genomic_DNA	VII.H	VirB6 protein
**XACb0042**	A0QR6840E02	VIII.B	hypothetical protein
**XACb0042**	genomic_DNA	VIII.B	hypothetical protein
**XACb0043**	A0AM1594G01	VIII.B	hypothetical protein
**XACb0045**	genomic_DNA	VII.H	VirB4 protein
**XACb0045**	A0UE6759E05	VII.H	VirB4 protein
**XACb0046**	A0UV6733D10	VII.H	VirB3 protein
**XACb0048**	A0CE6743C02	VIII.B	hypothetical protein
**XACb0049**	genomic_DNA	VIII.B	hypothetical protein
**XACb0051**	A0UV1356C11	VI.C	ISxac2 transposase

**XACb0052**	A0UT6817C08	VI.B	partition gene repressor
**XACb0053**	A0CE6742F12	VI.B	partition protein B
**XACb0054**	A0AC6827F05	VI.B	partition protein A
**XACb0056**	A0UV6821D10	VI.B	replication protein A
**XACb0057**	A0QR6801F04	VIII.A	conserved hypothetical protein
**XACb0059**	A0QR5704F12	VII.H	virulence associated protein
**XACb0060**	A0QR5702B12	VII.H	virulence plasmid protein
**XACb0062**	A0UV6762F12	VI.C	ISxac3 transposase
**XACb0064**	A0UV6736F09	VIII.B	hypothetical protein
**XACb0067**	A0AC1387F05	VI.C	Tn5045 transposase
**XACb0072**	A0QR5407H04	VI.B	resolvase
